# Pre‐Constructed Mechano‐Electrochemical Adaptive Solid Electrolyte Interphase to Enhance Li^+^ Diffusion Kinetics and Interface Stability for Chemically Prelithiated SiO Anodes

**DOI:** 10.1002/advs.202515555

**Published:** 2025-10-16

**Authors:** Zhan Wang, Shuang Li, Yun Zheng, Yinan Liu, Qilin Feng, Chencheng Xu, Quanchao Zhuang, Zhicheng Ju, Jiangmin Jiang, Huaiyu Shao, Xiaogang Zhang

**Affiliations:** ^1^ Jiangsu Province Engineering Laboratory of High Efficient Energy Storage Technology and Equipments, School of Materials Science and Physics China University of Mining and Technology Xuzhou 221116 China; ^2^ Guangdong‐Hong Kong‐Macau Joint Laboratory for Photonic‐Thermal‐Electrical Energy Materials and Devices, Institute of Applied Physics and Materials Engineering University of Macau Macau 999078 China; ^3^ Jiangsu Key Laboratory of Electrochemical Energy Storage Technology, College of Material Science and Engineering Nanjing University of Aeronautics and Astronautics Nanjing 210016 China

**Keywords:** chemical prelithiation, initial Coulombic efficiency, lithium‐ion batteries, SiO anode, solid electrolyte interphase

## Abstract

The development of silicon monoxide (SiO) anode in high‐energy lithium‐ion batteries (LIBs) is challenged by low initial Coulombic efficiency (ICE) and significant volume expansion. Although chemical prelithiation can enhance the ICE of SiO, it inevitably induces volume expansion in advance and suffers the inferior air stability. Herein, a chemical prelithiation‐mediated strategy is proposed that pre‐constructs a mechano‐electrochemical adaptive solid electrolyte interphase (SEI) through the spontaneous reaction of ammonium hexafluorozirconate (Ah) with the chemically prelithiated SiO anode (Pr‐SiO). The mechano‐electrochemical adaptive SEI, enriched with LiF, Li_3_N, and ZrO_2_ components, exhibits a unique structure of “rigid inside and flexible outside” to enhance electrochemical reaction kinetics and mechanical durability. The Pr‐SiO with the adaptive SEI (Ah‐Pr‐SiO) possesses high ICE (99.4%), fast Li^+^ diffusion kinetics, and superior cycle stability (1435.8 mAh g^−1^ after 200 cycles). Notably, the designed Ah‐Pr‐SiO reveals high hydrophobicity and air stability, leading to feasible industrial compatibility. The assembled pouch cell (LiNi_0.8_Co_0.1_Mn_0.1_O_2_//Ah‐Pr‐SiO) exhibits stable cycling with a high energy density (346.6 Wh kg^−1^). This work provides a novel chemical prelithiation‐mediated pre‐constructed SEI strategy, offering the possibility of designing an advanced SEI for Si‐based anodes toward high energy density long‐life lithium‐ion batteries.

## Graphical abstract

Silicon oxide (SiO) is emerging as the most advanced anode material. Herein, we propose a chemical prelithiation‐mediated strategy for SiO, which improves initial Coulombic efficiency (ICE) and pre‐constructs a mechano‐electrochemical adaptive solid electrolyte interphase (SEI). Notably, the mechano‐electrochemical adaptive SEI enriched with LiF, Li_3_N, and ZrO_2_ components exhibits outstanding electrochemical reaction kinetics and mechanical durability.

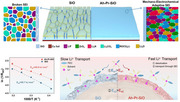

## 1 Introduction

With the increasing global energy demand, rechargeable batteries, particularly lithium‐ion batteries (LIBs), are continuously advancing to achieve higher specific capacities and lower costs.^[^ ^1^, ^2^, ^3^, ^4^, ^5^ ^]^ Silicon monoxide (SiO) is a promising anode material due to its relatively high theoretical specific capacity (2680 mAh g^−1^) and abundant availability.^[^ ^6^, ^7^, ^8^, ^9^, ^10^ ^]^ However, SiO suffers from severely active lithium loss (>20%) originating from irreversible Li_2_O and lithium silicates (Li_x_SiO_y_) generation during initial lithiation for solid electrolyte interphase (SEI) formation.^[^ ^11^, ^12^ ^]^ The irreversible lithium consumption leads to a significantly reduced initial Coulombic efficiency (ICE) (<80%) and severely restricts attainable energy density, which hinders the development of the SiO anode.^[^ ^13^, ^14^ ^]^ Therefore, developing effective strategies to overcome the low ICE is essential to unlock the full potential of SiO anodes in high‐energy LIBs.^[^ ^15^, ^16^ ^]^

Up to now, various strategies have been proposed to address the challenge of low ICE for the SiO anode.^[^ ^17^, ^18^, ^19^, ^20^ ^]^ Prelithiation strategy is widely regarded as the most effective method for compensating irreversible lithium loss through advanced lithium replenishment,^[^ ^21^, ^22^, ^23^ ^]^ such as direct contact prelithiation,^[^ ^24^, ^25^, ^26^ ^]^ electrochemical prelithiation,^[^ ^27^, ^28^ ^]^ prelithiation additives,^[^ ^29^, ^30^, ^31^ ^]^ etc. Unfortunately, these prelithiation methods exhibit several limitations, including the safety of highly active lithium metal, the controllability of the degree of prelithiation, and the complexity of process operation.^[^ ^32^ ^]^ Notably, chemical prelithiation has garnered significant attention recently due to its advantages of controllability, simplicity, and low cost.^[^ ^33^, ^34^ ^]^ By direct reactions between highly active reducing agents and electrode materials, chemical prelithiation enables efficient lithium replenishment for the SiO anode and improves the ICE.^[^ ^35^, ^36^, ^37^ ^]^ However, a key issue needs to be emphasized, while chemical prelithiation can achieve the replenishment of active lithium, it inevitably affects the cycling stability and air stability of SiO anodes, becoming a challenge that must be addressed for practical applications.^[^ ^38^, ^39^, ^40^, ^41^, ^42^ ^]^

To eliminate the adverse effect of stability in chemically prelithiated SiO anodes, one must understand the underlying mechanism of the active lithium replenishment. For the SiO anode, the chemically prelithiated replenishment lithium reactive with SiO to form amorphous and reversible Li_x_Si alloys, leading to significant volume expansion and a rapid increase in internal stress.^[^ ^43^, ^44^ ^]^ Compared to the lithiation process during cycling, the lithiation of chemical prelithiation is confined to the electrode surface in contact with the solution, which tends to cause uneven lithium distribution and localized stress concentration.^[^ ^38^ ^]^ If the SEI lacks sufficient strength to withstand the localized stress changes induced by uneven lithiation, lithium loss due to SEI reformation, and the mechanical failure of lithiated material‐contact interphase will increase, ultimately impairing the long‐term cycle stability of SiO anodes.^[^ ^39^, ^40^ ^]^ Moreover, due to the intrinsic material properties of SiO, its low ionic conductivity directly slows electrochemical kinetics, thereby reducing its rate capability.^[^ ^45^, ^46^, ^47^ ^]^ In addition, as the surface of SiO is rich in active lithium after chemical prelithiation, it has a high chemical reactivity with air and moisture, making it susceptible to erosion by oxygen and water, which leads to reduced industrial compatibility. Hence, it is imperative to achieve the high ICE of the SiO anode, together with simultaneously design a unique SEI structure with superior ionic conductivity and high interface stability.

Herein, we propose a chemical prelithiation‐mediated strategy to in situ pre‐construct a mechano‐electrochemical adaptive SEI by spontaneous reacting of prelithiated SiO (Pr‐SiO) with ammonium hexafluorozirconate (Ah), which is enriched with LiF, Li_3_N, and ZrO_2_ components. Owing to the favourable mechanical properties and stability of LiF and ZrO_2_, which effectively accommodates the stress fluctuations and volume expansion of Pr‐SiO, and the high Li⁺ flux of Li_3_N significantly improves its ionic conductivity during the cycle process. In addition, the mechano‐electrochemical adaptive SEI exhibits a unique structure of “rigid inside and flexible outside,” which provides enhanced Li^+^ diffusion kinetics and extraordinary mechanical durability. Notably, the pre‐constructed inorganic‐rich interphase exhibits ultra‐hydrophobicity, ensuring the prelithiated SiO features air stability with higher industrial compatibility. As expected, the chemically prelithiated SiO anode with mechano‐electrochemical adaptive SEI (Ah‐Pr‐SiO) achieves an impressive ICE of 99.4%, along with favourable cycle stability (1435.8 mAh g^−1^ after 200 cycles) and superior rate capability. As a practical application, the assembled lithium‐ion full batteries (LiFePO_4_//Ah‐Pr‐SiO) deliver a high ICE (91.2%), and the assembled pouch cell (LiNi_0.8_Co_0.1_Mn_0.1_O_2_//Ah‐Pr‐SiO) exhibits stable cycling with a high energy density (346.6 Wh kg^−1^).

## 2 Results and Discussion

**Figure 1:**
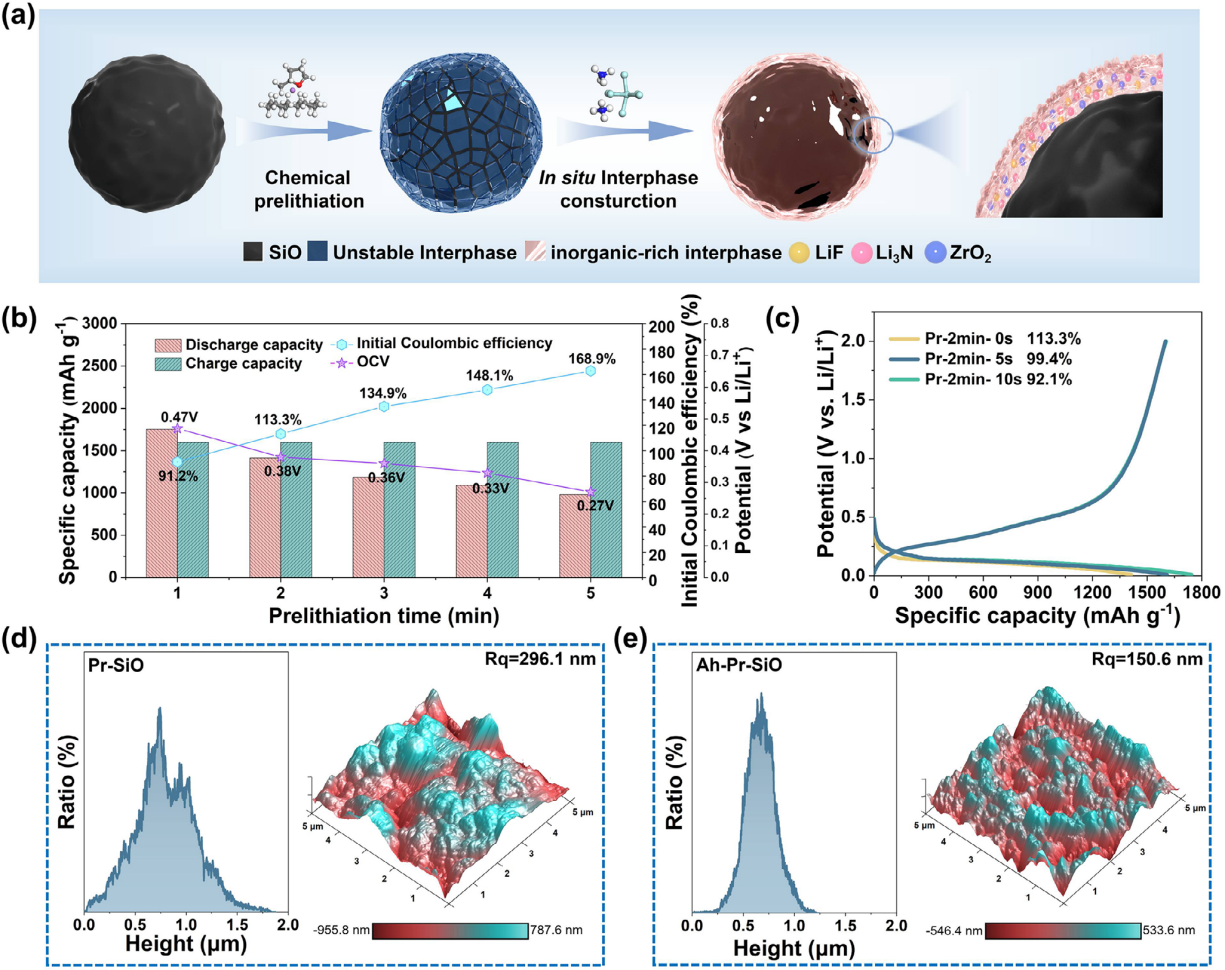
a) Schematic diagram of chemical prelithiation and in situ inorganic‐rich interphase pre‐construction. b) The relationship between OCV/ICE and prelithiation time. c) Initial charge/discharge curves of Pr‐2min‐SiO at various reaction times. The 3D AFM morphology and the ratio for the height of d) Pr‐SiO and e) Ah‐Pr‐SiO, respectively.

The chemical prelithiation and interphase pre‐construction processes for the SiO anode are shown in **Figure** **1**a. In particular, a controllable pre‐constructed inorganic‐rich interphase strategy was used based on the in situ spontaneous reaction between Ah and active lithium derived from Pr‐SiO. Micrometer‐scale SiO particles, which were selected as the research subject, exhibit an amorphous state with particle sizes ranging from 3 to 12 µm (Figures S1 and S2, Supporting Information). The pristine SiO was first prelithiated by immersion in a chemical prelithiation solution, resulting in the formation of Pr‐SiO. Subsequently, a spontaneous chemical reaction occurred between the excessive active lithium on the surface of Pr‐SiO and the Ah reagent, leading to the in situ construction of an inorganic‐rich interphase, which can be spontaneously transformed to realize the pre‐construction of SEI without charge–discharge treatment.

For chemical prelithiation of SiO anode, the selection of redox potential of the chemical prelithiation reagents is crucial. The feasibility of chemical prelithiation was analyzed by density functional theory (DFT) calculations (Figures S3 and S4, Supporting Information), and the biphenyl‐lithium/2‐methyltetrahydrofuran lithium‐arene complex (Li‐BP/2‐Me‐THF) was selected for the chemical prelithiation solution. In this system, the Li^+^ in Li‐BP can combine with the oxygen atom on the ether group in 2‐Me‐THF to form a stable complex, which has the highest HOMO energy level and indicates a high reduction capacity with low redox potential.^[^ ^48^ ^]^ Cyclic voltammetry (CV) with dQ/dV curves revealed that the redox potential of Li‐BP/2‐Me‐THF solution (0.085 V) is lower than the lithiation potential of the SiO (0.22 V), which demonstrates the feasibility of Pr‐SiO anode (Figure S5, Supporting Information). The controllability of chemical prelithiation was explored by comparing the ICE, initial open‐circle voltages (OCV), and discharge–charge curves of the Pr‐SiO (Figure 1b; Figure S6, Supporting Information). With an increase in prelithiated time and the continuous replenishment of active Li^+^, the ICE of Pr‐SiO increases, and the OCV decreases. This demonstrates a positive correlation between prelithiation efficiency and prelithiation duration, and the chemical prelithiation process can be controlled.

Considering the consumption of active lithium during the spontaneous reaction, the degree of prelithiation process needs to be slightly excessive, thus Pr‐SiO, conducted for two minutes with an ICE of 113.3% was selected for the spontaneous reaction to construct an inorganic‐rich interphase. Since dimethyl sulfoxide (DMSO) is a polar non‐protonic solvent, it can dissociate complex salts into their corresponding ionic groups.^[^ ^49^ ^]^ Taking into account component introduction and industrial costs, Ah, which can simultaneously provide NH_4_^+^ and ZrF_6_^2−^, was selected as the solvent (Table S1, Supporting Information). Ah dissociates into NH_4_^+^ and ZrF_6_^2−^ when dissolved in DMSO at room temperature and pressure upon dissolution and reacts with the lithiation products on the Pr‐SiO surface to form Li_3_N, LiF, and ZrO_2_ (Figure S7, Supporting Information). As the immersion time increased, the reaction became more pronounced, accompanied by the formation of white bubbles on the surface of Pr‐SiO (Figure S8, Supporting Information). Notably, the ICE of Ah‐Pr‐SiO after interfacial reaction (10 s) dropped to 92.1% (Figure 1c), indicating that the spontaneous reaction resulted in excessive consumption of active lithium and a reduction in ICE. In comparison, the Ah‐Pr‐SiO exhibits a high ICE of 99.4% after an interfacial reaction with 5 s, suggesting that controllability of the interphase construct can be achieved by varying the reaction time to realize optimal reaction conditions. Under these conditions, excessive consumption of active lithium caused by prolonged chemical prelithiation can be avoided, and sufficient active lithium can be ensured to participate in the formation of the cathode component.

**Figure 2:**
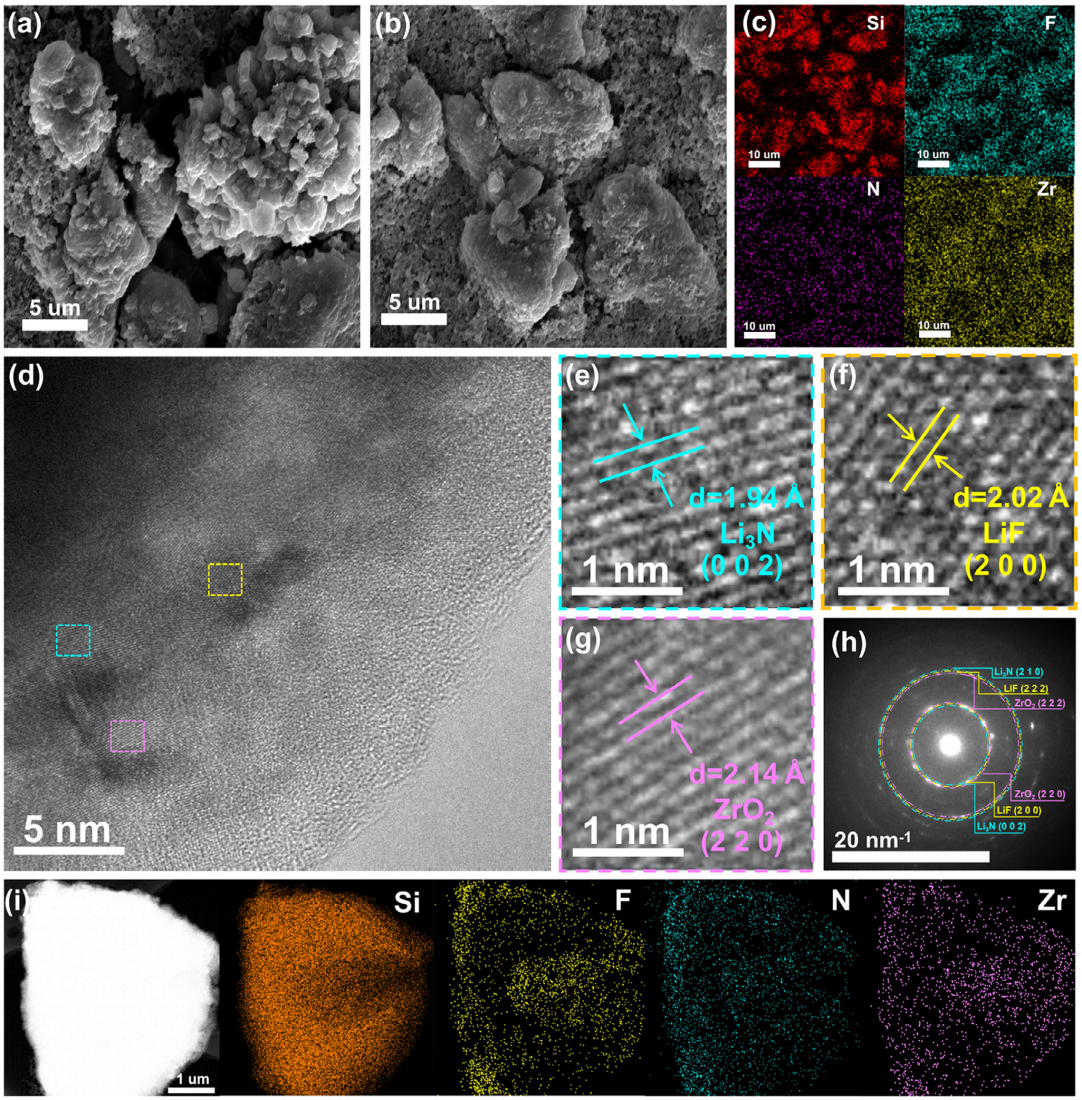
SEM image of a) Pr‐SiO and b) Ah‐Pr‐SiO. c) SEM‐EDS mapping of Ah‐Pr‐SiO. d) HR‐TEM images of the Ah‐Pr‐SiO. e–g) lattice stripes of Li_3_N, LiF and ZrO_2_. h) SAED image and i) HR‐TEM‐EDS mapping of Ah‐Pr‐SiO.

The homogeneity and denseness of the pre‐constructed interphase were detected by atomic force microscopy (AFM), which evaluates surface morphology by measuring surface roughness (Ra). The surface roughness of Ah‐Pr‐SiO (Ra = 150.6 nm) is significantly lower than that of Pr‐SiO (Ra = 296.1 nm), indicating a more uniform surface has formed after spontaneous reaction. The height distribution of Pr‐SiO and Ah‐Pr‐SiO further highlights differences in surface roughness (Figure 1d,e). Compared to Ah‐Pr‐SiO, Pr‐SiO exhibits a more dispersed height ratio, indicating an inhomogeneous surface after chemical prelithiation. Scanning electron microscopy (SEM) was employed to observe the surface morphology of the three samples. In contrast to the relatively smooth surface of pristine SiO particles, Pr‐SiO exhibits a rougher surface with an obvious volume expansion, whereas Ah‐Pr‐SiO demonstrates a uniformly dense and compact morphology. (**Figure** **2**a,b; Figure S9, Supporting Information). This phenomenon can be attributed to the inorganic‐rich interphase constructed after the spontaneous reaction effectively mitigates the massive morphological deformation of Pr‐SiO due to the volume expansion induced by the gradual replenishment of lithium.

To better analyze the chemical constituents of the pre‐constructed inorganic‐rich interphase, X‐ray photoelectron spectroscopy (XPS) was conducted on SiO, Pr‐SiO, and Ah‐Pr‐SiO electrodes before the cycle process. The appearance of lithium peaks in the Li 1s spectra of Pr‐SiO and Ah‐Pr‐SiO confirms the successful introduction of active lithium (Figure S10, Supporting Information). As shown in Figure S11a,b (Supporting Information), the F 1s and N 1s spectra of Ah‐Pr‐SiO show characteristic peaks at 684.9 eV and 398.1 eV, which are attributed to LiF and Li_3_N, respectively.^[^ ^50^, ^51^ ^]^ As a comparison, the absence of F 1s and N 1s peaks in untreated Pr‐SiO indicates that the LiF and Li_3_N are exclusively formed through the spontaneous chemical reaction between Ah and excessive active lithium. The Zr 3d spectra exhibit two characteristic peaks at 181.6 and 184.1 eV, corresponding to Zr 3d^3/2^ and Zr 3d^5/2^, respectively (Figure S11c, Supporting Information). The energy gap between these peaks is ≈2.5 eV, and their relative intensity ratio is ≈1.5, which is the typical characteristic of ZrO_2_.^[^ ^52^ ^]^ The C 1s and O 1s spectra reveal that the interfacial construction did not alter the pristine composition of Pr‐SiO apart from the introduced components (LiF, Li_3_N, and ZrO_2_), confirming that the in situ spontaneous reaction was mild and preserved the integrity of the original electrode (Figure S12, Supporting Information). Meanwhile, energy dispersive X‐ray spectroscopy (SEM‐EDS) shows that Si, F, N, and Zr elements are uniformly distributed on the surface of Ah‐Pr‐SiO (Figure 2c; Figure S13, Supporting Information).

High‐resolution transmission electron microscopy (HR‐TEM) was utilized to further elucidate the microstructure and specific composition of the pre‐constructed inorganic‐rich interphase. As shown in Figure S14 (Supporting Information), SiO forms an uneven lithiation interface layer after chemical prelithiation, with crystal lattice stripes of the lithiation product Li‐Si distributed throughout ((3 1 2), 0.205Å). In comparison, the Ah‐Pr‐SiO exhibits uniform and distinct lattice fringes of 1.94, 2.02, and 2.18 Å, corresponding to the (0 0 2), (2 0 0), and (2 2 0) crystal planes of Li_3_N, LiF, and ZrO_2_, respectively (Figure 2d–g; Figure S15, Supporting Information). The presence and distribution of these inorganic components were confirmed by selected area electron diffraction (SAED) patterns and HR‐TEM‐EDS mapping (Figure 2h,i). Therefore, these characterization results demonstrate the successful construction of an interphase enriched with LiF, Li_3_N, and ZrO_2_ on the surface of the Pr‐SiO electrode through the in situ spontaneous reaction with the Ah reagent.

**Figure 3:**
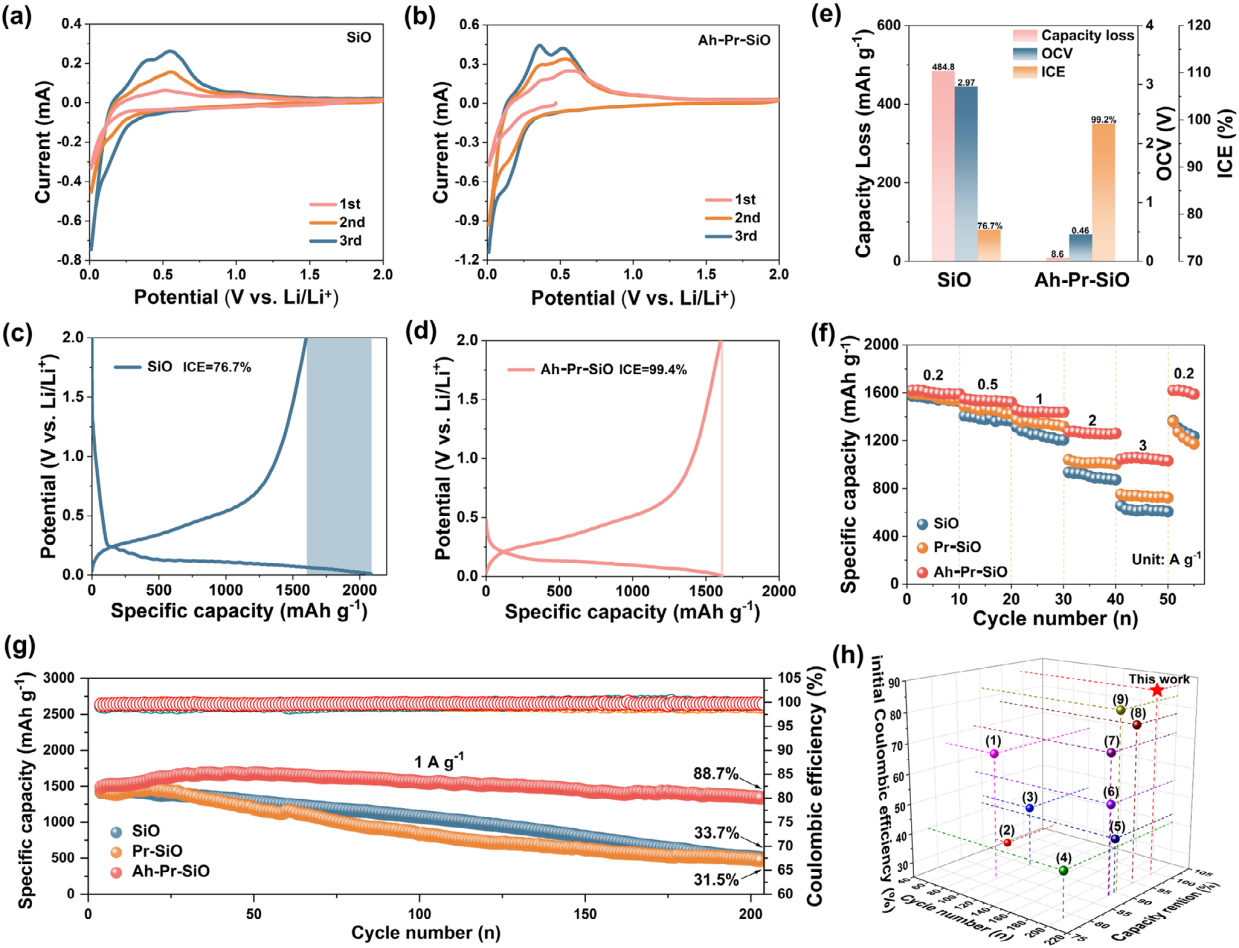
CV curves a,b), and Initial charge/discharge curves c,d) of SiO and Ah‐Pr‐SiO. e) Corresponding data for the capacity loss, ICE, and OCV of SiO and Ah‐Pr‐SiO. f) Rate performance of SiO, Pr‐SiO, and Ah‐Pr‐SiO, respectively. g) Cycling performance of SiO, Pr‐SiO, and Ah‐Pr‐SiO, and h) Comparison of ICE and cycling stability of this work with other reported SiO‐based anodes.

To evaluate the influence introduced by the pre‐constructed inorganic‐rich interphase on the practical performance of the SiO, Pr‐SiO, and Ah‐Pr‐SiO were investigated (**Figure** **3**). The electrochemical behavior and prelithiation degree were assessed by CV and initial charge/discharge curves. Compared with SiO, the OCV of Ah‐Pr‐SiO was significantly reduced to 0.46 V, which was much lower than the SEI formation voltage of 1.25 V due to the effective lithium replenishment, demonstrating the pre‐construction of SEI after exposing to the electrolyte (Figure 3a,b).^[^ ^53^ ^]^ The ICE of Ah‐Pr‐SiO improved significantly from 76.7% to 99.4%, and the capacity loss decreased from 485.1 to 8.6 mAh g^−1^, further confirming the successful replenishment of active lithium (Figure 3c,d). Furthermore, compared with Pr‐SiO, Ah‐Pr‐SiO exhibits similar redox peaks in the CV curve, indicating that the chemical prelithiation‐mediated interface pre‐construction did not alter the electrochemical behavior (Figure S16, Supporting Information). Figure 3e demonstrates the values reflecting the effect of prelithiation, proving that the chemical prelithiation‐mediated strategy can efficiently enhance the ICE of SiO anode. DC polarization tests were used to investigate the stability of the constructed interphase. The interfacial properties were qualitatively evaluated based on the stabilization degree of the leakage current after discharging the cells uniformly to 0.01 V and maintaining the voltage constant. It can be found that the Ah‐Pr‐SiO exhibits a smaller leakage current that stabilizes faster than SiO, indicating the formation of a more uniform and stable interphase (Figure S17, Supporting Information).

The enhancement of Li^+^ diffusion kinetics by the inorganic‐rich interphase was validated through electrochemical impedance spectroscopy (EIS) and rate performance tests. As shown in Figure S18 (Supporting Information), Ah‐Pr‐SiO displays the lowest interfacial impedance (R_SEI_ = 11.9 Ω) and charge‐transfer impedance (Rct = 4.7 Ω) among the three samples (Table S1, Supporting Information), confirming the fast Li^+^ transport ability of the pre‐constructed SEI. In particular, Ah‐Pr‐SiO exhibits a specific capacity of 1031.2 mAh g^−1^ at a high current density of 3 A g^−1^, which is much higher than SiO (606.7 mAh g^−1^) and Pr‐SiO (722.9 mAh g^−1^), respectively. After the current density was reset to 200 mA g^−1^, the reversible capacity of Ah‐Pr‐SiO is recovered to 1614.3 mAh g^−1^, demonstrating the superior rate capability (Figure 3f). Moreover, Ah‐Pr‐SiO exhibits impressive long‐term cycling stability, which delivers a specific capacity of 1435.8 mAh g^−1^ with a high capacity retention of 88.7% after 200 cycles at a current density of 1 A g^−1^. In contrast, SiO and Pr‐SiO exhibit the specific capacities of 570.1 and 538.7 mAh g^−1^ after 200 cycles, corresponding to the capacity retentions of 33.7% and 32.5%, respectively (Figure 3g; Figure S19, Supporting Information). It is shown that the design of pre‐constructed inorganic‐rich SEI layer can effectively supplement the active lithium loss and improve the ICE of SiO anode, as well as enhance the rate capability and cycle stability simultaneously. Figure 3h compares the electrochemical performance of the Ah‐Pr‐SiO anode to other published works, further revealing the outstanding electrochemical performance of our Ah‐Pr‐SiO (Table S3, Supporting Information).

**Figure 4:**
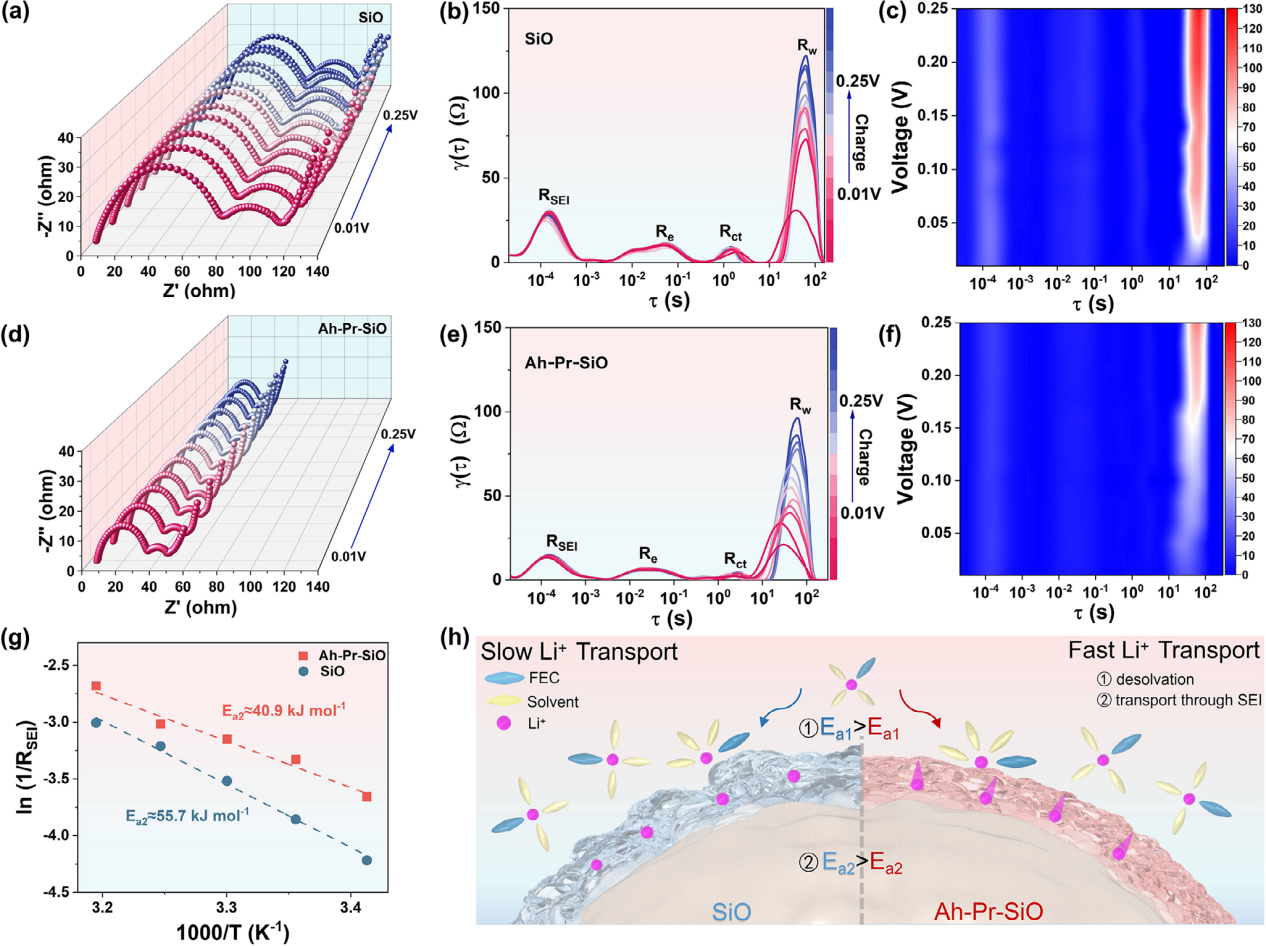
a) In situ impedance of SiO and d) Ah‐Pr‐SiO at initial cycling. DRT curves of b) SiO and e) Ah‐Pr‐SiO. In situ DRT contour plots of c) SiO and f) Ah‐Pr‐SiO monitored at initial cycling. g) Activation energies (Ea_2_) of Li^+^ transport through the SEI. h) Schematic of the enhanced Li^+^ transport facilitated by the SEI with inorganic‐rich components.

To investigate the effect of pre‐constructed SEI on Li^+^ diffusion kinetics during lithiation‐delithiation processes, in situ impedance tests were conducted. Both the charging and discharging processes of Ah‐Pr‐SiO exhibit the lowest electrochemical impedance (**Figure** **4**a,d; Figures S20 and S21a, Supporting Information). The distribution of relaxation times (DRT) of the charging process fitting shows that the fitted peaks were the interfacial impedance R_SEI_, contact impedance R_e_, charge transfer impedance R_ct_, and diffusion impedance R_w_, respectively (Figure 4b,e; Figure S21b,c, Supporting Information).^[^ ^54^, ^55^ ^]^ Notably, an additional peak R_i_ between R_e_ and R_ct_ can be observed for Pr‐SiO and Ah‐Pr‐SiO (Figure S22, Supporting Information), which is mainly composed of the lithium silicate salt phase and lithium‐silicon alloy phase formed after chemical prelithiation. The electrochemical impedance is smaller due to the lack of organic components generated by the decomposition of the electrolyte as compared to the SEI interphase formed for the initial discharge process of the battery. The low full‐potential impedance of Ah‐Pr‐SiO is attributed to the pre‐constructed interphase containing Li_3_N, which exhibits favorable electrochemical activity and facilitates high Li^+^ flux across the interphase. In addition, the R_SEI_ of SiO and Pr‐SiO increases with the charging process, while Ah‐Pr‐SiO remains constant. This is due to the pre‐constructed interphase being stable enough to reduce the side reaction that occurs when the electrolyte first contacts the electrode material. The all‐potential impedance and DRT simultaneously demonstrate the superior Li^+^ diffusion kinetics of the pre‐constructed SEI on the surface of Ah‐Pr‐SiO.

The ionic transport properties of Li^+^ across the solid electrolyte interphase (R_SEI_) and charge transfer resistance (R_ct_) were analyzed in situ variable‐temperature EIS (Figure S23a, Supporting Information). The activation energy associated with these processes was derived from the EIS data using the Arrhenius equation (Equation S1, Supporting Information). Notably, the activation energy of the desolvation process (E_a1_) of Ah‐Pr‐SO and Li^+^ transport through its SEI (E_a2_) was much lower than that for the SiO (41.1 vs 61.1 kJ mol^−1^ for E_a1_, 40.9 vs 55.7 kJ mol^−1^ for E_a2_) (Figure 4g,h; Figure S23b, Supporting Information). The E_a1_ improves the desolvation of Li^+^ on the SiO surface, and the E_a2_ can be advantageous for fast transport of Li^+^ within the SEI. The above advantages are due to the presence of Li_3_N, which has an affinity energy for Li^+^ with high ionic conductivity.^[^ ^56^ ^]^ The activation energy results further demonstrate that the Li^+^ transport kinetics have been significantly enhanced by the pre‐constructed interphase enriched with Li_3_N components, thereby improving the electrochemical activity of the SEI and rate capability.

The enhancement of the Li^+^ diffusion kinetics of Ah‐Pr‐SiO was further verified. The differences in the Li^+^ diffusion coefficient (D_k_) of the three samples were calculated and compared by GITT under different charging and discharging states, and the Li^+^ diffusion coefficient was calculated by Equation S2 (Supporting Information). In particular, Ah‐Pr‐SiO shows the highest D_k_ in both lithiation and delithiation processes, indicating that the modulation of the SEI layer contributes to the improvement of Li^+^ diffusion kinetics (Figures S24 and S25, Supporting Information). Based on the CV curves at different sweep speeds, the corresponding b‐values were calculated from Equation S3 (Supporting Information) to investigate the lithium storage behavior of Ah‐Pr‐SiO (Figures S26 and S27, Supporting Information). Typically, the *b* value between 0.5 and 1.0 indicates that the material has a hybrid lithium storage behavior of capacitive and diffusion‐controlled processes. The higher *b* value of Ah‐Pr‐SiO compared to SiO indicates that the pseudocapacitive reaction dominates the electrochemical process at the anode. The contribution of the two lithium storage behaviors can be calculated from Equation S4 (Supporting Information), in which Ah‐Pr‐SiO exhibits a greater pseudocapacitance contribution that progressively increases with the scan rate. This is attributed to the exceptional stability and rapid lithium‐ion diffusion kinetics of its mechanically‐adaptive electrochemical SEI interface, which prevents fragmentation and reorganization while enabling rapid lithium‐ion transport under high‐current conditions.^[^ ^57^, ^58^ ^]^ This trend reveals enhanced reaction kinetics at elevated current densities, thereby elucidating the superior rate performance of Ah‐Pr‐SiO at large current densities (Figures S28–S30, Supporting Information). To further monitor the Li^+^ conductivity through SEI, the EIS technique was adopted, and a symmetrical SiO//SiO configuration was used to eliminate the complex interactions of the Li metal electrodes in the SiO//Li half‐cell. The SiO electrodes in the symmetrical cell were obtained from a half‐cell after ten cycles to ensure stable SEI formation.^[^ ^59^ ^]^ As shown in Figure S31 (Supporting Information), it's shown that Ah‐Pr‐SiO has the lowest R_sei_ (15.5 Ω vs 20.4 Ω of Pr‐SiO and 35.7 Ω of Ah‐Pr‐SiO), fully demonstrating that mechano‐electrochemical adaptive SEI exhibits excellent Li^+^ conductivity.

**Figure 5:**
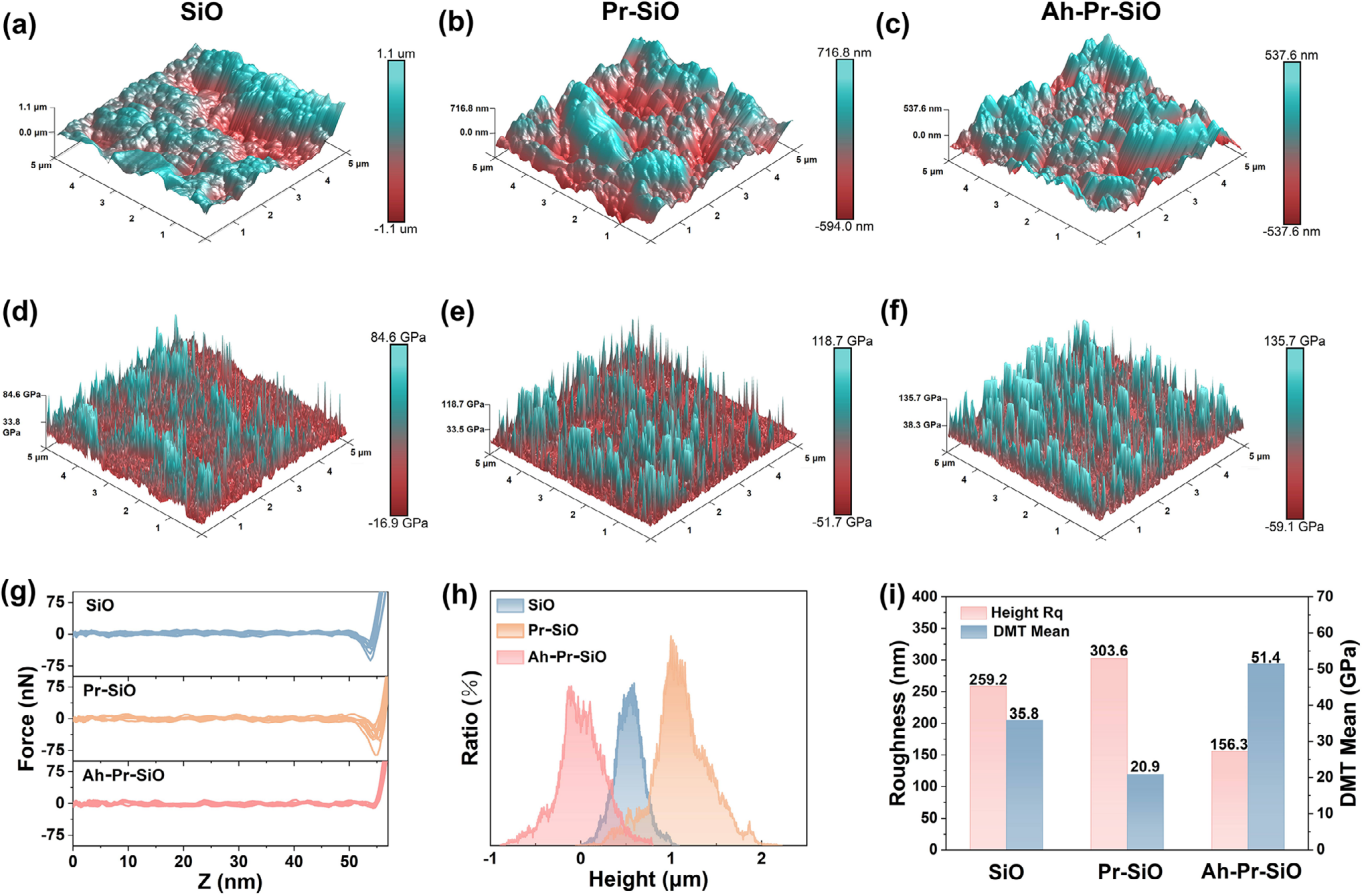
a–c). The 3D AFM morphology, d–f) Young's modulus, g) force‐displacement curves, h) corresponding ratio for the height, and i) corresponding data for the height Rq and DMT of the SiO, Pr‐SiO, and Ah‐Pr‐SiO, respectively.

The interphase characteristics and mechanical strength of the SEI layer on the SiO, Pr‐SiO, and Ah‐Pr‐SiO after cycling were investigated by AFM technology. In particular, the Rq of Ah‐Pr‐SiO (156.3 nm) is significantly lower than that of SiO (303.6 nm) and Pr‐SiO (259.2 nm) (**Figure** **5**a–c), indicating a more uniform surface after cycling. This improvement is attributed to the stable and dense SEI enriched with inorganic components, which effectively suppresses the fragmentation and reconstruction of the SEI during the cycling process. Young's modulus (DMT) measurements revealed that Ah‐Pr‐SiO possesses the highest modulus of 51.4 GPa, a substantial improvement compared to the 20.9 GPa of SiO (Figure 5d–f). Force curve analysis demonstrated that the SEI layer on Ah‐Pr‐SiO exhibits consistent jump contact and adhesion forces, confirming that the pre‐constructed interphase provides a uniform stress distribution (Figure 5g). This stands in stark contrast to Pr‐SiO, which experiences significant stress changes due to excessive lithiation during the prelithiation process. A comparison of the specific data for the three samples is shown in Figure 5h,i. Due to the superior mechanical strength and properties of LiF and ZrO_2_, the mechano‐electrochemical adaptive SEI of Ah‐Pr‐SiO with a high modulus and mechanical stability, enabling it to realize high ICE, meanwhile accommodating volume expansion, leading to superior cycle stability.

**Figure 6:**
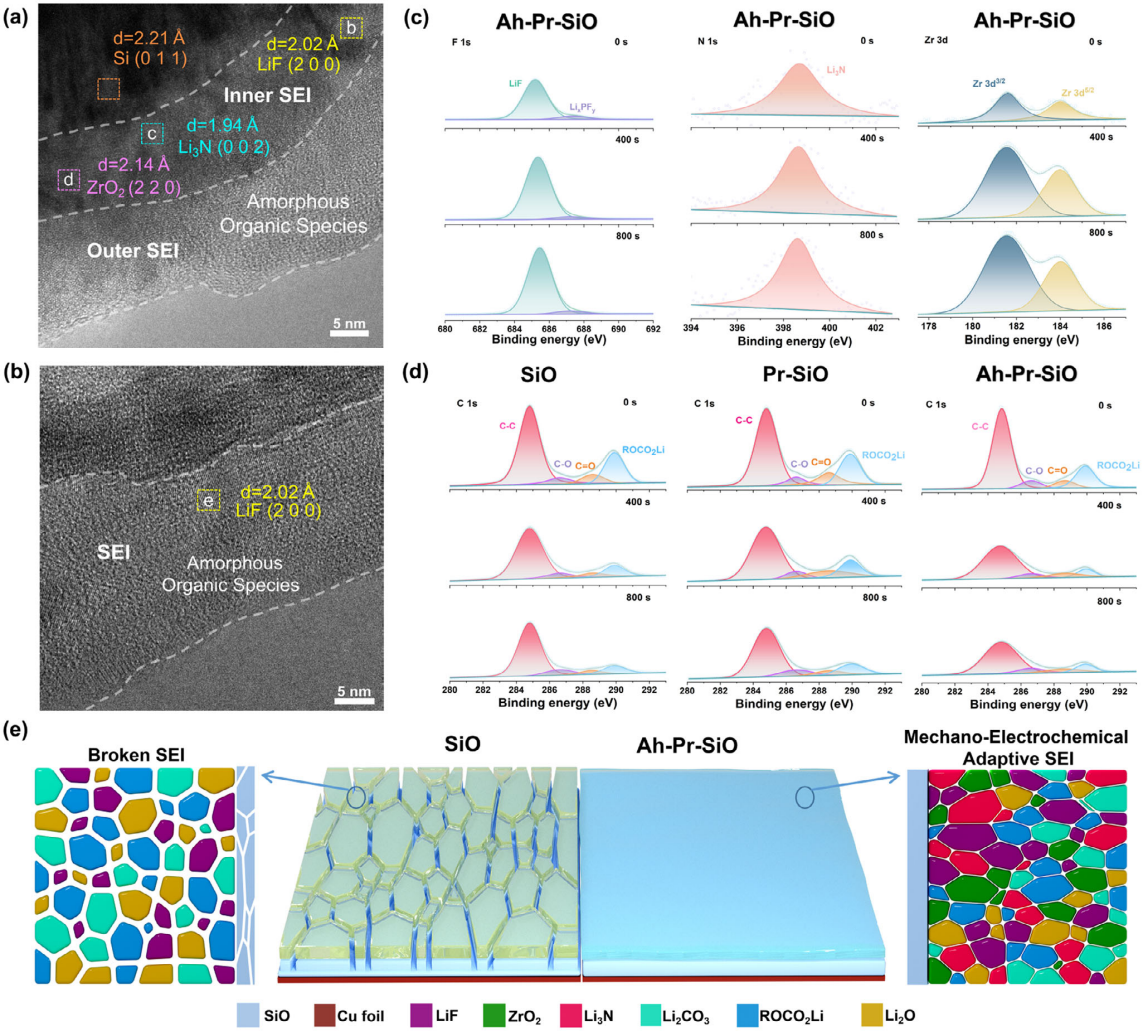
HR‐TEM images of the a) Ah‐Pr‐SiO and b) SiO after cycling. c) The F 1s, N 1S, and Zr 3d XPS spectra depth profiles of the Ah‐Pr‐SiO. d) The C 1s XPS spectra depth profiles of SiO, Pr‐SiO, and Ah‐Pr‐SiO after three cycles, respectively. e) Schematic of the SEI on the SiO and Ah‐Pr‐SiO anode, respectively.

To explore the favorable mechanical and electrochemical performance of the Ah‐Pr‐SiO anode, SEM and HR‐TEM images of all electrodes were analyzed. Due to the inability of the SEI to withstand the large volume expansion, visible cracks appeared on the surface of SiO after 200 cycles (Figure S32, Supporting Information). In contrast, the surface of Ah‐Pr‐SiO is almost free of obvious cracks. In addition, the cross‐sectional SEM images show that after 200 cycles, the volume expansion of Ah‐Pr‐SiO is lower at 35.7% (25.29 µm vs 34.33 µm) than that of SiO at 64.7% (26.97 µm vs 44.43 µm) (Figure S33, Supporting Information). Owing to the mechano‐electrochemical adaptive SEI on the surface of Ah‐Pr‐SiO, which enhances the mechanical stability to accommodate large volume expansion, leading to better cycle stability. HR‐TEM analyses reveal distinct nanostructures and main compositions of SEI layers between Ah‐Pr‐SiO and SiO. In particular, the distinct bilayered structure of SEI can be observed in the Ah‐Pr‐SiO, rather than a single‐layered SEI structure of SiO (**Figure** **6**a,b). Ah‐Pr‐SiO and SiO used for HR‐TEM testing underwent 30 cycles, at which point a stable SEI had formed. Noted that the inner layer of the bilayer SEI consists of a variety of inorganic components, while the outer layer consists mainly of amorphous organic ingredients, such as ROCO_2_Li, etc. The main components of the inner layer are LiF ((2 0 0)‐facet lattice spacing, 2.02Å), Li_3_N ((0 0 2)‐facet lattice spacing, 1.94 Å) and ZrO_2_ ((2 2 0)‐facet lattice spacing, 2.18 Å) (Figure S34a–d; Figure S35, Supporting Information). In contrast, the conventional SEI on the surface of SiO consists of LiF ((2 0 0)‐facet lattice spacing, 2.02Å), which is dispersed in organic amorphous species (Figure S34e, Supporting Information). The SEM and HR‐TEM results show that the mechano‐electrochemical adaptive SEI with a unique bilayer structure can effectively adapt to the volume expansion of SiO during cycling and has excellent structural stability.

Ar^+^ sputtering XPS technology was employed to further investigate the specific composition and distribution of the mechano‐electrochemical adaptive SEI during the cycling process (Figure 6c,d). Before etching, the C1s spectra of all three samples show main characteristic peaks of C═O, C─O, and C─C, which correspond to decomposition products in the organic electrolyte solution. The lower organic components with increasing etch depth were detected in Ah‐Pr‐SiO compared with SiO and Pr‐SiO, and this contrast is more pronounced in the inner layer of the SEI layer. In addition, inorganic components (LiF, Li_3_N, and ZrO_2_ signal in F 1s, N 1s, and Zr 3d spectrum, respectively) are uniformly distributed in the SEI layer of Ah‐Pr‐SiO compared to SiO and Pr‐SiO, and increased with etching depth (Figure S36, Supporting Information). The atomic percentages of the SEI signals from SiO, Pr‐SiO, and Ah‐Pr‐SiO are shown in Figure S37 and Table S4 (Supporting Information). Ar^+^ sputtering XPS results again demonstrate that the inner layer of the SEI layer of Ah‐Pr‐SiO after cycles is primarily composed of inorganic components such as LiF, Li_3_N, and ZrO_2_, while the outer layer mainly consists of organic components like alkyl lithium (Figure 6e). This is attributed to the pre‐construction of highly stable and dense inorganic‐rich SEI after a spontaneous chemical reaction, which effectively suppresses the decomposition of the organic electrolyte on the surface of SiO. The pre‐constructed mechano‐electrochemical adaptive interphase can effectively modulate the formation of the SEI layer, including inorganic components and spatial configurations, to provide impressive mechanical and electrochemical performance for Ah‐Pr‐SiO.

The enhancement of mechanical and electrochemical properties proves the superiority of mechano‐electrochemical adaptive SEI, which exhibits an inner rigid and outer flexible bilayer structure (Figure 6e). On the one hand, the LiF and ZrO_2_ components in the inner layer provide high strength and modulus for SEI due to their superior mechanical properties, together with the organic components in the outer layer, enhance the toughness of the SEI layer. The combination of strength and toughness of SEI is sufficient to accommodate volume expansion of the SiO and mitigate irreversible degradation of electrode capacity due to electrolyte decomposition. On the other hand, a variety of inorganic components, especially Li_3_N, make the SEI layer with a low Li^+^ diffusion barrier, which facilitates a more rapid transport of Li^+^ across the SEI for superior Li^+^ diffusion kinetics.

**Figure 7:**
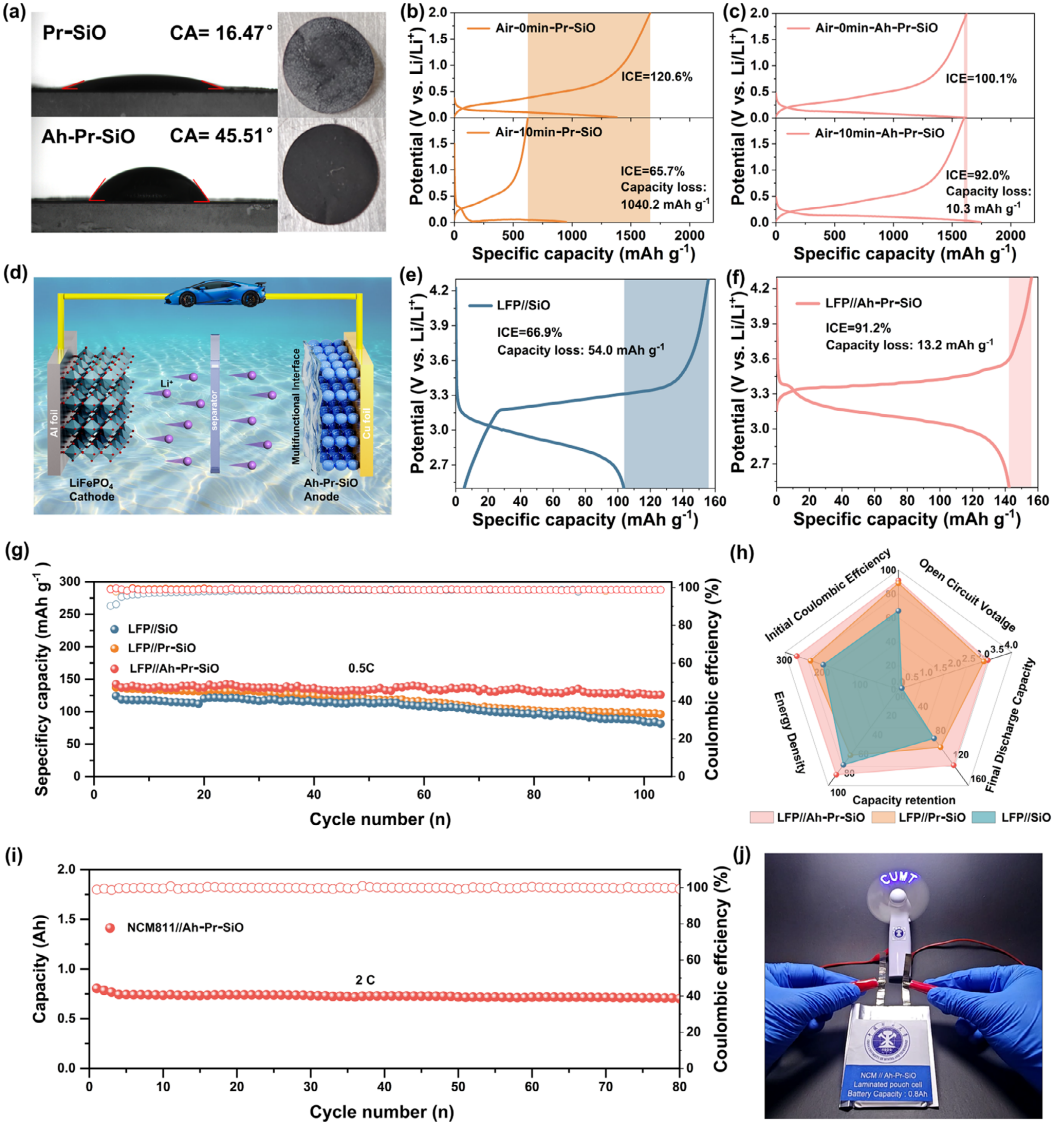
a) The contact angle with water and the digital images resting in the air for 10 min of the Pr‐SiO and Ah‐Pr‐SiO electrodes. The first charge/discharge curves of b) Pr‐SiO and c) Ah‐Pr‐SiO resting in air for 0 and 10 min. d) Schematic diagram of LFP//Ah‐Pr‐SiO. The first charge/discharge curves of e) LFP//SiO and f) LFP//Ah‐Pr‐SiO. g) The cycling performance and h) radargram of LFP//Ah‐Pr‐SiO, LFP//Pr‐SiO and LFP//SiO, respectively. i) The cycling performance of NCM811//Ah‐Pr‐SiO pouch cell. j) NCM811//Ah‐Pr‐SiO pouch cell lights up the LED bulb and powers the small electric fan.

Notably, improving the air stability of prelithiated electrode is the key to achieve the industrial compatibility of chemical prelithiation technology.^[^ ^41^ ^]^ For the Pr‐SiO, due to the early replenishment of active lithium, the SiO particles will form a large number of lithium‐silicon alloys and other active substances, which have a high chemical reactivity to moisture and oxygen.^[^ ^42^ ^]^ After standing in the air, the surface of Pr‐SiO will soon be eroded by moisture and oxygen, and inert lithium salt will form new phases such as Li_2_O, which could directly affect the ICE and capacity. To verify the change in air stability of three samples, the contact angle with deionized water was tested. When in contact with a water solution, the contact angle of Ah‐Pr‐SiO (45.51°) is smaller than Pr‐SiO (16.47°) and SiO (54.41°), demonstrating the hydrophobicity of Ah‐Pr‐SiO is significantly enhanced after inorganic‐rich interphase is constructed (**Figure** **7**a; Figure S38, Supporting Information). Optical photographs of Pr‐SiO and Ah‐Pr‐SiO after 10 min of exposure to air (25 °C temperature, 70 % humidity) show a large amount of white by‐products on the surface of the former and no change in the latter (Figure 7a), indicating a significant decrease in the reactivity of Ah‐Pr‐SiO toward moisture and oxygen. Moreover, the ICE and specific capacity of Pr‐SiO and Ah‐Pr‐SiO after 10 min of exposure to air were further investigated (Figure 7b,c). The ICE of Pr‐SiO was reduced from 120.6% to 65.7% with a capacity decay of 1040.2 mAh g^−1^, as comparison, Ah‐Pr‐SiO remains a high ICE of ≈92.0% with a capacity almost unchanged. The enhanced air stability is attributed to the homogeneous and dense inorganic‐rich interphase, as well as the high stability conferred by the LiF and ZrO_2_, which effectively insulate them from water and oxygen attacks.

To elucidate the practical application potential of Ah‐Pr‐SiO as an anode for LIBs, the full cells (LFP//Ah‐Pr‐SiO, LFP//Pr‐SiO, LFP//SiO) were assembled using LiFePO_4_ (LFP) as the cathode, together with Ah‐Pr‐SiO, Pr‐SiO, and SiO as the anode, respectively (Figure 7d). The LFP//Ah‐Pr‐SiO delivers a high ICE of 91.2%, with a capacity loss of only 13.2 mAh g^−1^, which is much better than LFP//SiO (ICE 66.9%, capacity loss of 54 mAh g^−1^) (Figure 7e,f). After 100 cycles at 0.5 C, the LFP//Ah‐Pr‐SiO remains a high specific capacity of 126.1 mAh g^−1^ (capacity retention of 88.6%), which is higher than the LFP//SiO with 81.4 mAh g^−1^ and LFP//Pr‐SiO with 96.2 mAh g^−1^ (Figure 7g; Figure S39, Supporting Information). It is worth noting that although chemical prelithiation effectively improved the ICE and reversible capacity of LFP//Pr‐SiO, the capacity decay was relatively fast due to the severe volume expansion of the Pr‐SiO anode (Figure S40, Supporting Information). The radargram reveals the detailed parameters of LFP//SiO, LFP//Pr‐SiO and LFP//Ah‐Pr‐SiO, respectively (Figure 7h). The LFP//Ah‐Pr‐SiO exhibits a high energy density of 268.6 Wh kg^−1^ (based on Equation S5, Supporting Information), which is higher than LFP//SiO (198.8 Wh kg^−1^), this demonstrates the enormous potential of chemical prelithiation‐mediated SEI pre‐construction strategy in silicon‐based anode materials for LIBs. To further illustrate the practical application of the Ah‐Pr‐SiO anode for LIBs with high energy density, an Ah‐level pouch cell was assembled with LiNi_0.8_Co_0.1_Mn_0.1_O_2_ (NCM811//Ah‐Pr‐SiO) (Figure S41 and Table S5, Supporting Information). As expected, the battery demonstrated outstanding electrochemical performance, achieving a reversible capacity of 0.8 Ah at a 0.2C charging rate and a high energy density of 346.6 Wh kg^−1^ based on the total battery mass, which is one of the best reported values for SiO‐based lithium‐ion full cells (Table S6, Supporting Information). Remarkably, a high capacity retention of 87.1% was maintained after 80 charge–discharge cycles at a 2C charging rate. (Figure 7i; Figure S42, Supporting Information). Additionally, as shown in Figure 7j, the NCM811//Ah‐Pr‐SiO pouch cell can power a small electric fan and light an LED bulb, verifying the practical application value of the SiO anode with a mechano‐electrochemical adaptive SEI (Video S1, Supporting Information).

## 3 Conclusion

In summary, we propose a chemical prelithiation‐mediated in situ pre‐constructed SEI strategy by the spontaneous reaction of Ah with the Pr‐SiO, which enhances the ICE meanwhile improving Li^+^ diffusion kinetics and structural stability of the chemically prelithiated SiO anode. Notably, the pre‐constructed SEI is rich in LiF, ZrO_2_, and Li_3_N components, in which the LiF and ZrO_2_ provide favourable mechanical properties and interface stability, and Li_3_N provides high Li^+^ flux. The mechano‐electrochemical adaptive SEI possesses characteristics of inner rigidity and outer flexibility, providing exceptional cycling stability and Li^+^ diffusion kinetics for the SiO anode. Moreover, the pre‐constrcuted inorganic‐rich interphase can adapt to the stress changes occurring during the prelithiation process, together with also providing superior air stability. The Ah‐Pr‐SiO half cells exhibit considerably improved rate and cycle performance, while maintaining higher ICE compared to SiO and Pr‐SiO. The assembled lithium‐ion full cells (LFP//Ah‐Pr‐SiO) deliver a high ICE (91.2%), and the assembled pouch cell (NCM811//Ah‐Pr‐SiO) exhibits stable cycling with a high energy density (346.6 Wh kg^−1^). This work provides an advanced prelithiation‐mediated pre‐constructed SEI strategy, offering the possibility for high energy density Si‐based anodes in lithium‐ion batteries with long cycle life.

## Conflict of Interest

The authors declare no conflict of interest.

## Supporting information



Supporting Information

Supporting Information

## Data Availability

The data that support the findings of this study are available from the corresponding author upon reasonable request.
