# Supplementary material for: Pre‐Constructed Mechano‐Electrochemical Adaptive Solid Electrolyte Interphase to Enhance Li+ Diffusion Kinetics and Interface Stability for Chemically Prelithiated SiO Anodes
Source: Adv Sci (Weinh). 2025 Oct 16;12(48):e15555. doi: 10.1002/advs.202515555 (PMC12752609; doi:10.1002/advs.202515555)
Supplement: Supplementary file 1 — Supporting Information [file ADVS-12-e15555-s001.docx]

Supporting Information

**Pre-constructed Mechano-Electrochemical Adaptive Solid Electrolyte Interphase to Enhance Li^+^ Diffusion Kinetics and Interface Stability for Chemically Prelithiated SiO Anodes**

*Zhan Wang, Shuang Li, Yun Zheng, Yinan Liu, Qilin Feng, Chencheng Xu, Quanchao Zhuang, Zhicheng Ju, Jiangmin Jiang*, Huaiyu Shao*, Xiaogang Zhang**

**Experimental Procedures**

**Materials and chemicals**

Biphenyl (BP, 99%), 2-methyltetrahydrofuran (2-Me-THF, 99%), Dimethyl sulfoxide (DMSO, 99.9%) and Ammonium hexafluorozirconate ((NH_4_)_2_ZrF_6_) were purchased from Aladdin. The pristine SiO, LiFePO_4_ and LiNi_0.8_Co_0.1_Mn_0.1_O_2_ raw material were supplied by Contemporary Amperex Technology Co., Limited. All other chemicals were of analytical grade and used as received.

**Preparation of electrodes**

The anode with copper foil as the current collector is composed of 70 % SiO, 20% Super P conductive additive, and 10% polyacrylic acid Lithium (PAA-Li) binder. The slurry-coated electrode was initially dried under a conditions at 60 °C for 12 hours in a convection. Following this, the electrode was fabricated into 12-mm-diameter circular geometries and the mass loading of about 1.5 mg/cm^2^, subjecting it to vacuum drying at 80 °C for 8 hours before cell assembly. The type of separator used was Celgard 2500.

**Preparation of Pr-SiO electrodes**

The chemical prelithiation reagent was prepared by dispersing a certain amount of BP in 2-Me-THF solvent, completely dissolved by magnetic stirring (800 rmp) and then adding an excess of lithium metal (n_Li_:n_BP_= 4:1) to obtain a solution with a concentration of 0.5 M. Subsequently stirred for 1h to obtain a Li-BP-2Me-THF solution with a dark green color. The Pr-SiO were prepared by immersing the SiO anode in the Li-BP-2Me-THF solution for varying degrees of lithiation. The above operations were carried out in an Ar atmosphere (ppm<0.1).

**Preparation of Ah-Pr-SiO electrodes**

The (NH_4_)_2_ZrF_6_ powder was added in DMSO under agitated stirring for 12 h to obtain the 0.02 M precursor (NH_4_)_2_ZrF_6_/DMSO solution. The Ah-Pr-SiO was prepared by immersing the Pr-SiO anode in the (NH_4_)_2_ZrF_6_/DMSO solution for varying degrees of in situ reaction. The electrodes were then cleaned with corresponding ether solvents and dried at 30 °C. The above operations were carried out in an Ar atmosphere (ppm<0.1).

**Structural characterizations**

The morphological information of the electrode was carried out using a field emission scanning electron microscope (FSEM) with a MAIA3 LMH and high-resolution transmission electron microscopy (HRTEM, Tecnao G2 F20). X-ray photoelectron spectra (XPS) were recorded using a Perkin-Elmer PHI 5400 ESCA system. Atomic force microscope (AFM) measurements were carried out using a Bruker Dimension ICON. To ensure accurate experimental results, all tests should be made to ensure as much protection as possible against oxygen attack.

**Electrochemical Characterizations**

The R2032 coin cells were assembled in an Ar atmosphere, with the electrolyte comprising 1 M LiPF_6_ in EC/DEC (v: v = 1:1) and 5%FEC. Tests were conducted using a NEWARE system to charge/discharge at a constant temperature of 25 °C, with the half-cell voltage range set from 0.01 to 2.0 V and the full-battery voltage range set from 2.5 to 4.2 V. GITT measurements of the batteries were conducted with a 0.2 C current pulse duration after 3 cycles. Cyclic voltammetry (CV) measurements were conducted using a sweep rate of 0.01 mV s^-1^ on a VSP-300 Electrochemical Workstation. Electrochemical impedance spectroscopy (EIS) measurements were carried out over a frequency range from 0.01 Hz to 100 kHz. Variable-temperature EIS test is performed in a variable temperature oven.

**Preparation for Lithium-ion Full Batteries**

The anode with copper foil as the current collector is composed of 80% LiFePO_4_ (LFP), 10% Super P, and 10% [polyvinylidene](https://baike.baidu.com/item/polyvinylidene/53069568?fromModule=lemma_inlink) fluoride (PVDF) binder. The slurry-coated electrode was initially dried under a conditions at 60 °C for 12 hours in a convection. Following this, the electrode was fabricated into 12-mm-diameter circular geometries, subjected to vacuum drying at 80 °C for 8 hours prior to cell assembly. The lithium-ion full batteries were assembled using commercialized LFP cathode and SiO, Pr-SiO and Ah-Pr-SiO anodes. The capacity ratio of anode to cathode (N/P) was about 1.1:1. The type of separator used was Celgard 2500. For the mass loading of the LiFePO_4_ cathode, to ensure that the N/P ratio of the full cell was 1.1, the mass load of the SiO cathode used was 0.7 mg/cm^2^, and the mass load of the LFP cathode was 5 mg/cm^2^. For pouch cell, The LiNi_0.8_Co_0.1_Mn_0.1_O_2_ (NCM811) anode with copper foil as the current collector is composed of 95% NCM811, 2.5% Super P and 2.5% PVDF binder. The N/P ratio was about 1.1:1.

**Theoretical calculations**

The impedance at full point was simulated and analyzed using the Matrix Laboratory （MATLAB R2023b） software package. Molecular dynamic (MD) simulations were performed with Forcite module in Material Studio 2023 software with respect to the Li-BP-2Me-THF prelithiation reagent. The molecular structures of 2Me-THF and BP were modelled and optimized using the clean function of the Visualizer module. To optimize the geometry in the DMol3 quantum mechanical calculation module, select the Geometry Optimization task, select Fine Calculation Precision, select the Hybrid-B3LYP general function, tick Spin unrestricted, use All Electron to handle the core electrons, adopt the DNP basis group and other setup parameters. The three molecular structures obtained in the first step were subjected to geometry optimization calculations using the default software values to obtain their optimal structures. Next, the DMol3 quantum mechanical calculation module was used to perform a single-point energy calculation task on the optimal molecular structure obtained in the previous step. To begin, launch the Analysis function in the DMol3 module. Then, perform Population and Potentials analyses to obtain electronic property data, such as HOMO and LUMO energy levels and ESP.

**Distribution of relaxation times (DRT) fitting**

In the event that the voltage response of an electrochemical system to a step current perturbation is assumed to be exponentially decaying with a specific time scale distribution, the impedance of the electrochemical system can be described by equation below.

$$Z(\omega)=R_{0}+Z_{pol}(\omega)=R_{0}+R_{pol}\int_{0}^{\infty} \frac{g(\tau)}{1+j\omega\tau}d\tau$$

$$\int_{0}^{\infty} g(\tau)\text{ }d\tau=0$$

$R_{0}$represents the ohmic resistance of the electrochemical system, $Z_{pol}(\omega)$is the polarisation impedance,$R_{pol}$is the polarisation resistance, $\tau$is the relaxation characteristic time, $g(\tau)$ is the distribution function of the relaxation characteristic time, $j$is the complex unit, and ω is the angular frequency^[1]^.

**Supporting Figures**

**
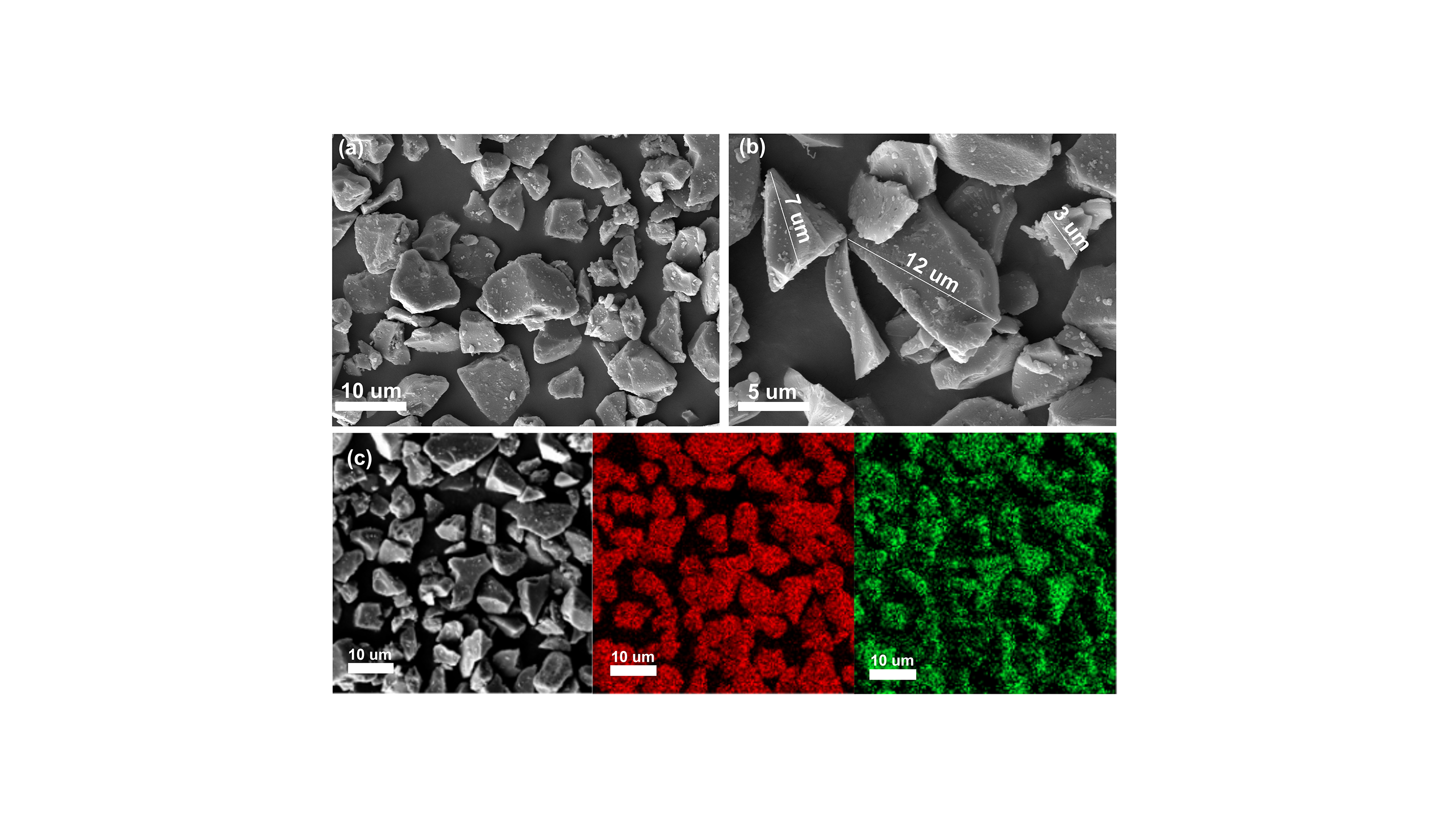
**

**Figure S1.** SEM images and EDS mapping of SiO.


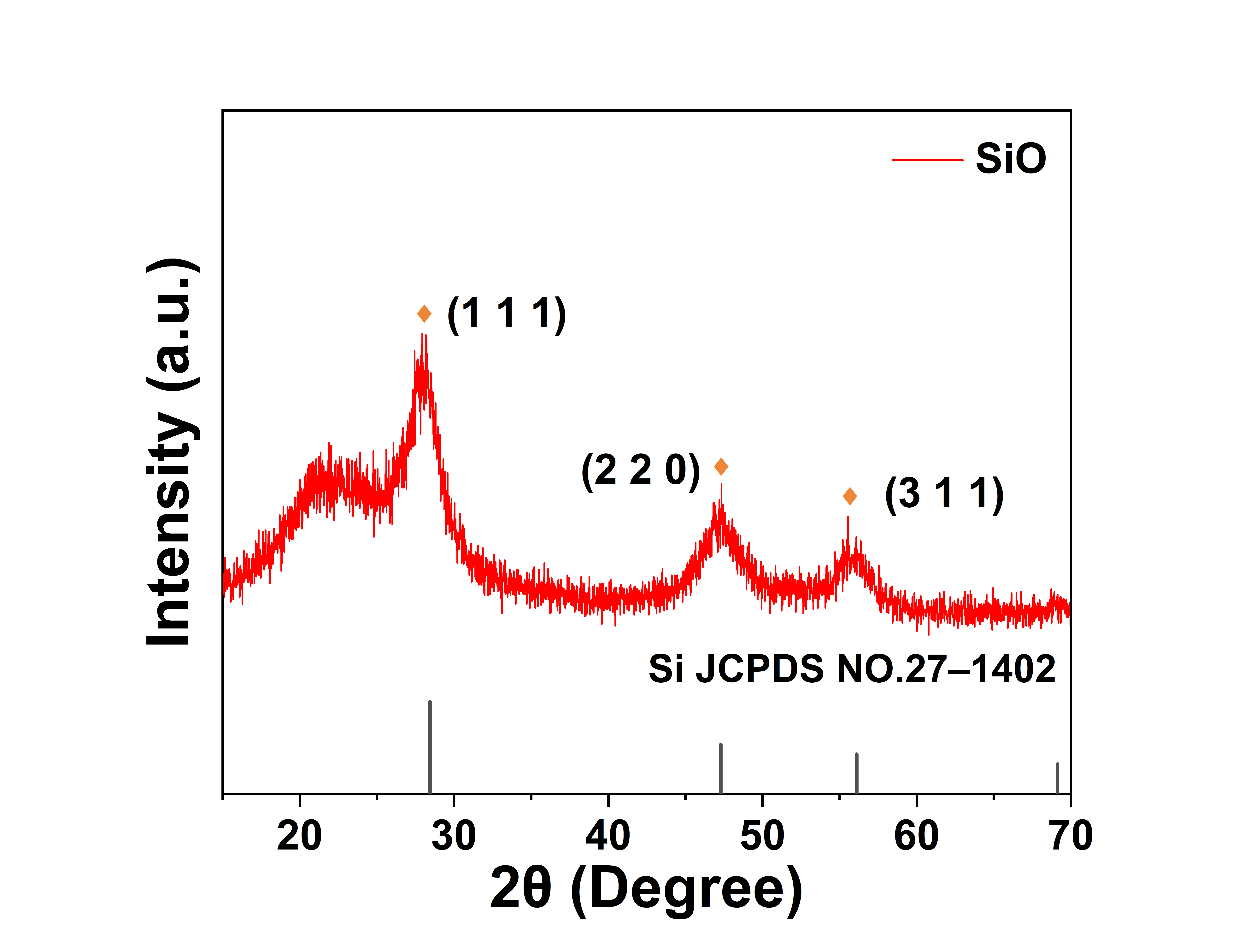


**Figure S2.** XRD pattern of SiO.

**
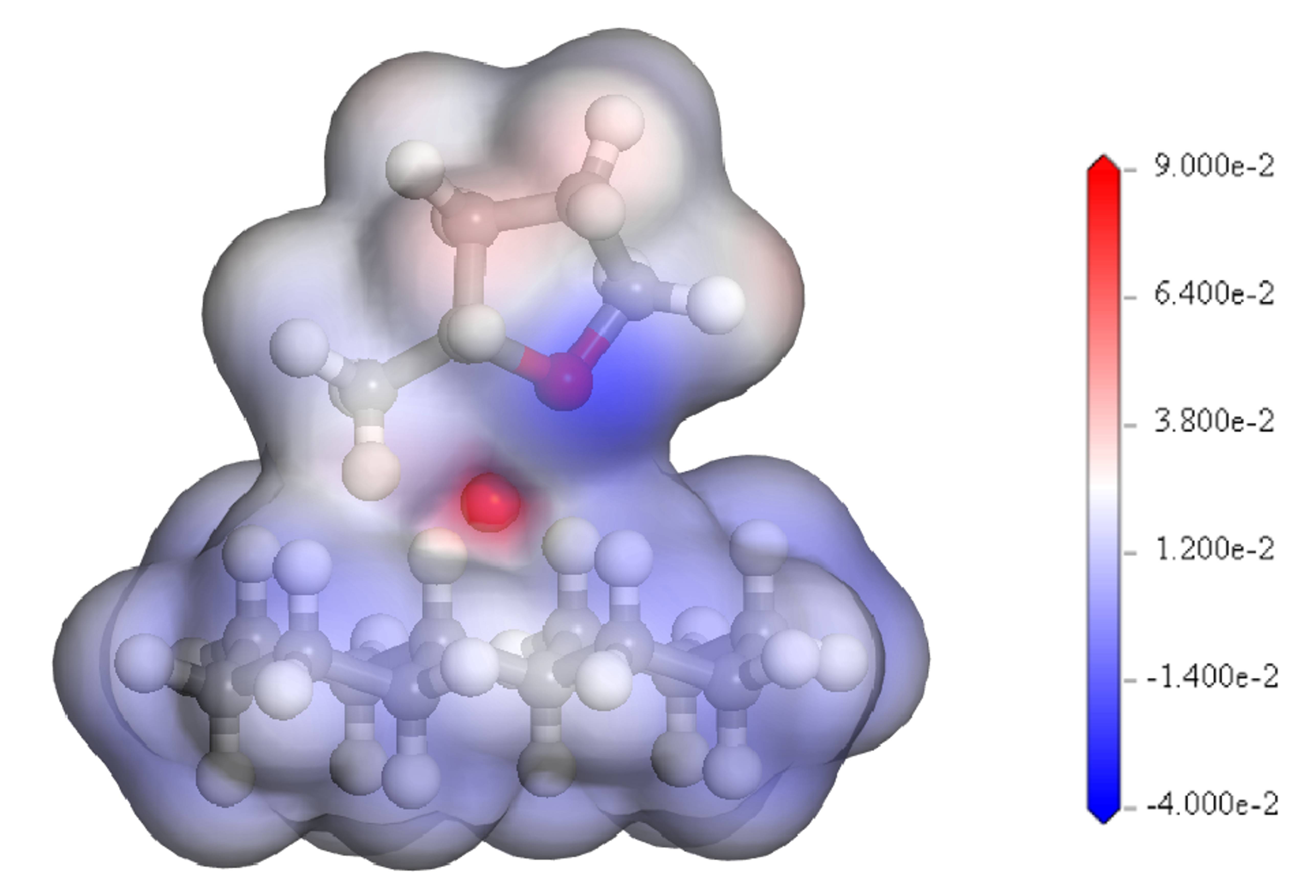
**

**Figure S3.** Electrostatic potential mapping of Li-BP/2-Me-THF.


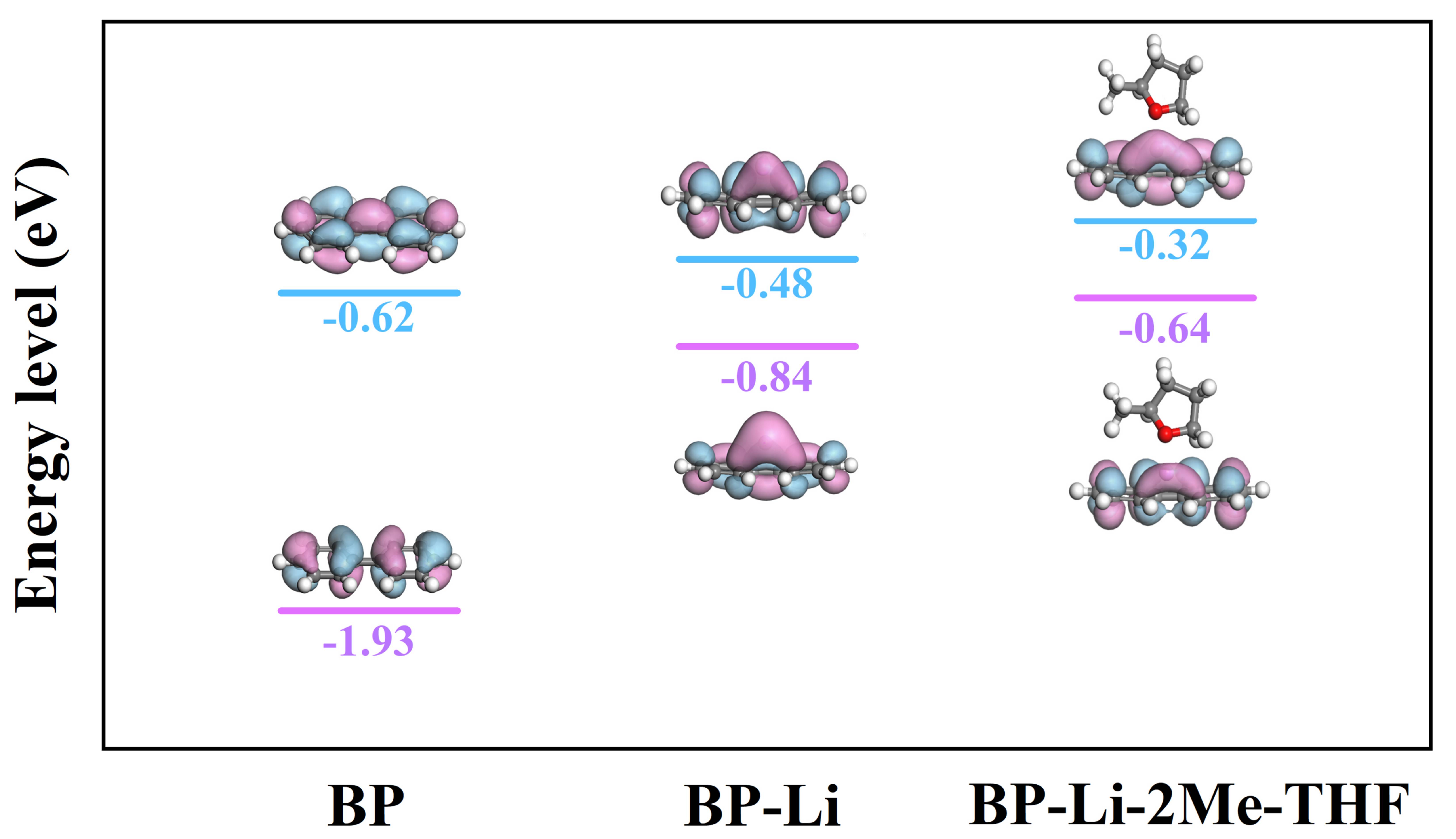


**Figure S4.** Geometrical configurations and LUMO/HOMO energy levels of the Li-BP/2-Me-THF solvent.


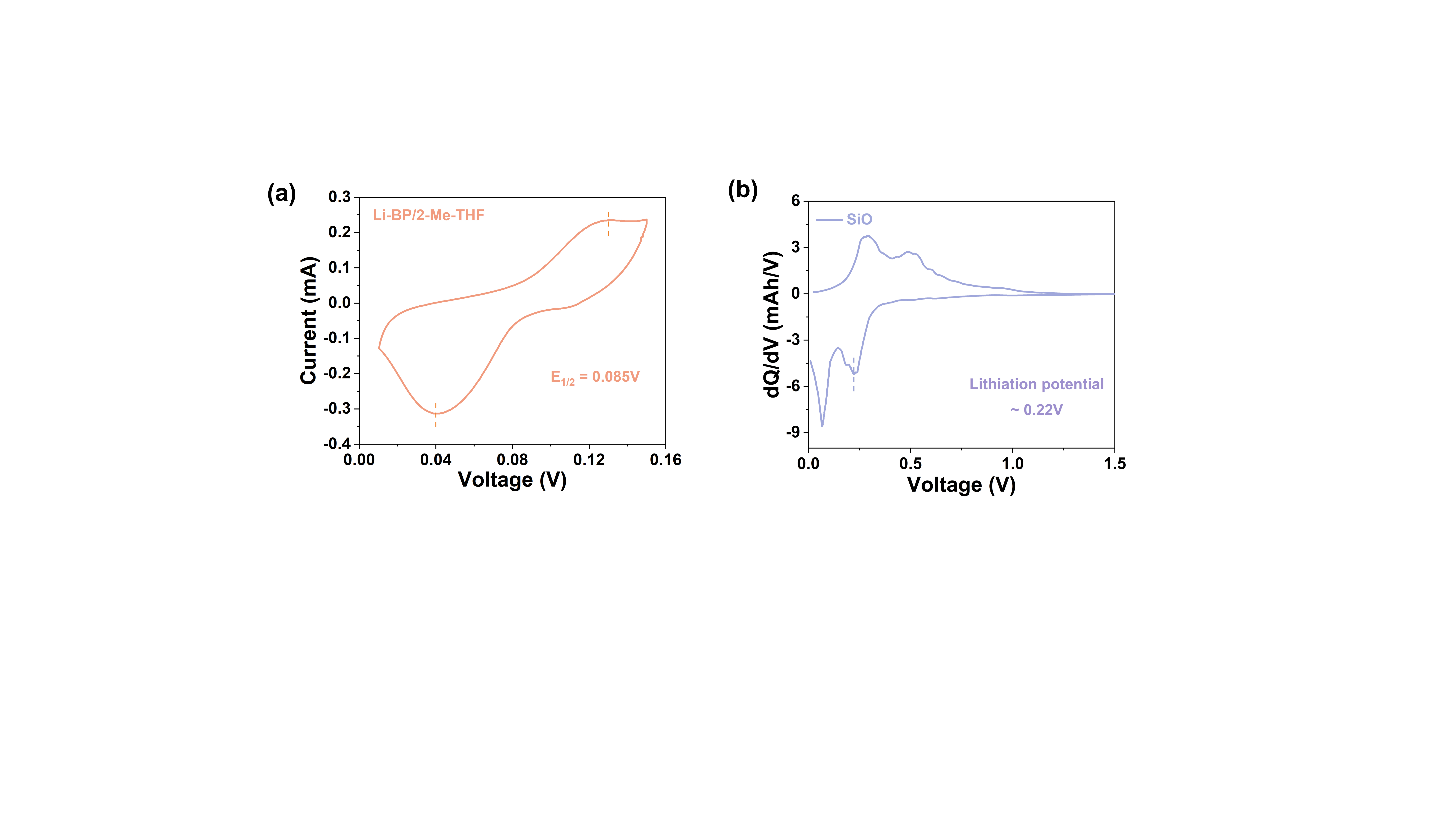


**Figure S5.** CV curve of Li-BP/2-Me-THF solvent and dQ/dV curve of SiO.


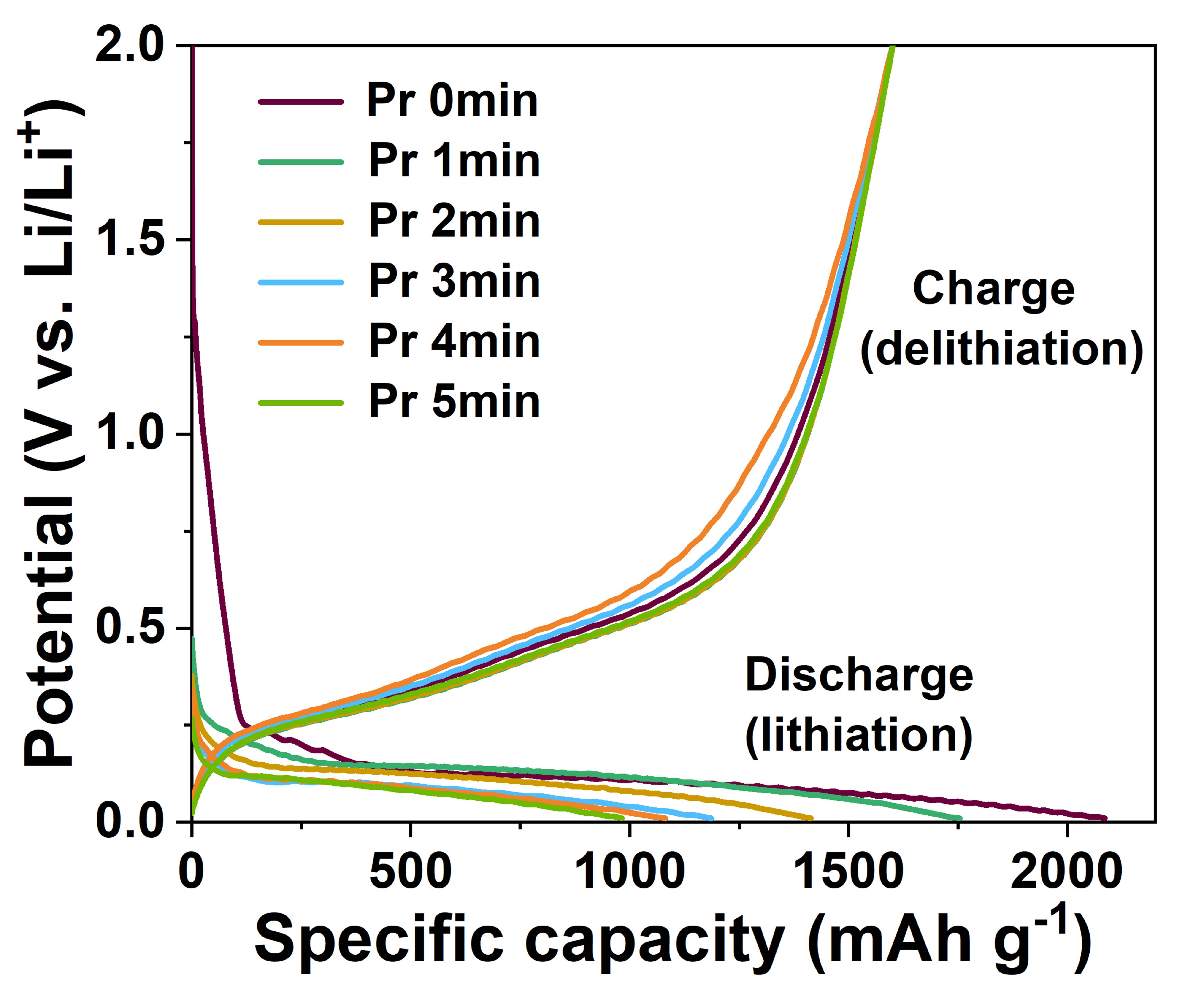


**Figure S6.** Initial charge/discharge curves of Pr-SiO at various prelithiation times.

**
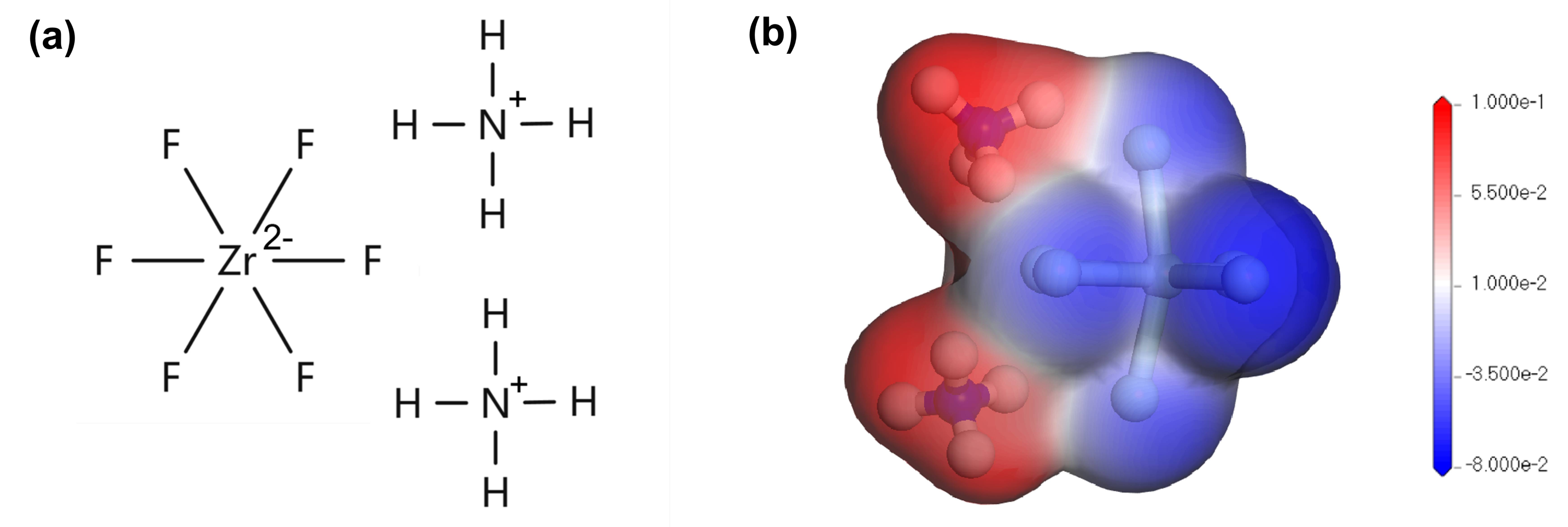
**

**Figure S7.** (a) Molecular structural formula and (b) electrostatic potential mapping of Ah.


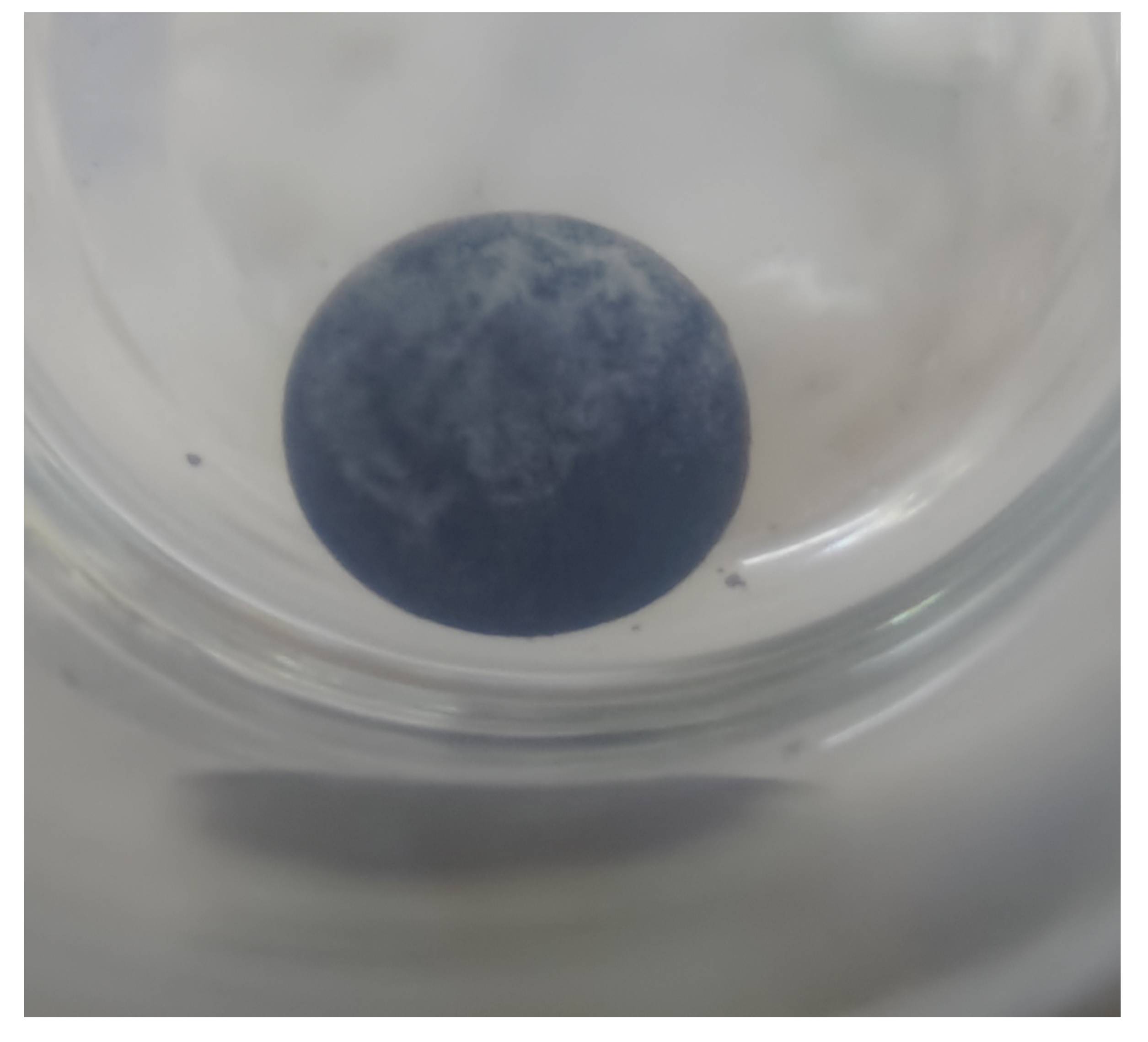


**Figure S8.** Optical image of the interface modification process.


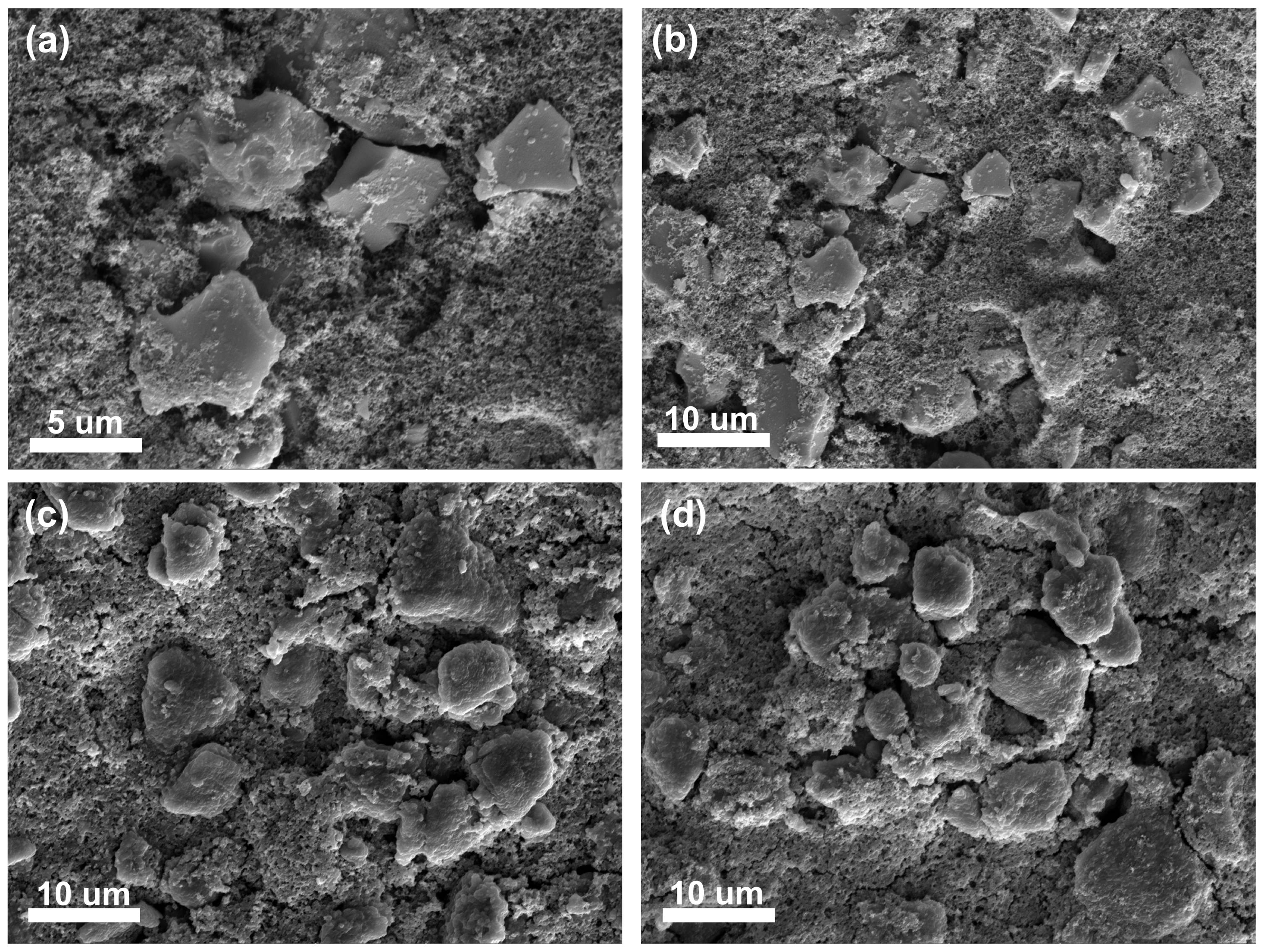


**Figure S9.** SEM images of (a) and (b) SiO, (c) Pr-SiO, and (d) Ah-Pr-SiO, respectively.


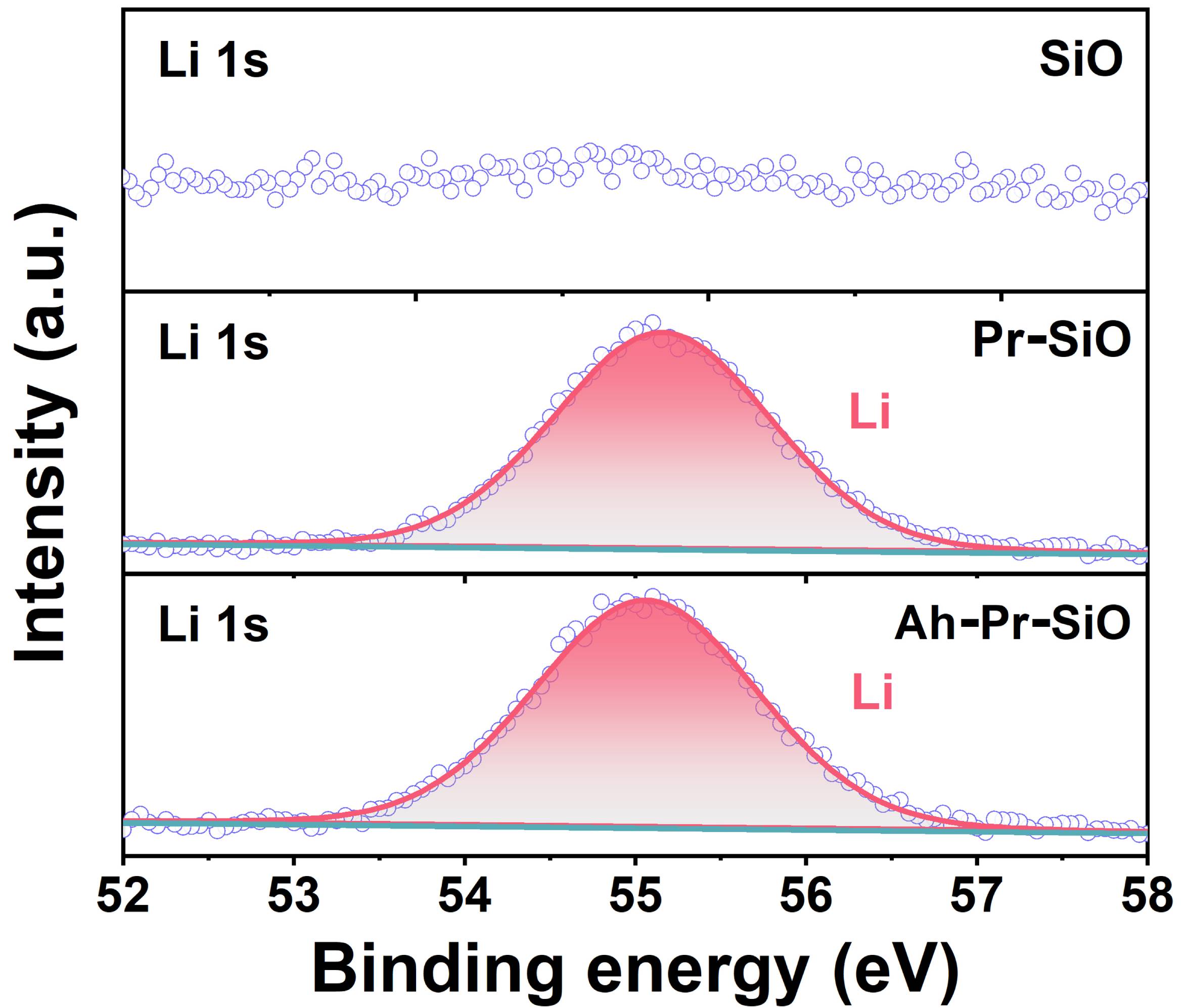


**Figure S10.** XPS spectra of Li 1s.


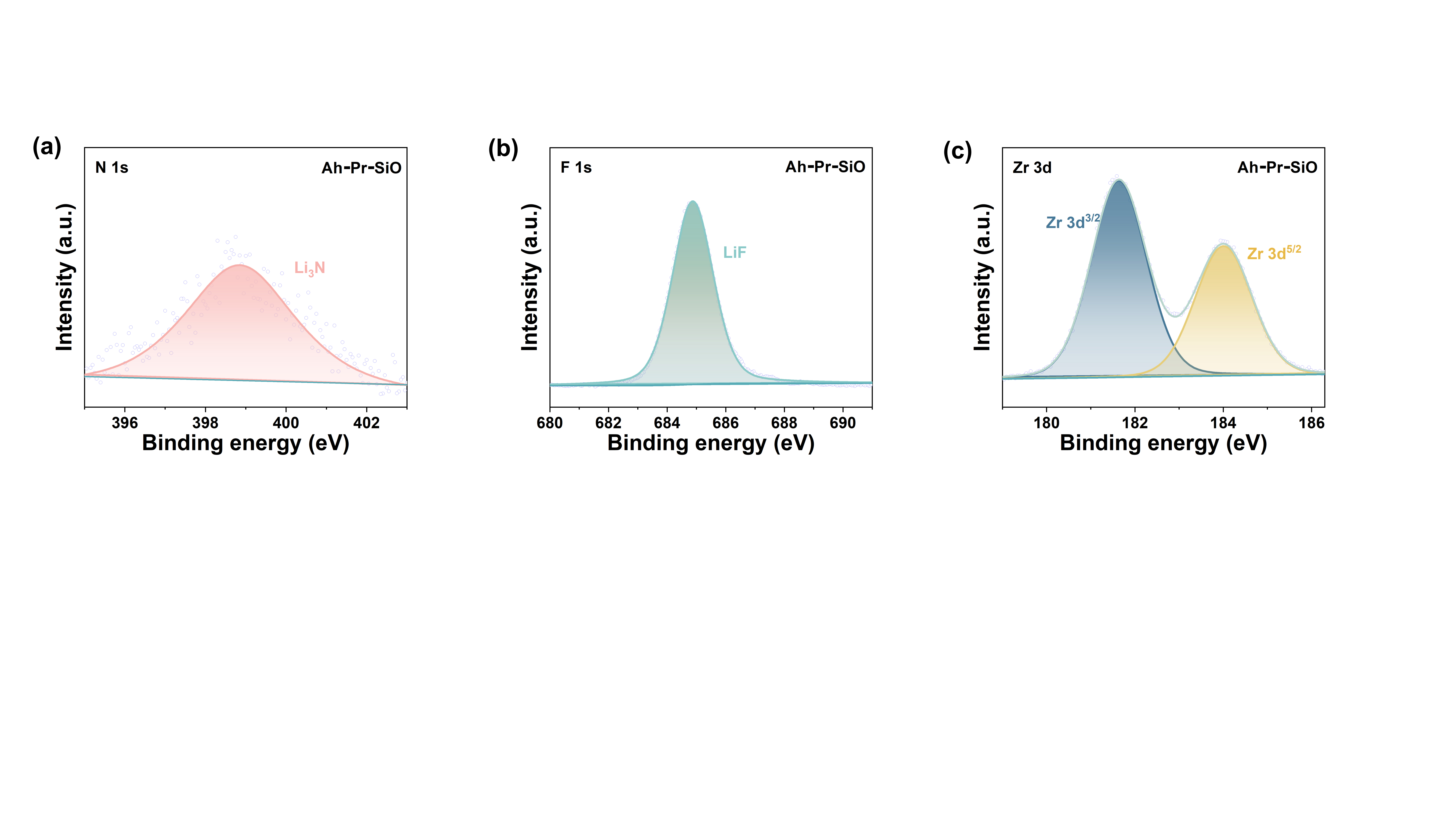


**Figure S11.** XPS spectra of (a) N 1s, (b) F 1s and (c) Zr 3d, respectively.


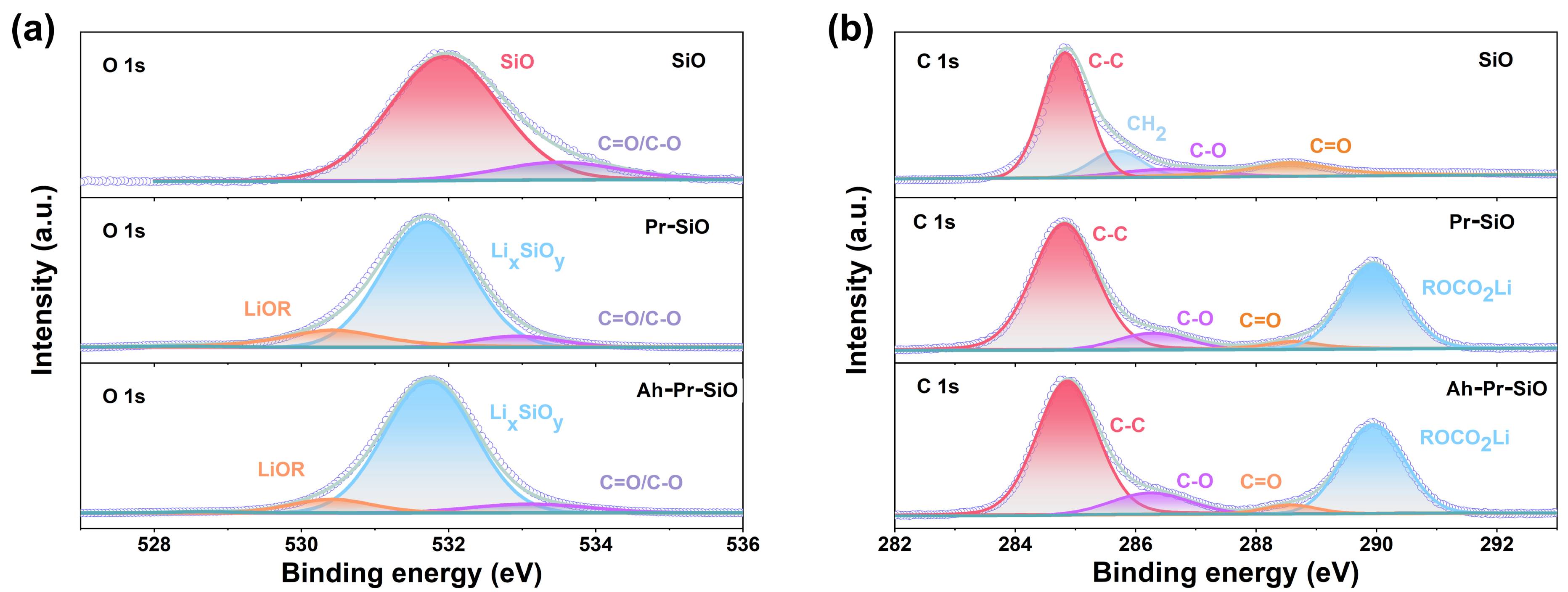


**Figure S12.** XPS spectra of (a) O 1s and (b) C 1s, respectively.


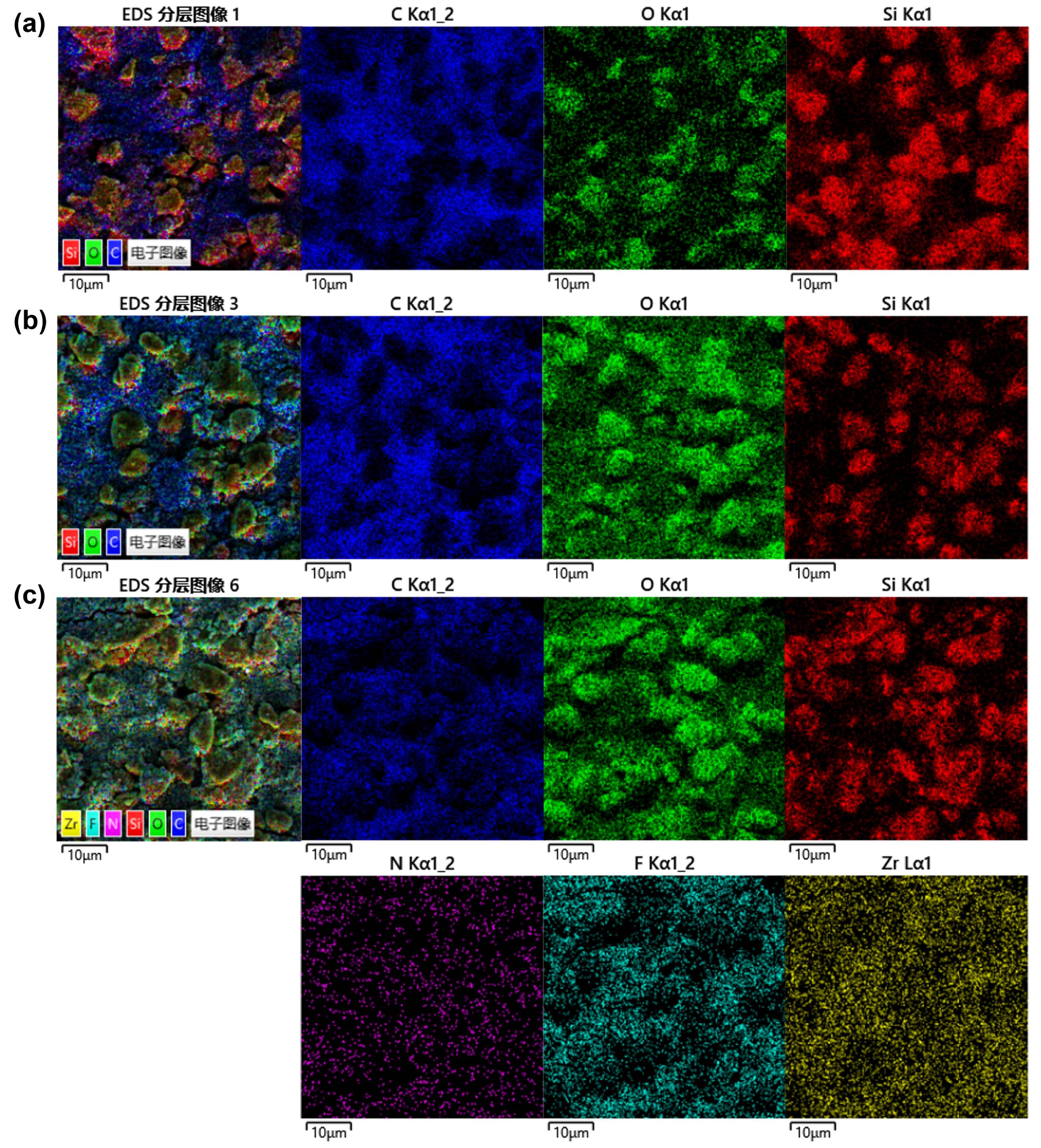


**Figure S13.** SEM**-**EDS mapping of (a) SiO, (b) Pr-SiO, and (c) Ah-Pr-SiO, respectively.


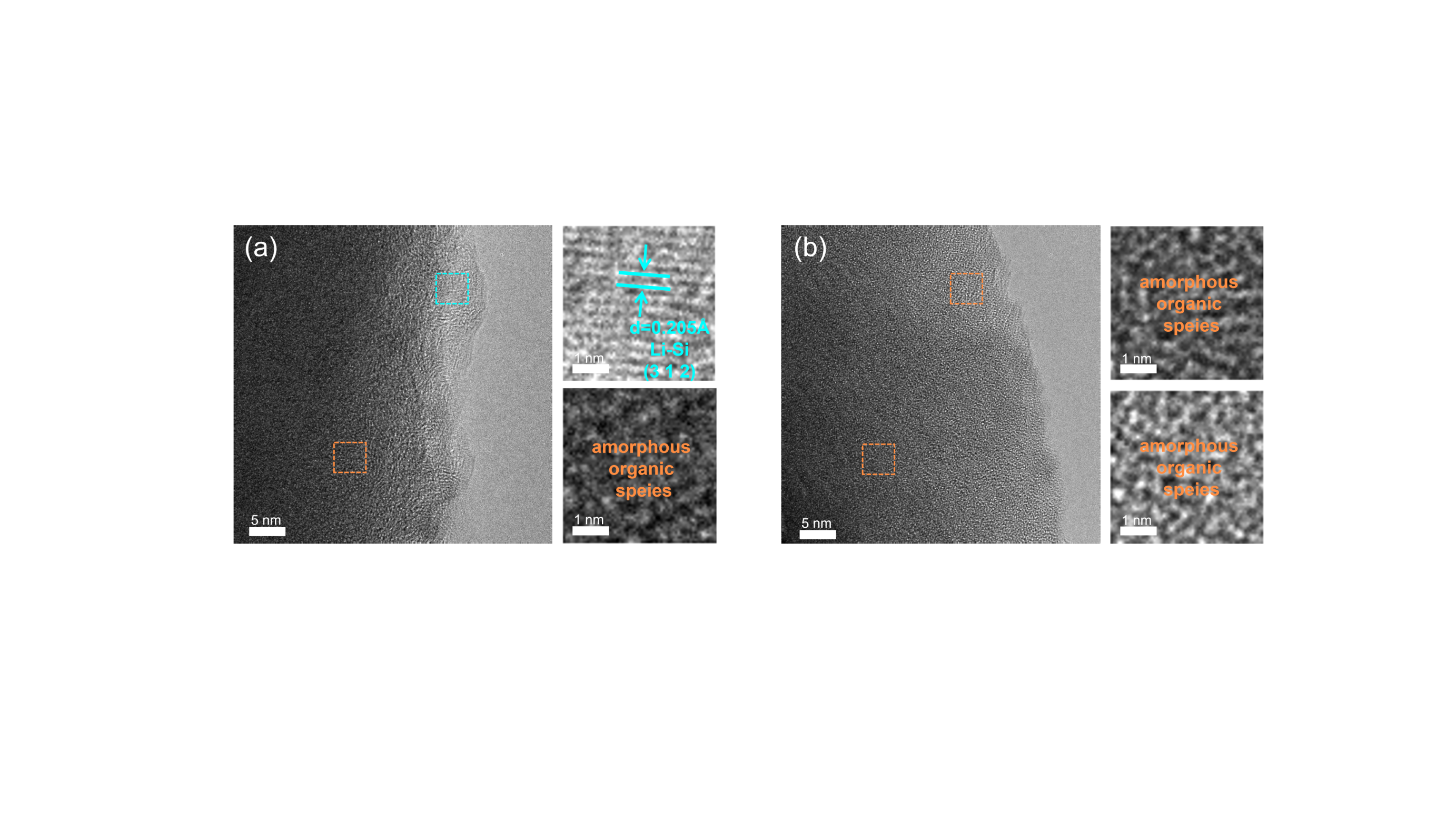


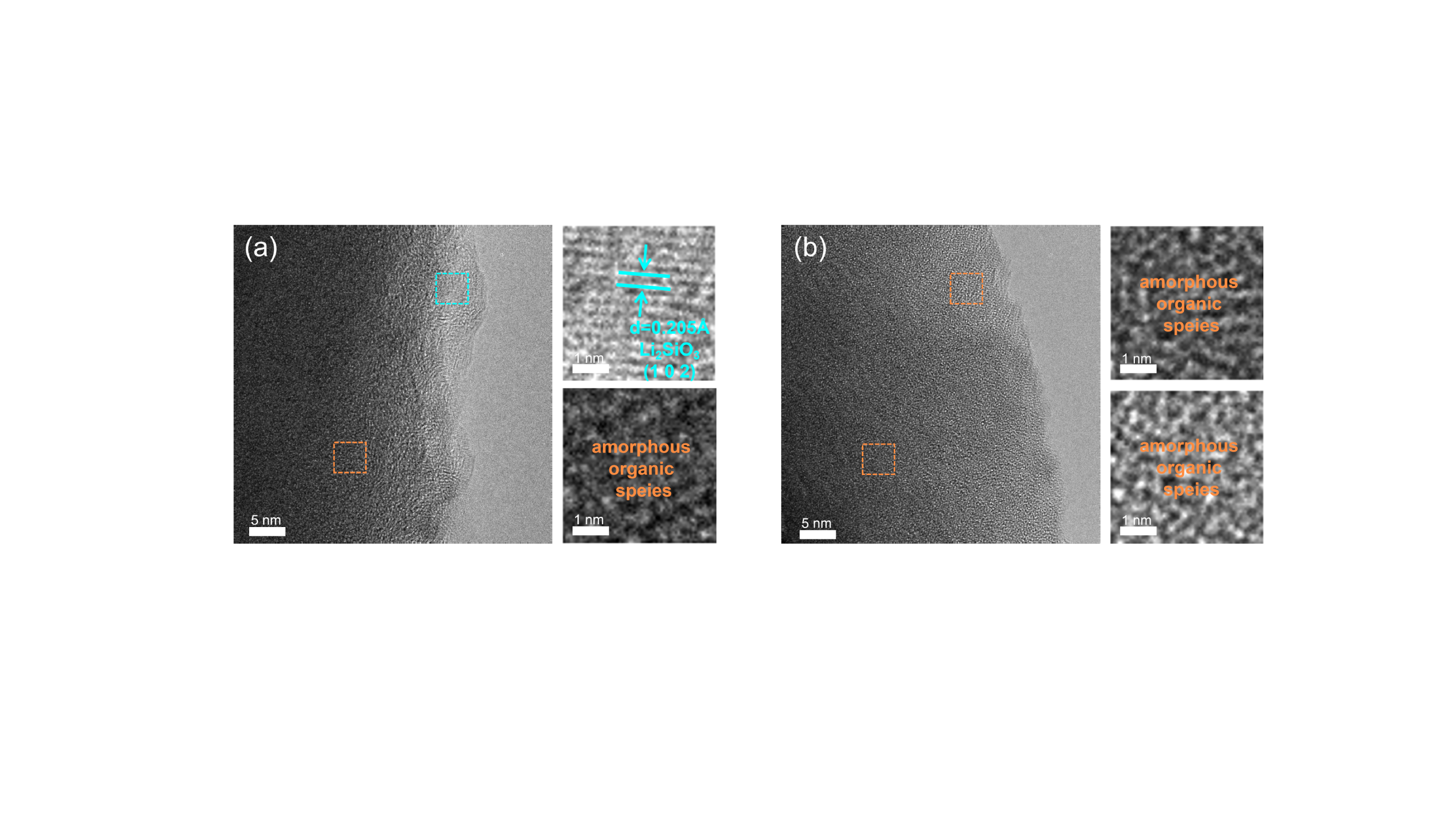


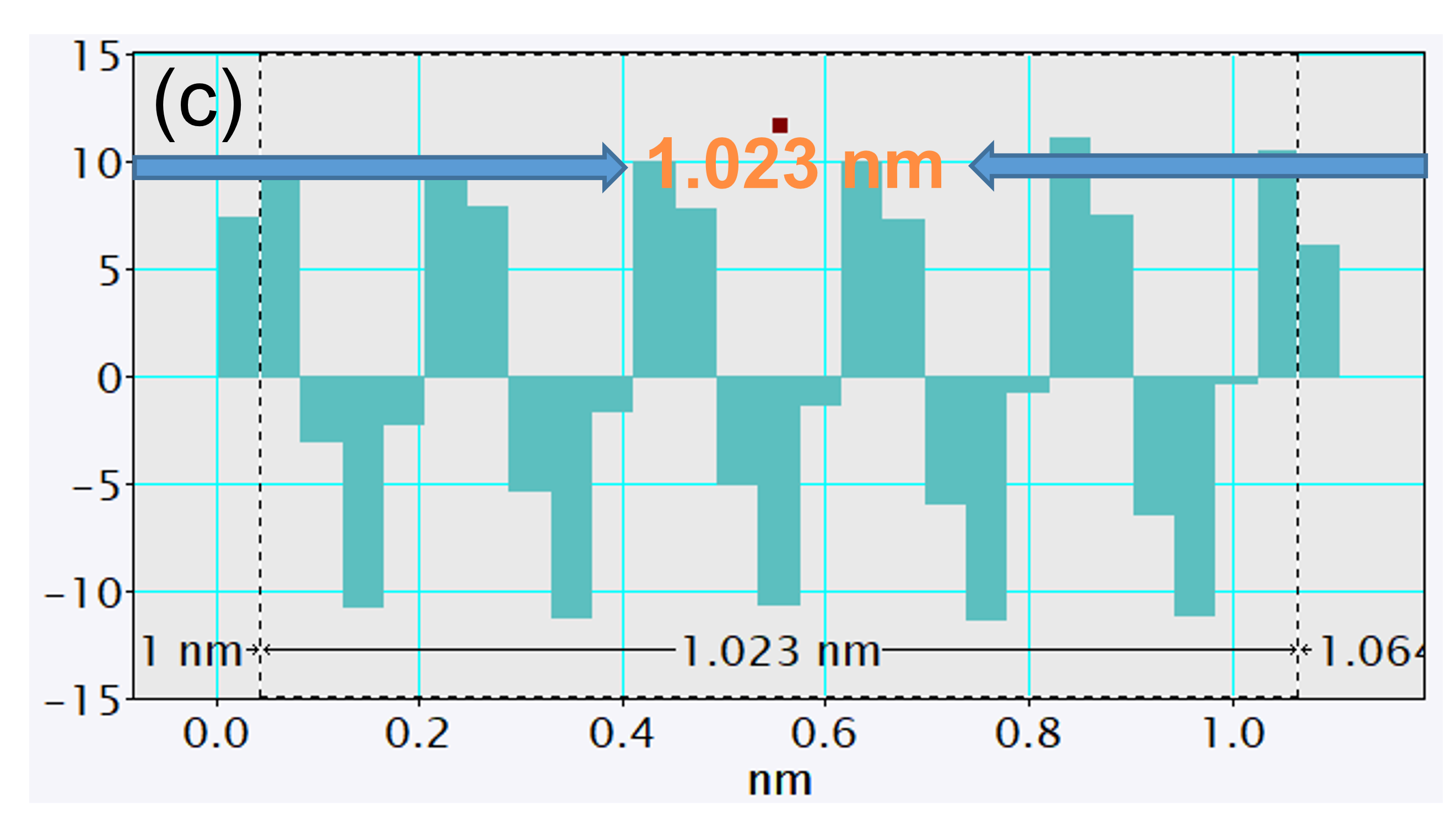


**Figure S14.** HR-TEM images of the (a) Pr-SiO and (b) SiO. (c) Lattice spacing analysis of Li-Si.

Crystal structure calibration based on the following PDF cards:

Li-Si:82-2202


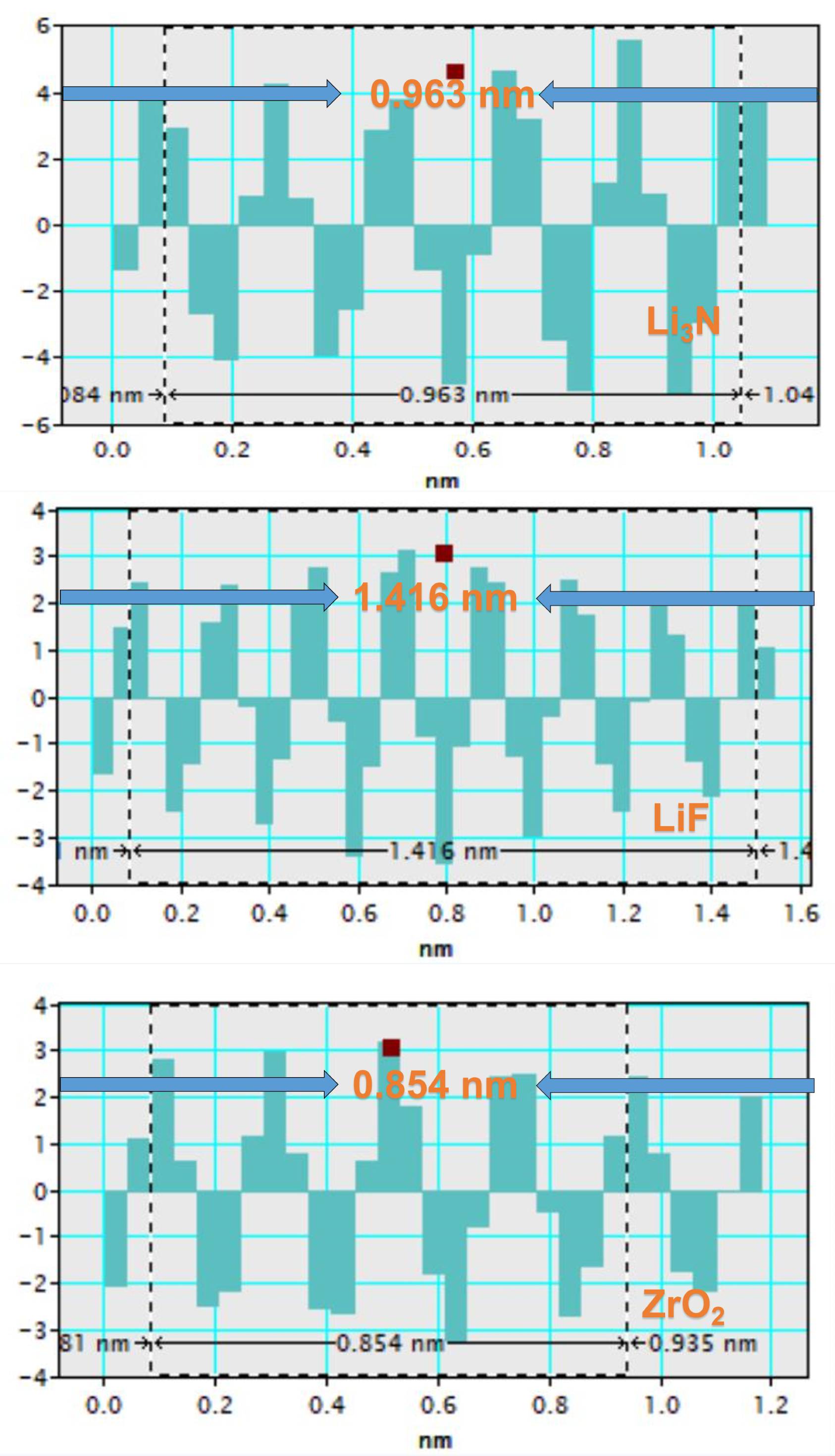


**Figure S15.** Lattice spacing analysis of Li_3_N, LiF and ZrO_2_.

Crystal structure calibration based on the following PDF cards:

Li_3_N: 76-0593

LiF: 04-0857

ZrO_2_: 49-1746


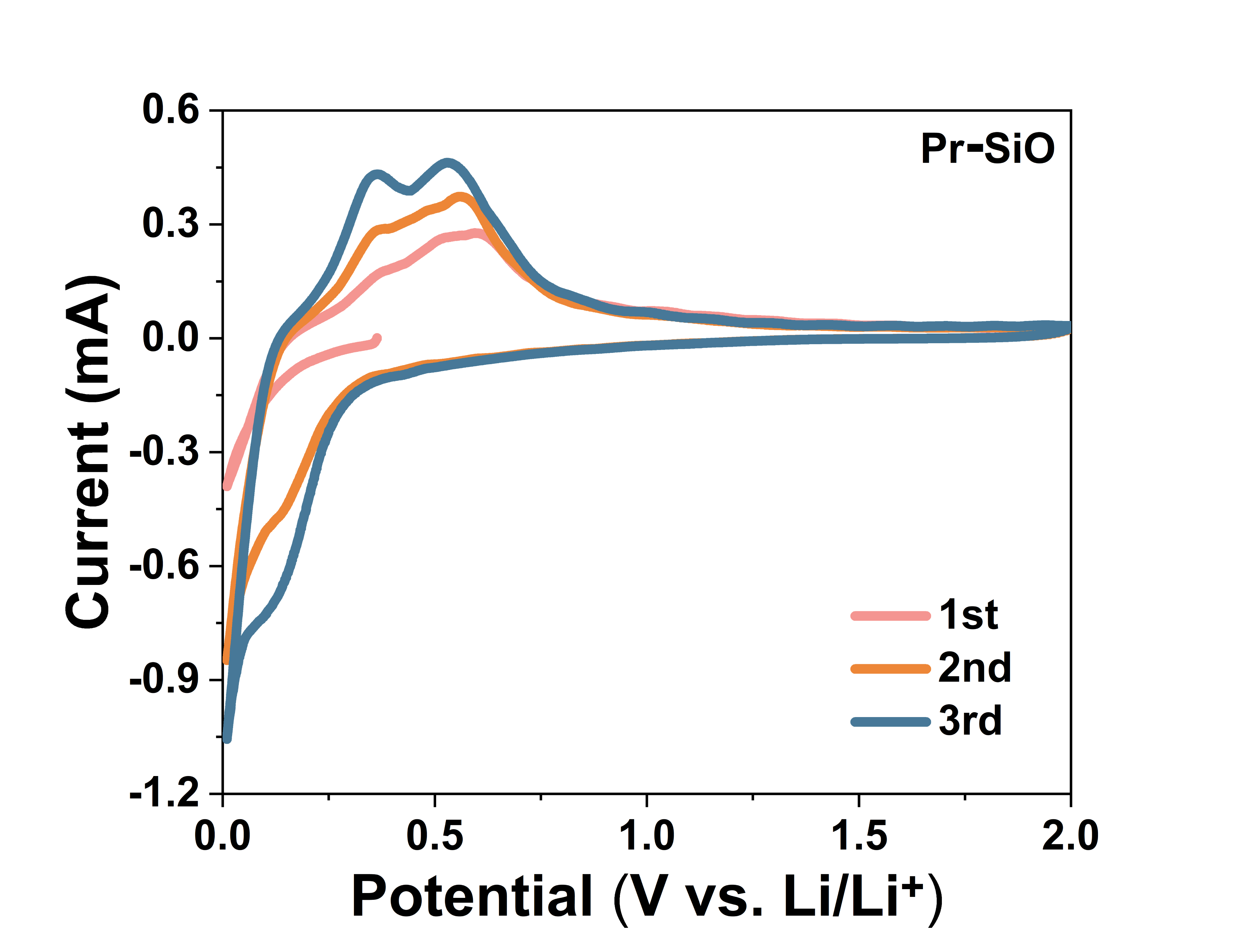

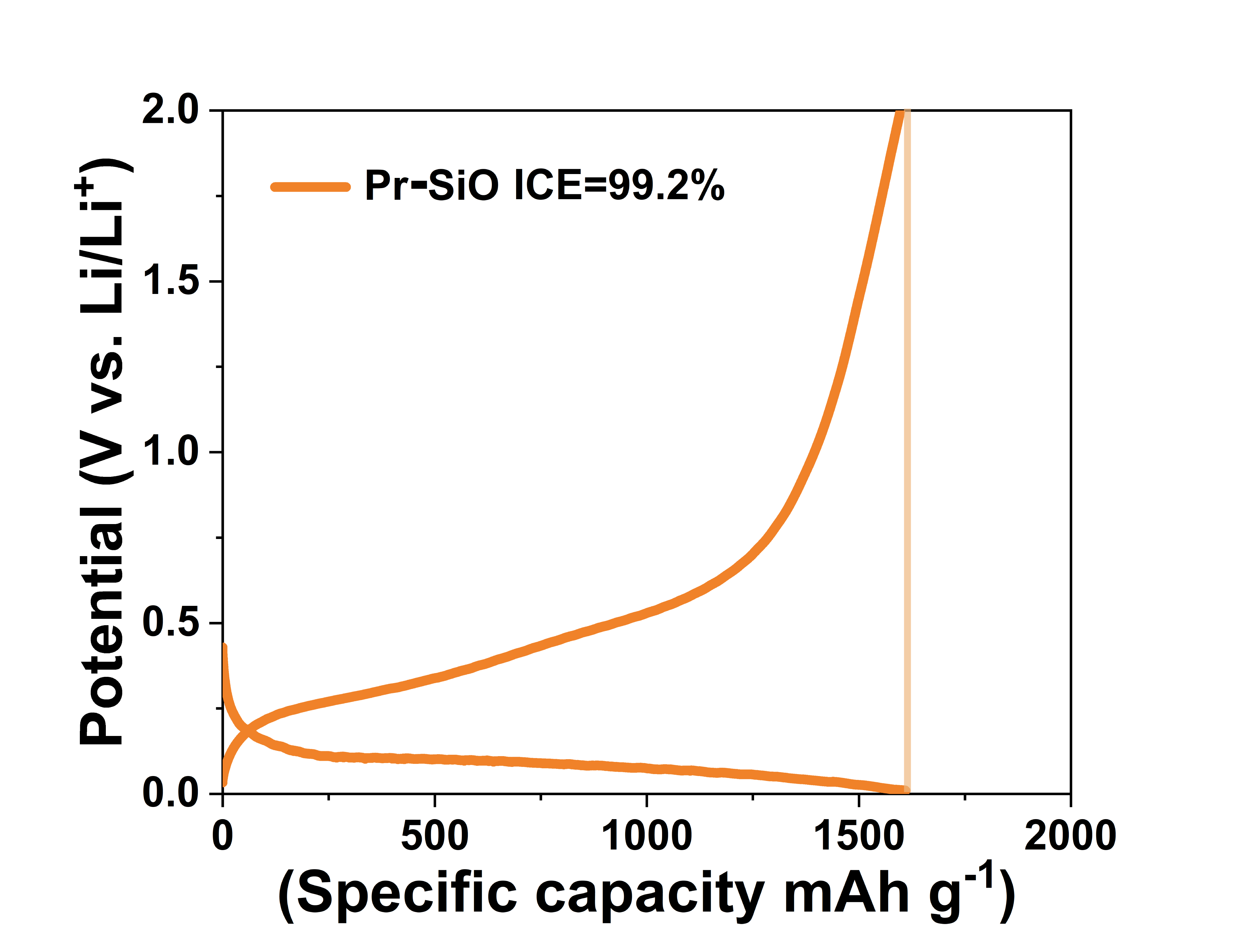


**Figure S16.** CV curves and Initial charge/discharge curves of Pr-SiO.

**
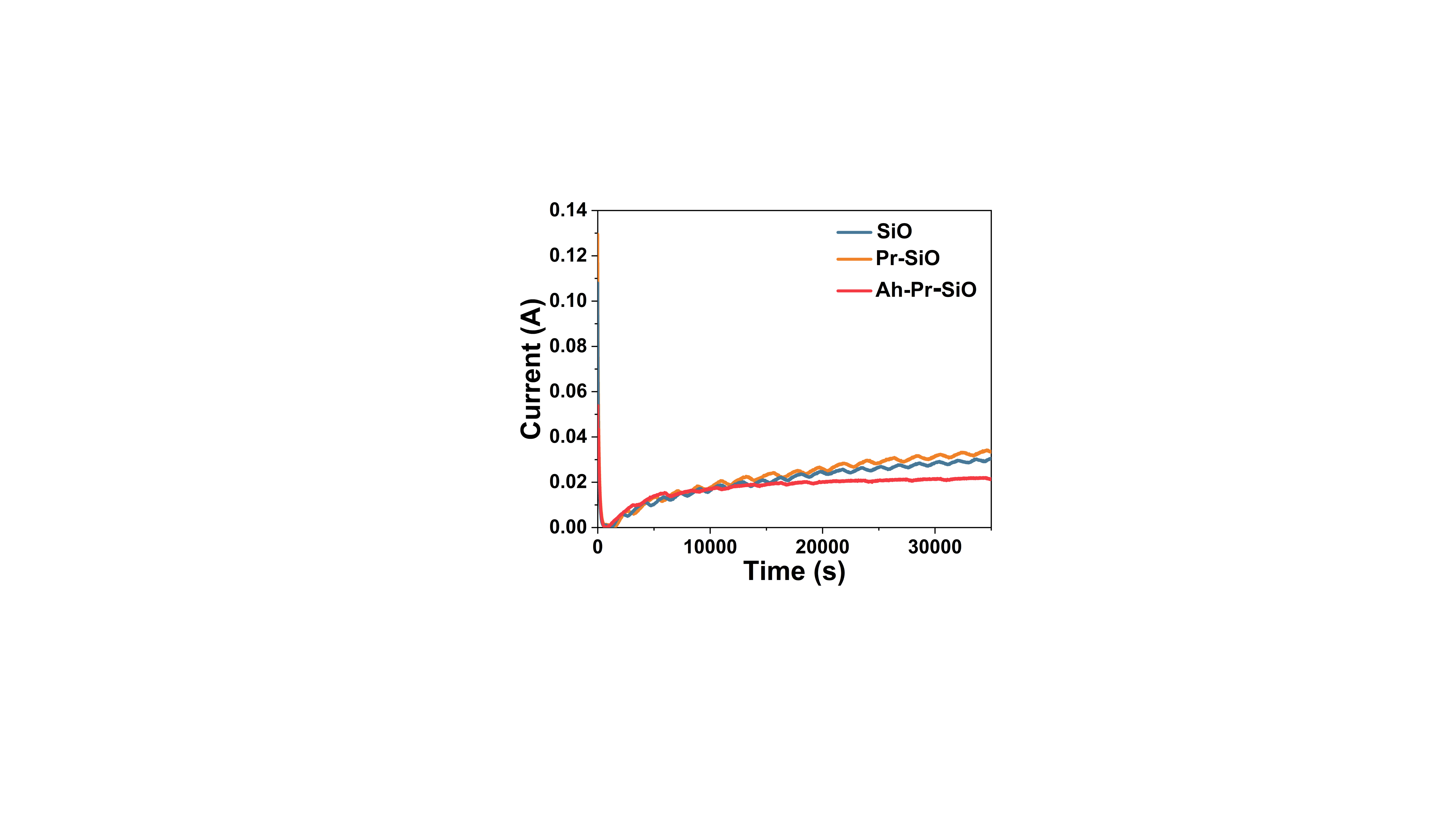
**

**Figure S17.** The DC polarization curves of SiO, Pr-SiO and Ah-Pr-SiO electrodes at 0.005 V.

**
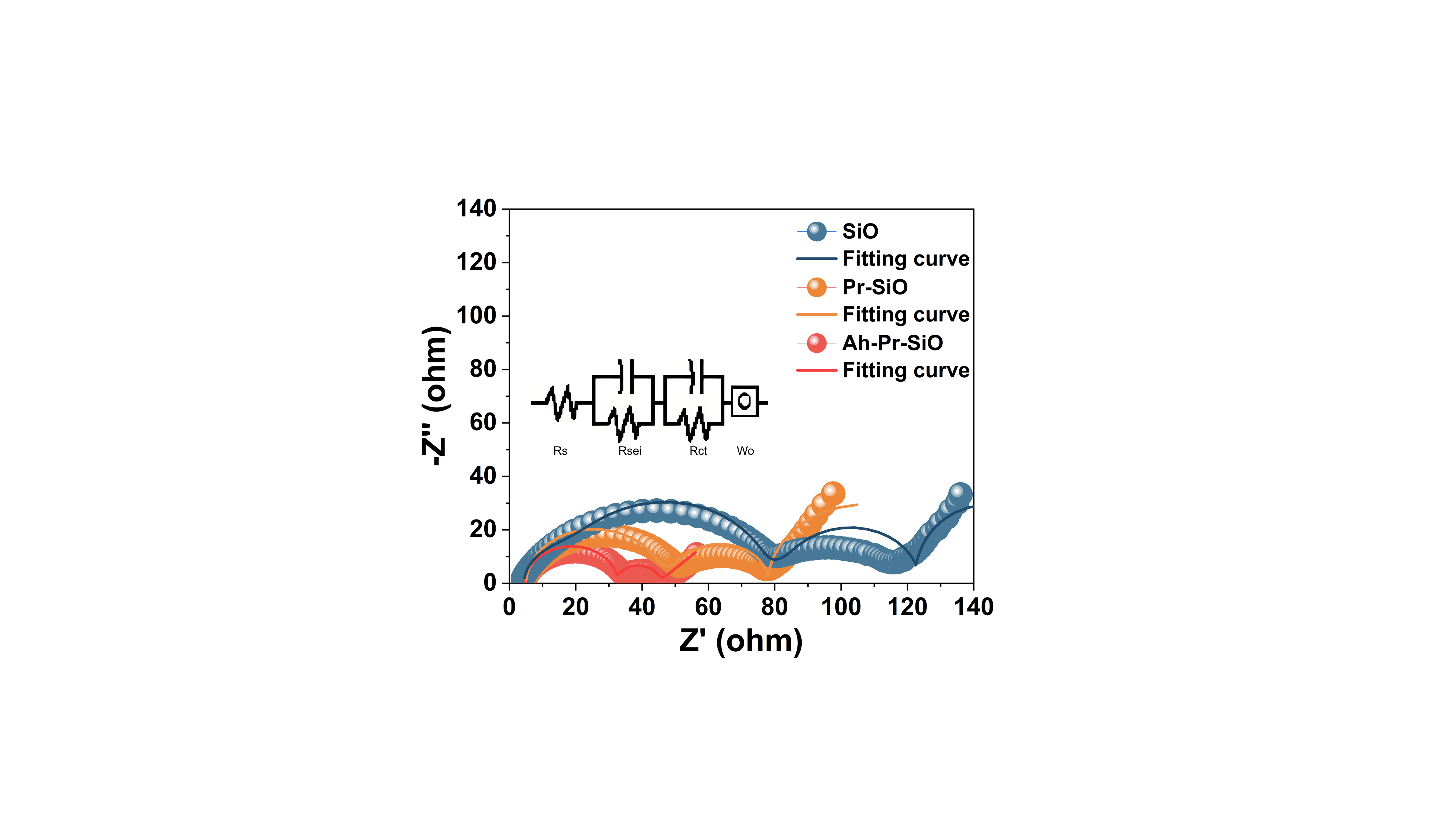
**

**Figure S18.** EIS measurements and the equivalent circle of the fitted Nyquist plot of SiO, Pr-SiO, and Ah-Pr-SiO at the first cycle, respectively.


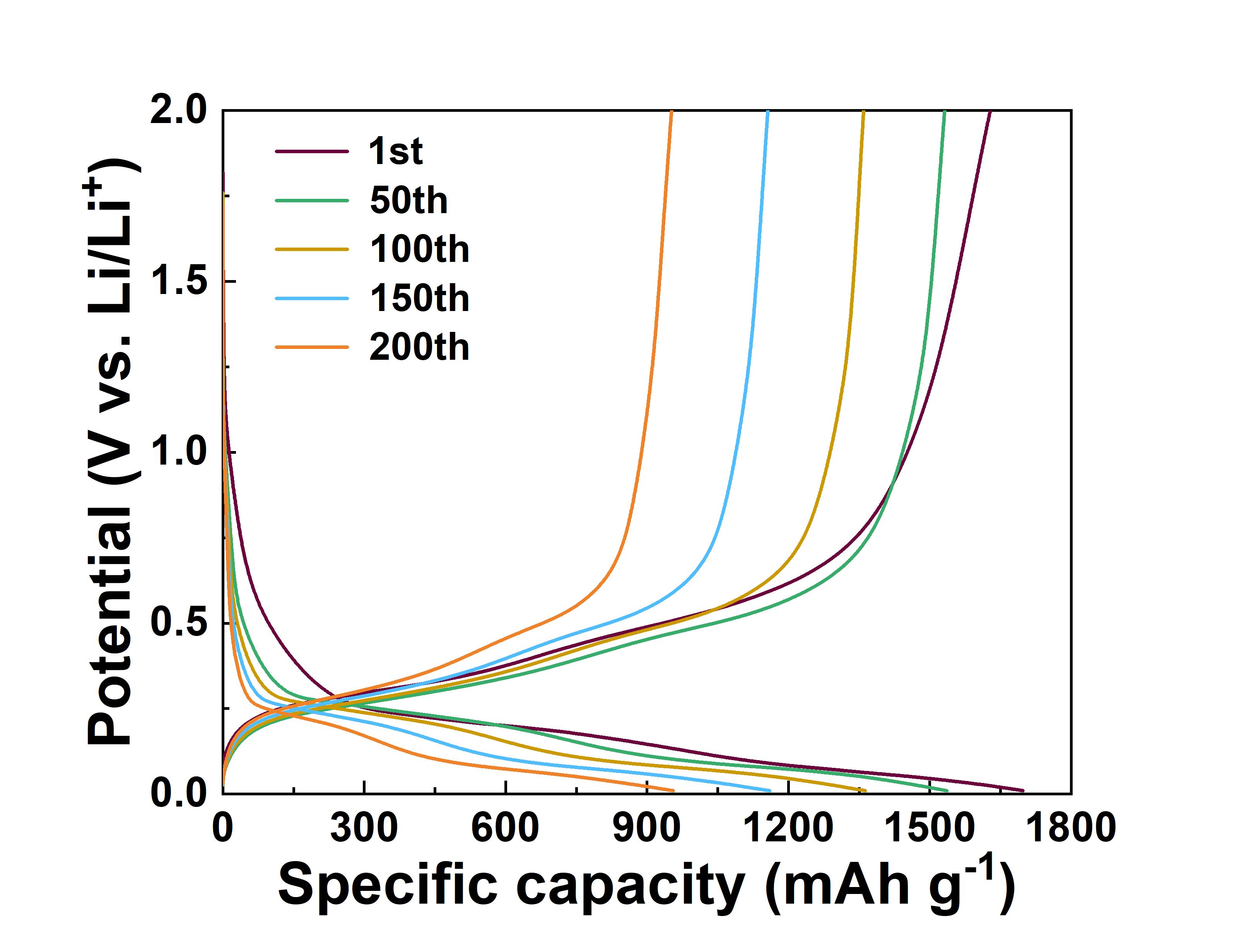


**Fig. S19.** The corresponding galvanostatic charging/discharging profiles of Ah-Pr-SiO.


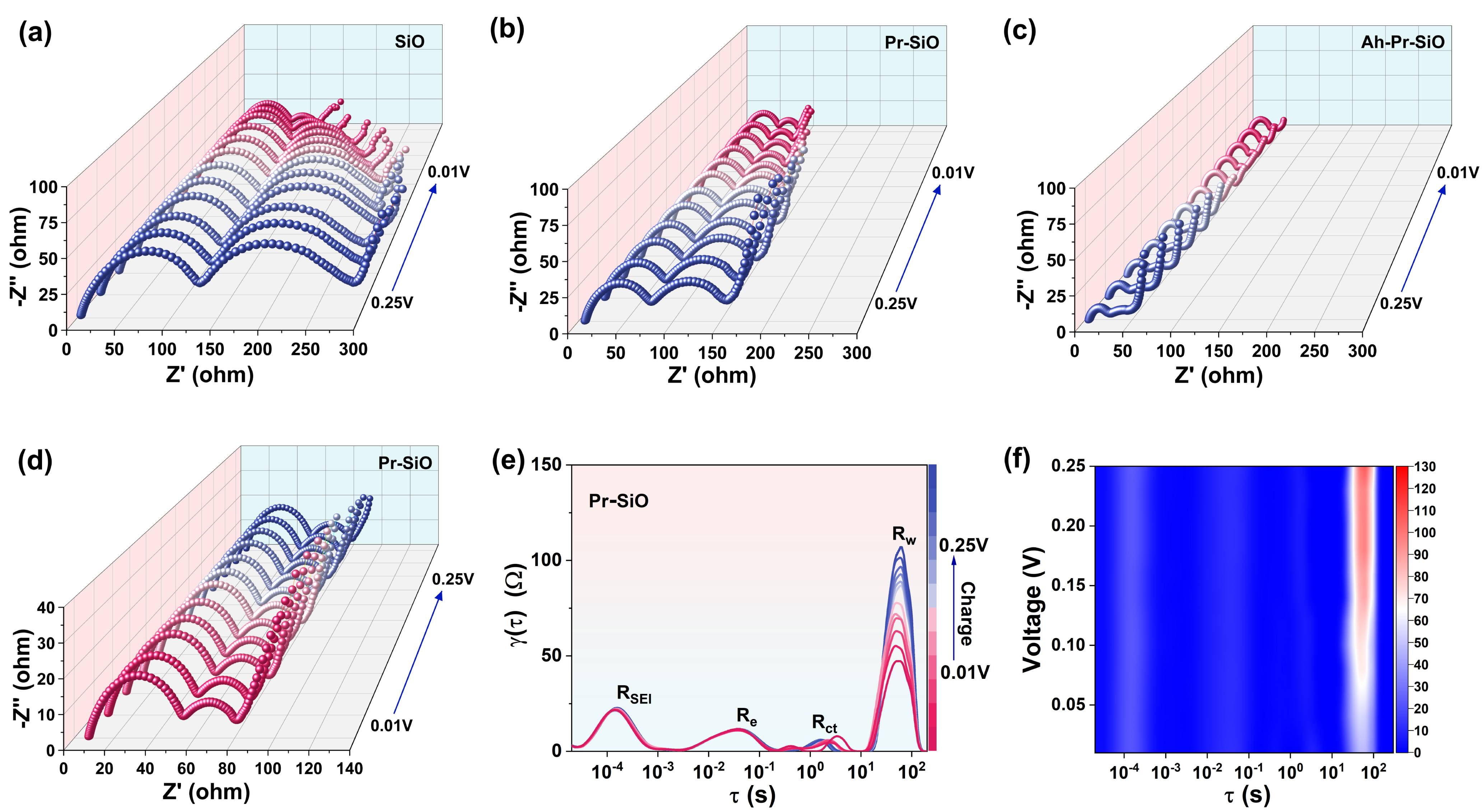


**Figure S20.** In-situ impedance of (a) SiO, (b) Pr-SiO and (c) Ah-Pr-SiO in 0.25-0.01V discharge at initial cycling.


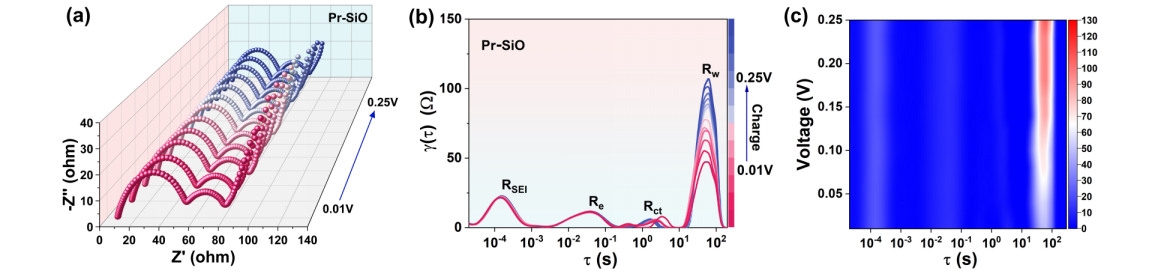


**Figure S21.** In-situ impedance of (a) Pr-SiO at initial charge process. (b) DRT curves during 0.01-0.25V charge Pr-SiO. (c) In situ DRT contour plots of Pr-SiO monitored in a 0.01-0.25 V charge at initial cycling


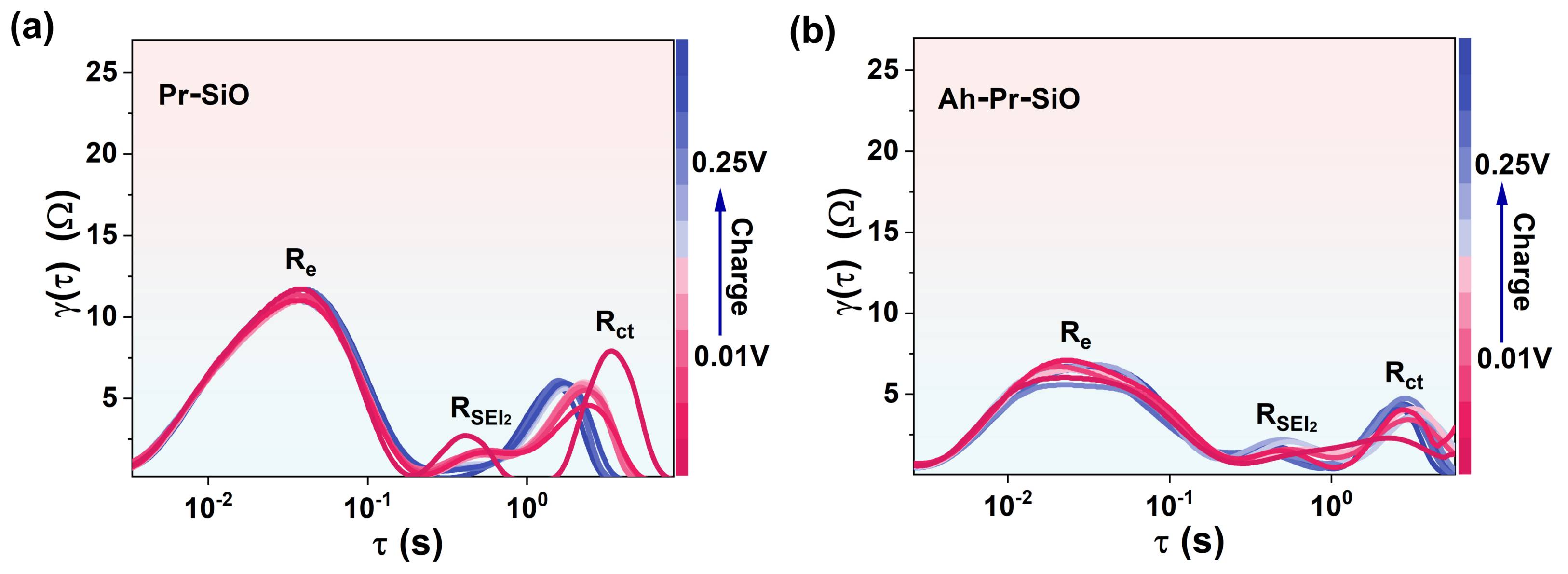


**Figure S22.** Enlarged image of DRT image of (a) Pr-SiO and (b) Ah-Pr-SiO.


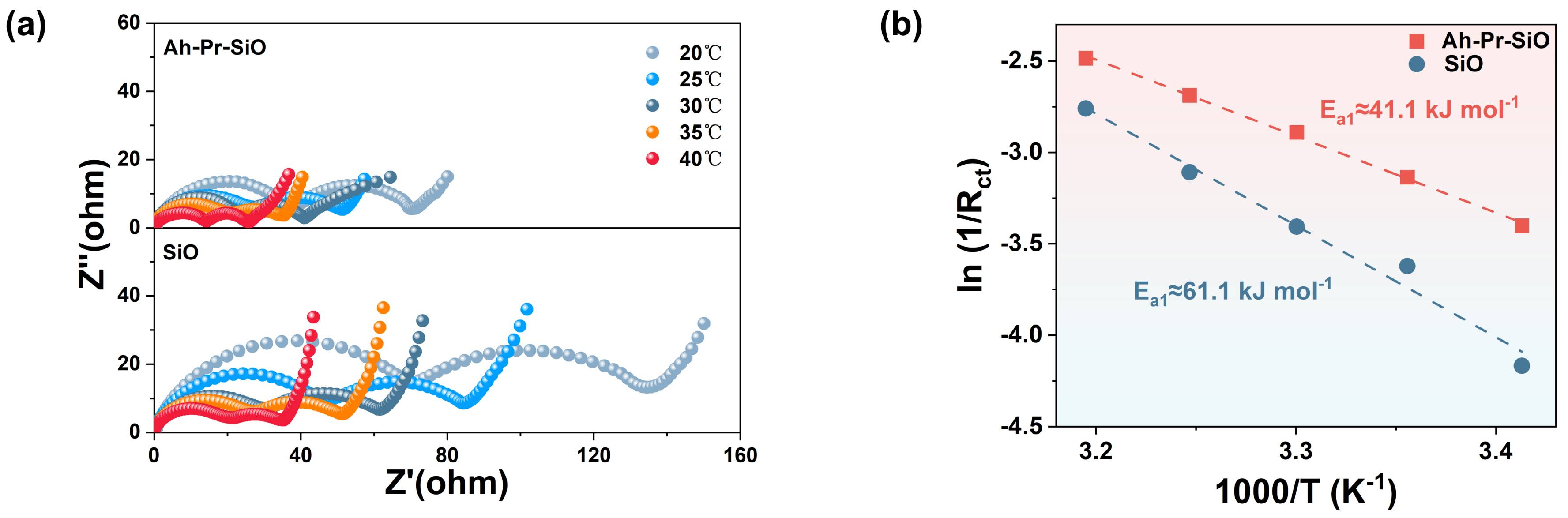


**Figure S23.** (a) EIS profiles at different temperatures and (b) Activation energies (Ea_1_) of Li^+^ desolvation.


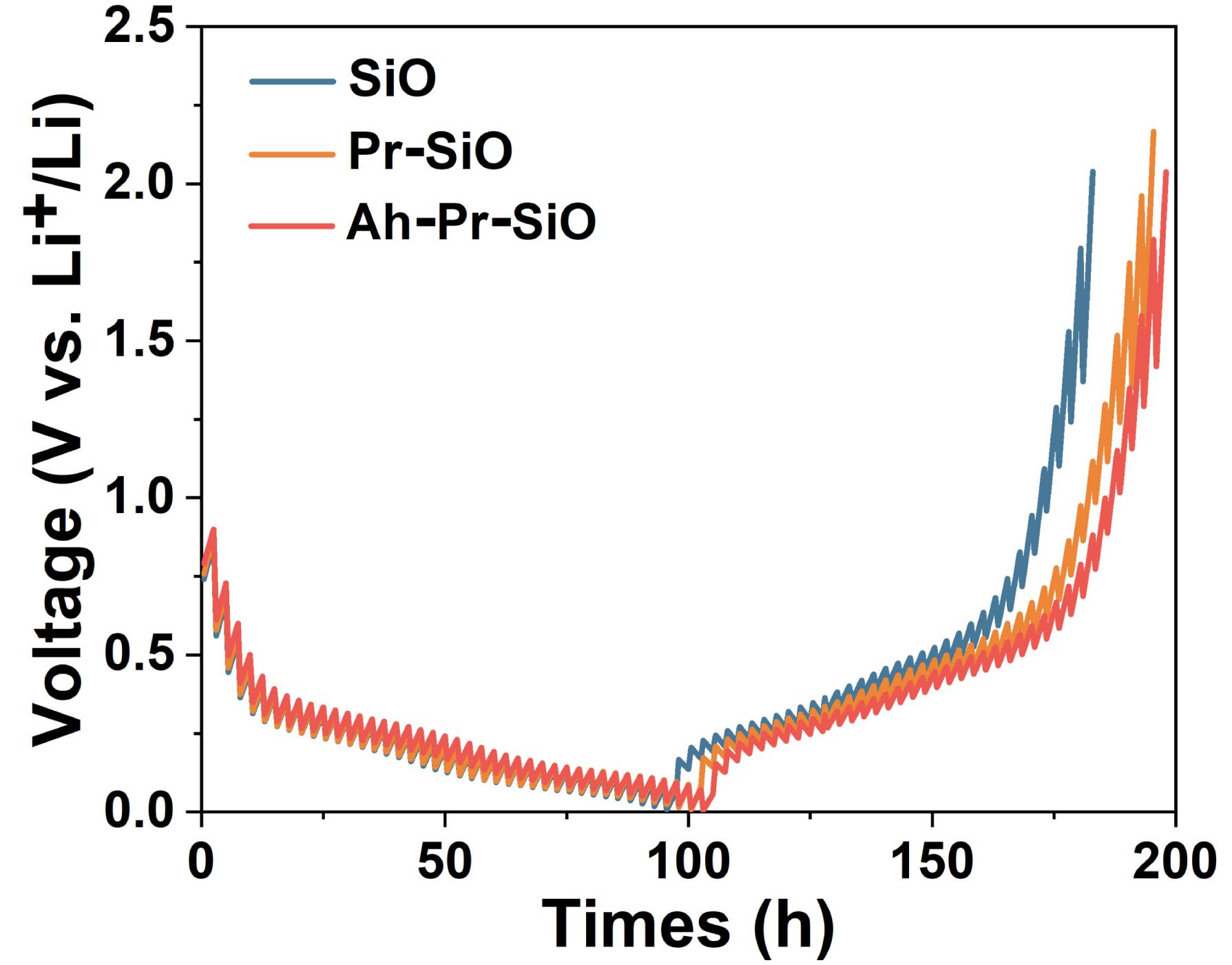


**Figure S24.** GITT voltage profiles of SiO, Pr-SiO, and Ah-Pr-SiO.


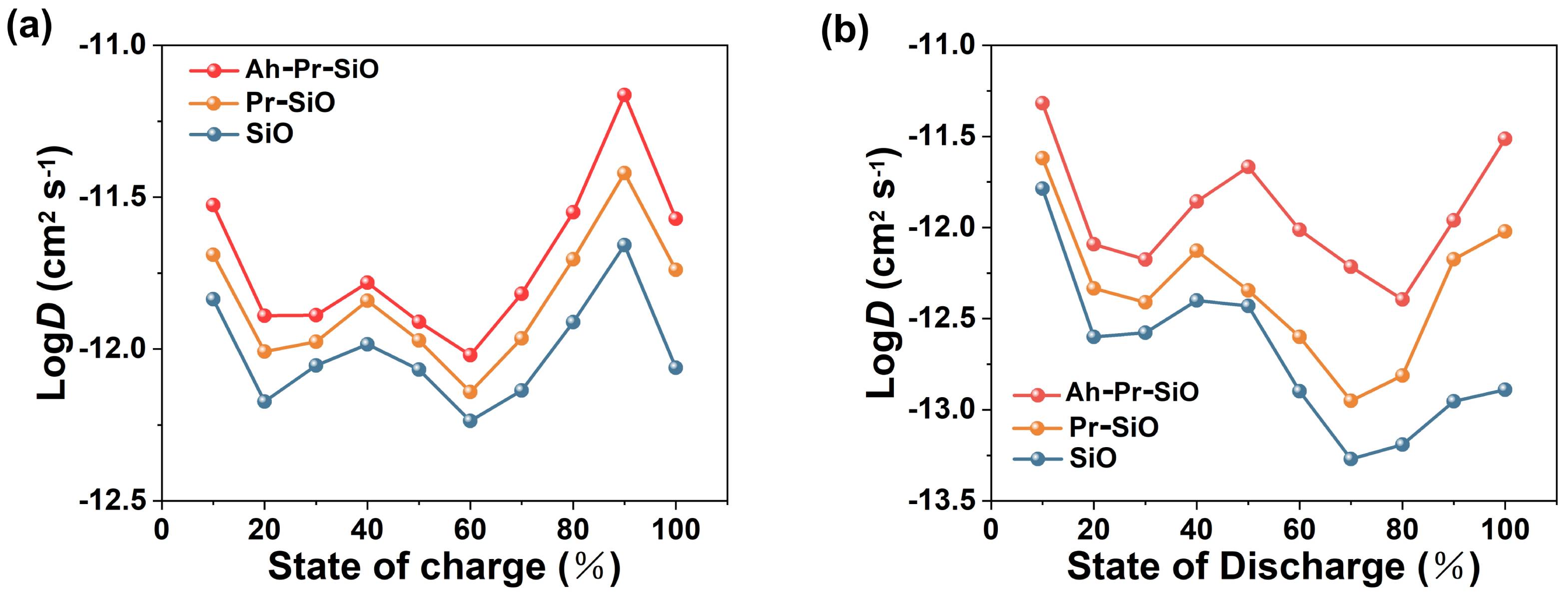


**Figure S25.** The calculated Li chemical diffusion coefficients in the (a) charge and (b) discharge states.


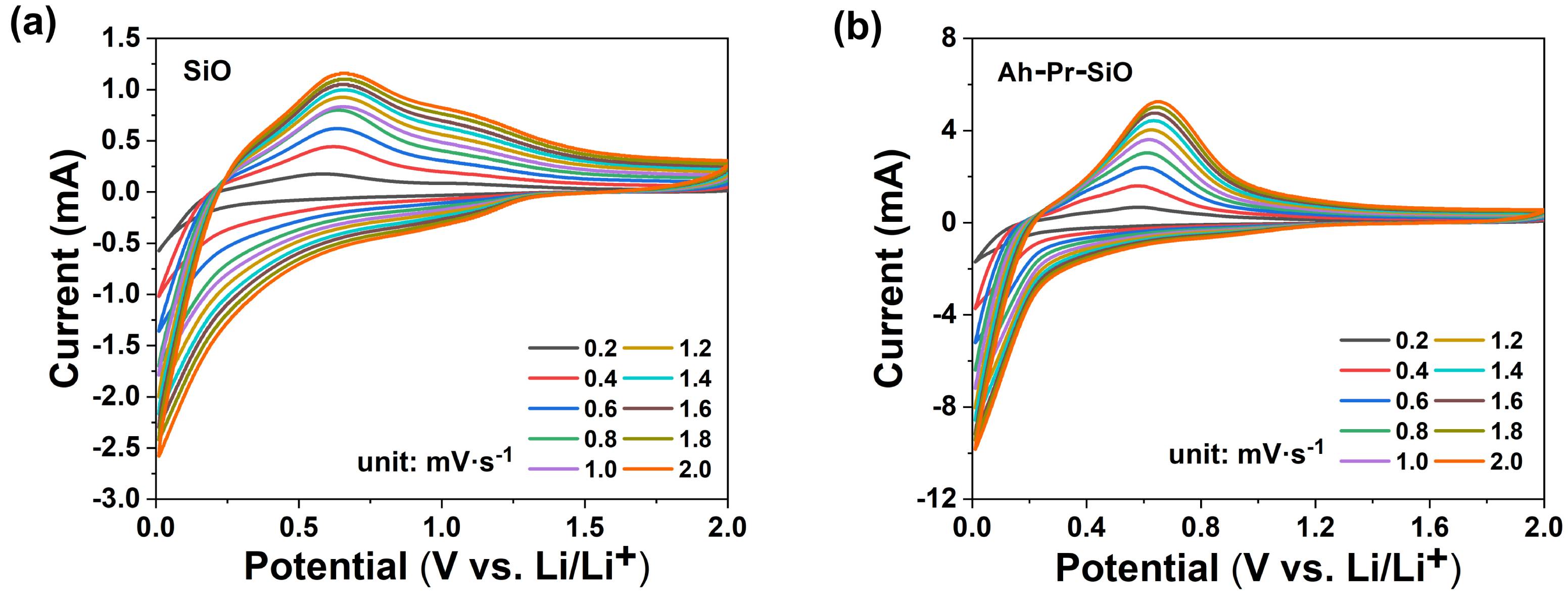


**Figure S26.** The CV curves of (a) SiO and (b) Ah-Pr-SiO at different scan rates.


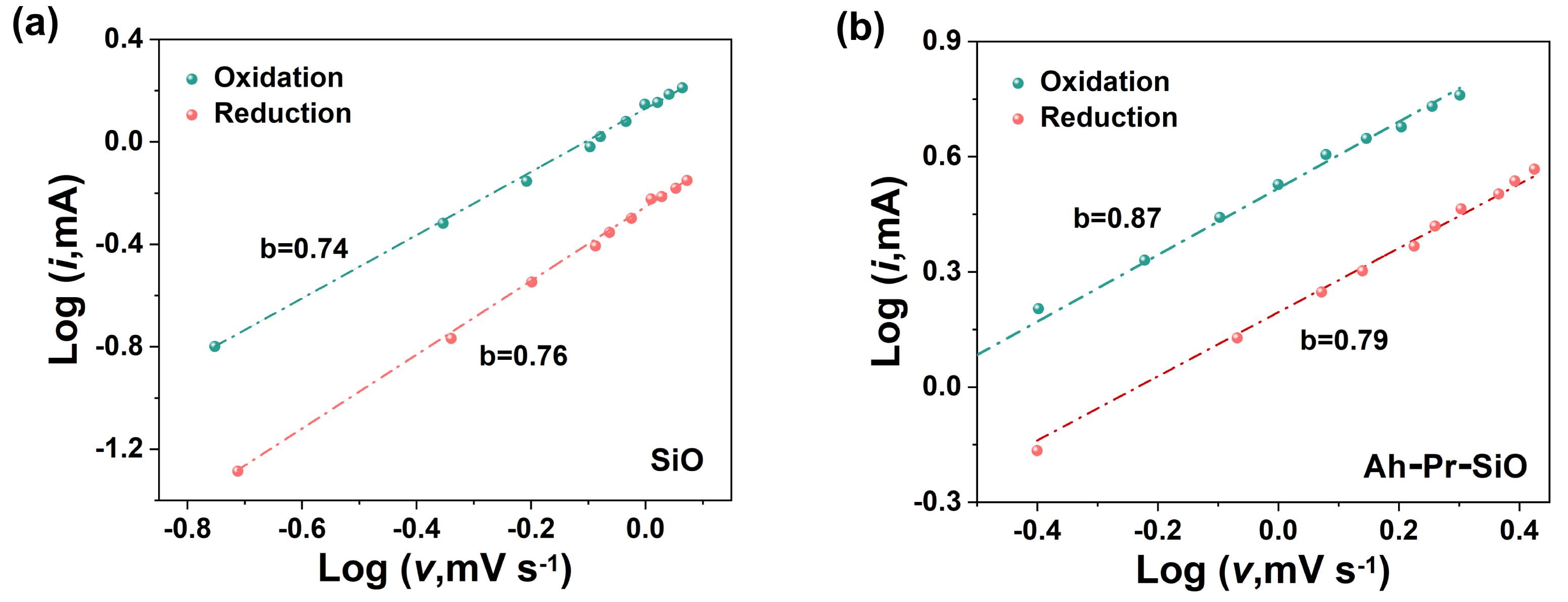


**Figure S27.** *b* value tests of (a) SiO and (b) Ah-Pr-SiO.

**
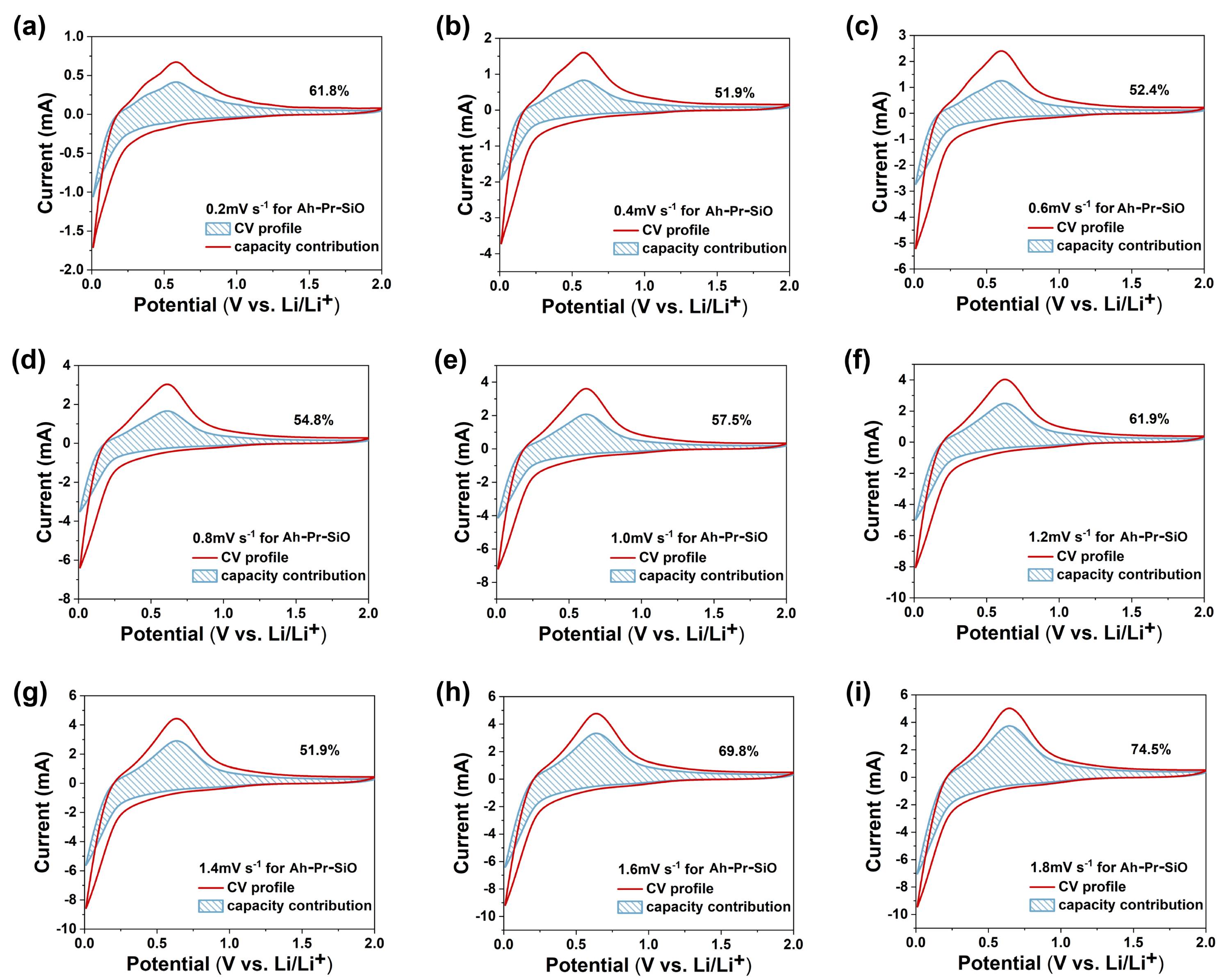
**

**Figure S28.**Capacitive contributions at different scan rates of the Ah-Pr-SiO anodes. The scan rates are (a) 0.2 mV s^-1^; (b) 0.4 mV s^-1^; (c) 0.6 mV s^-1^; (d) 0.8 mV s^-1^; (e) 1 mV s^-1^; (f) 1.2 mV s^-1^; (g) 1.4 mV s^-1^; (h) 1.6 mV s^-1^; (i) 1.8 mV s^-1^, respectively.


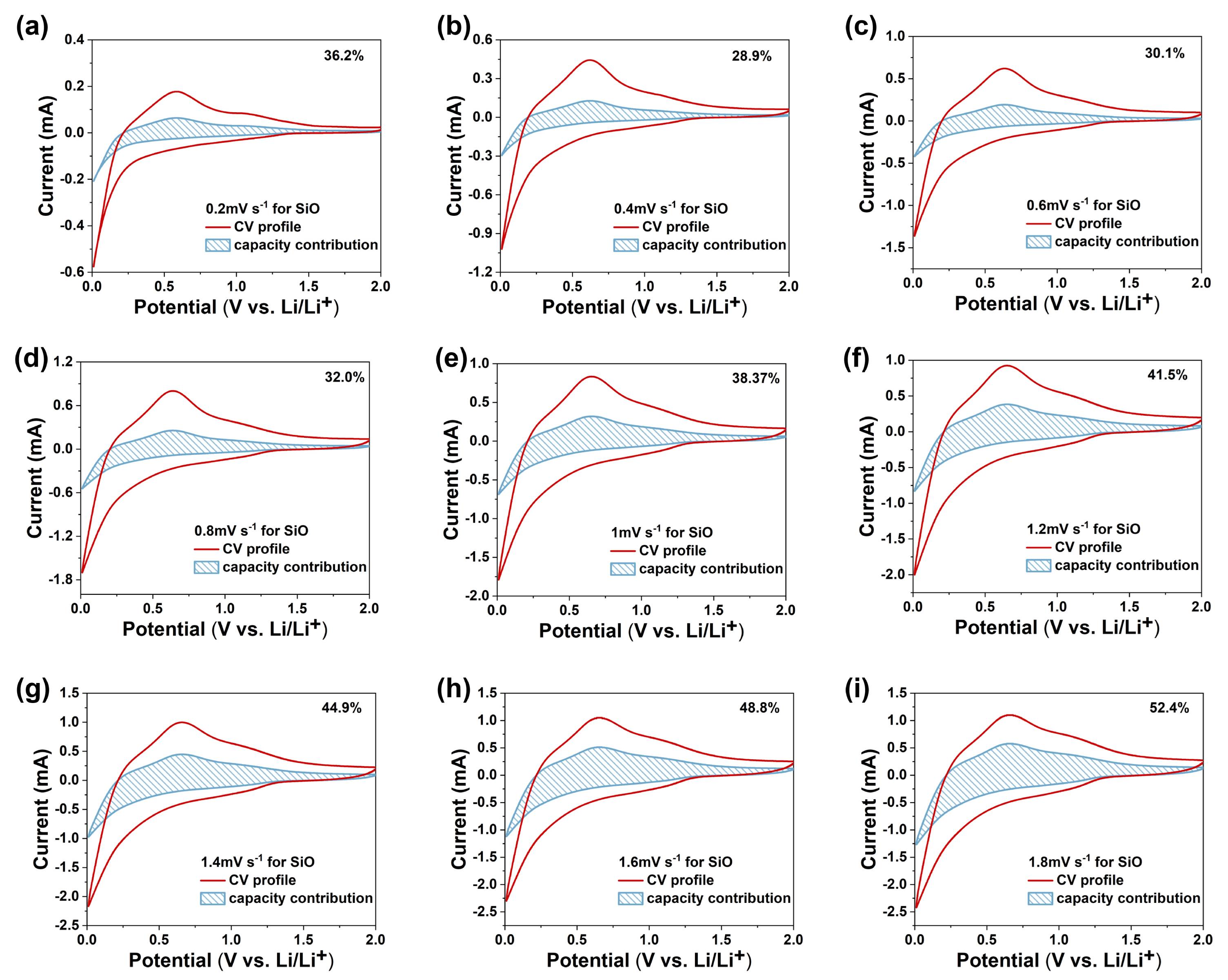


**Figure S29.**Capacitive contributions at different scan rates of the SiO anodes. The scan rates are (a) 0.2 mV s^-1^; (b) 0.4 mV s^-1^; (c) 0.6 mV s^-1^; (d) 0.8 mV s^-1^; (e) 1 mV s^-1^; (f) 1.2 mV s^-1^; (g) 1.4 mV s^-1^; (h) 1.6 mV s^-1^; (i) 1.8 mV s^-1^, respectively.


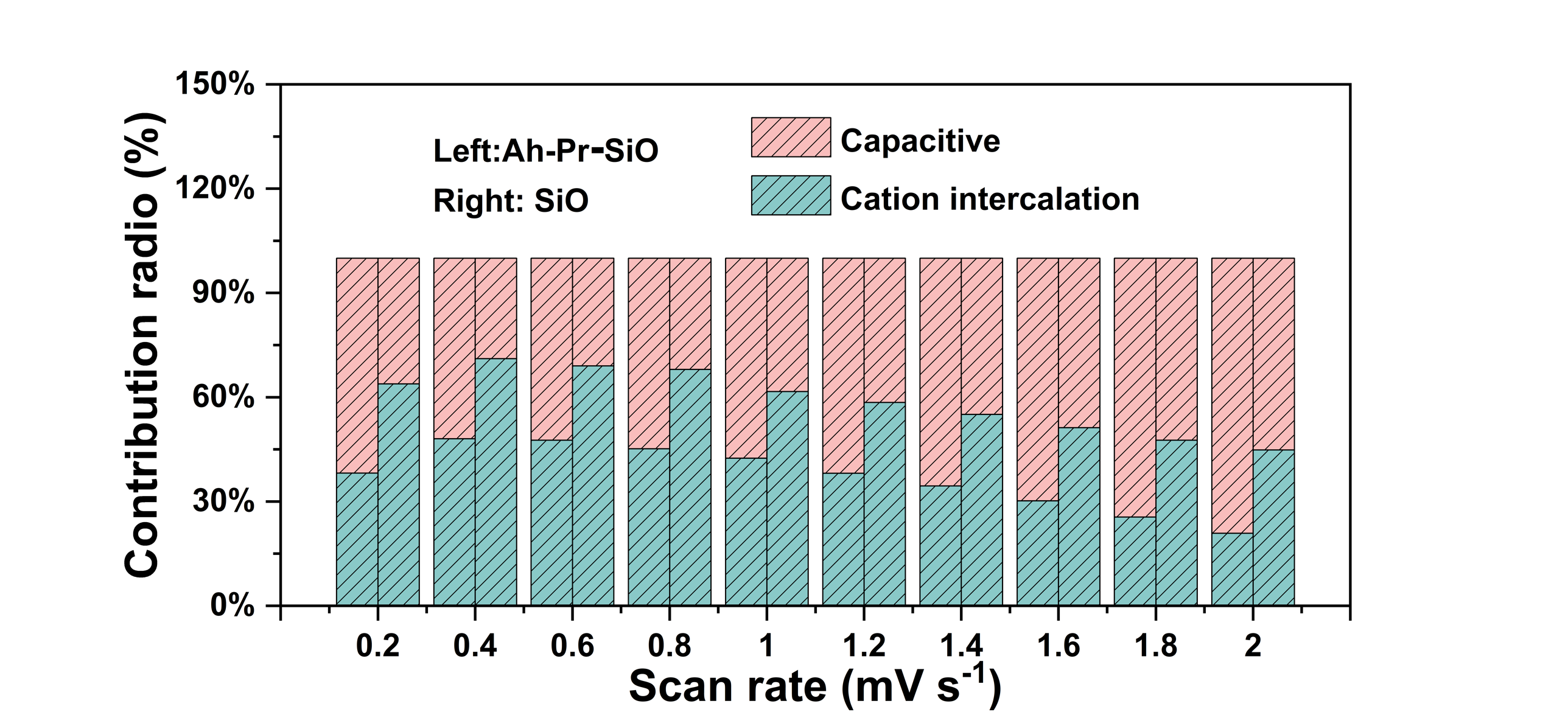


**Figure S30.** Capacitive contribution ratio at various sweep rates of SiO and Ah-Pr-SiO.


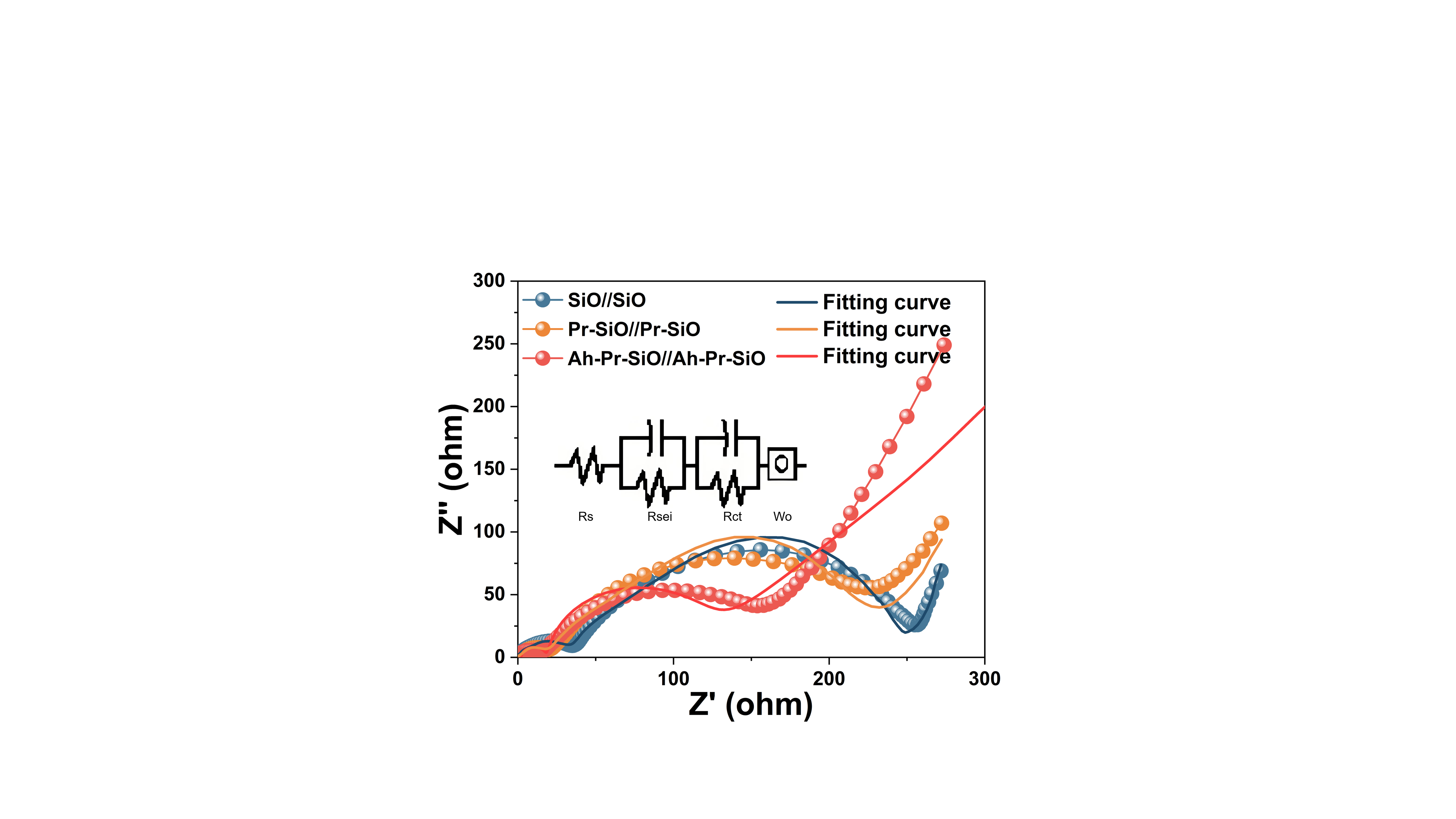


**Figure S31.** EIS measurements and the equivalent circle of the fitted Nyquist plot of SiO//SiO, Pr-SiO//Pr-SiO and Ah-Pr-SiO//Ah-Pr-SiO, respectively.

**
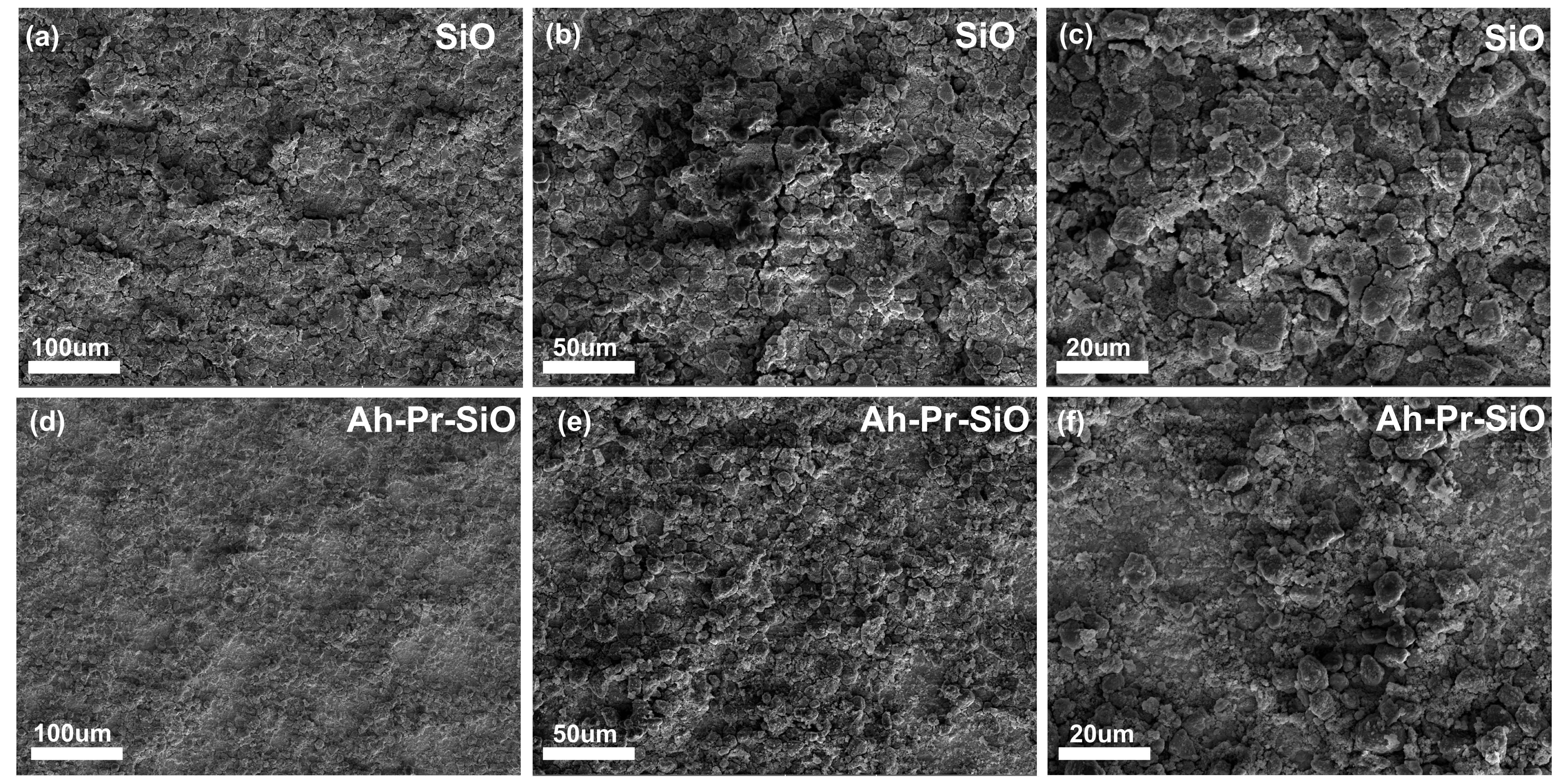
**

**Figure S32.** SEM images of (a)-(c) SiO and (d)-(e) Ah-Pr-SiO after 200 cycles.

**
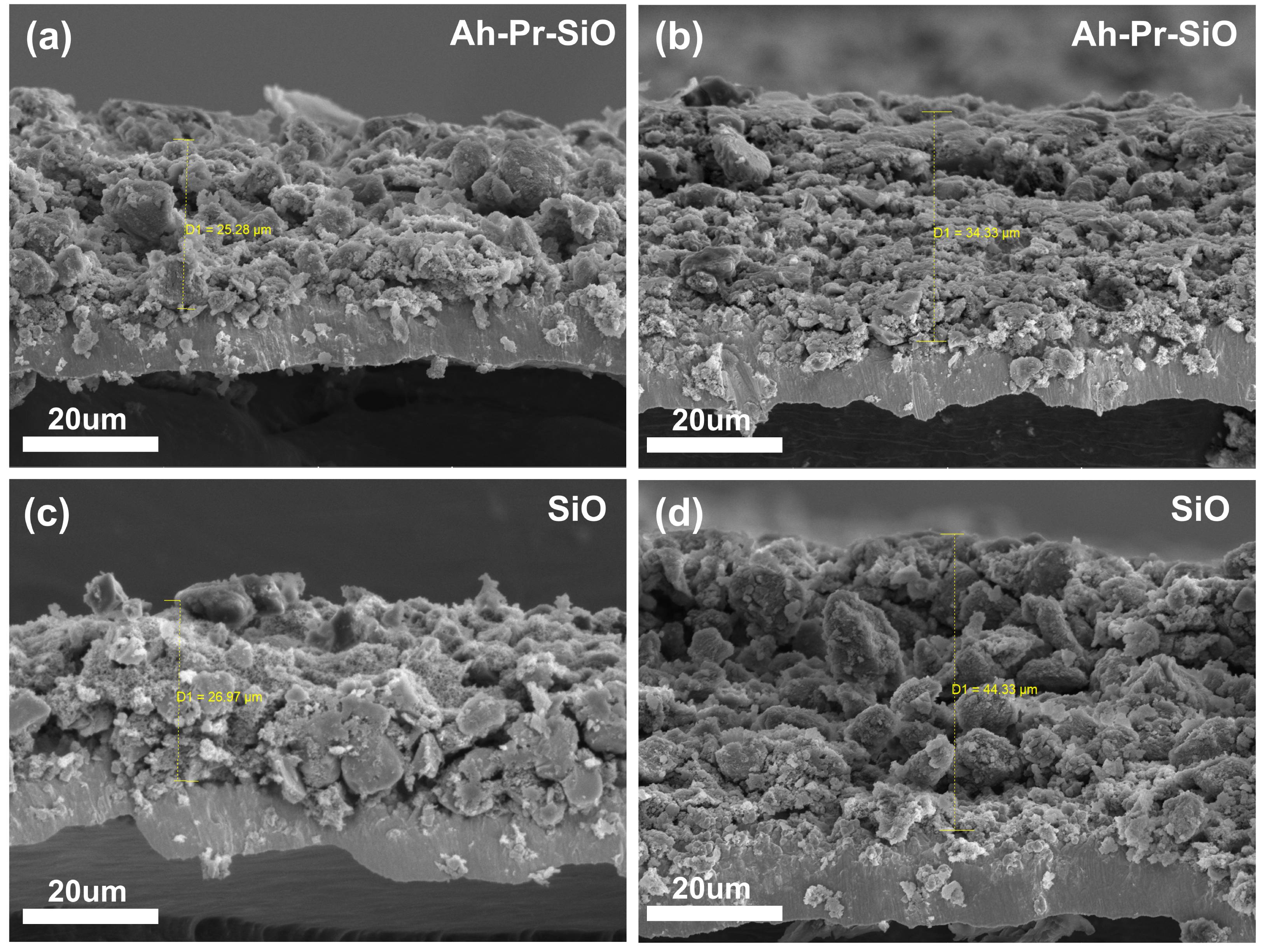
**

**Figure S33.** Corresponding cross-sectional SEM images of (a) Ah-Pr-SiO and (c) SiO anodes before cycling and (b) and (d) after 200 cycles.


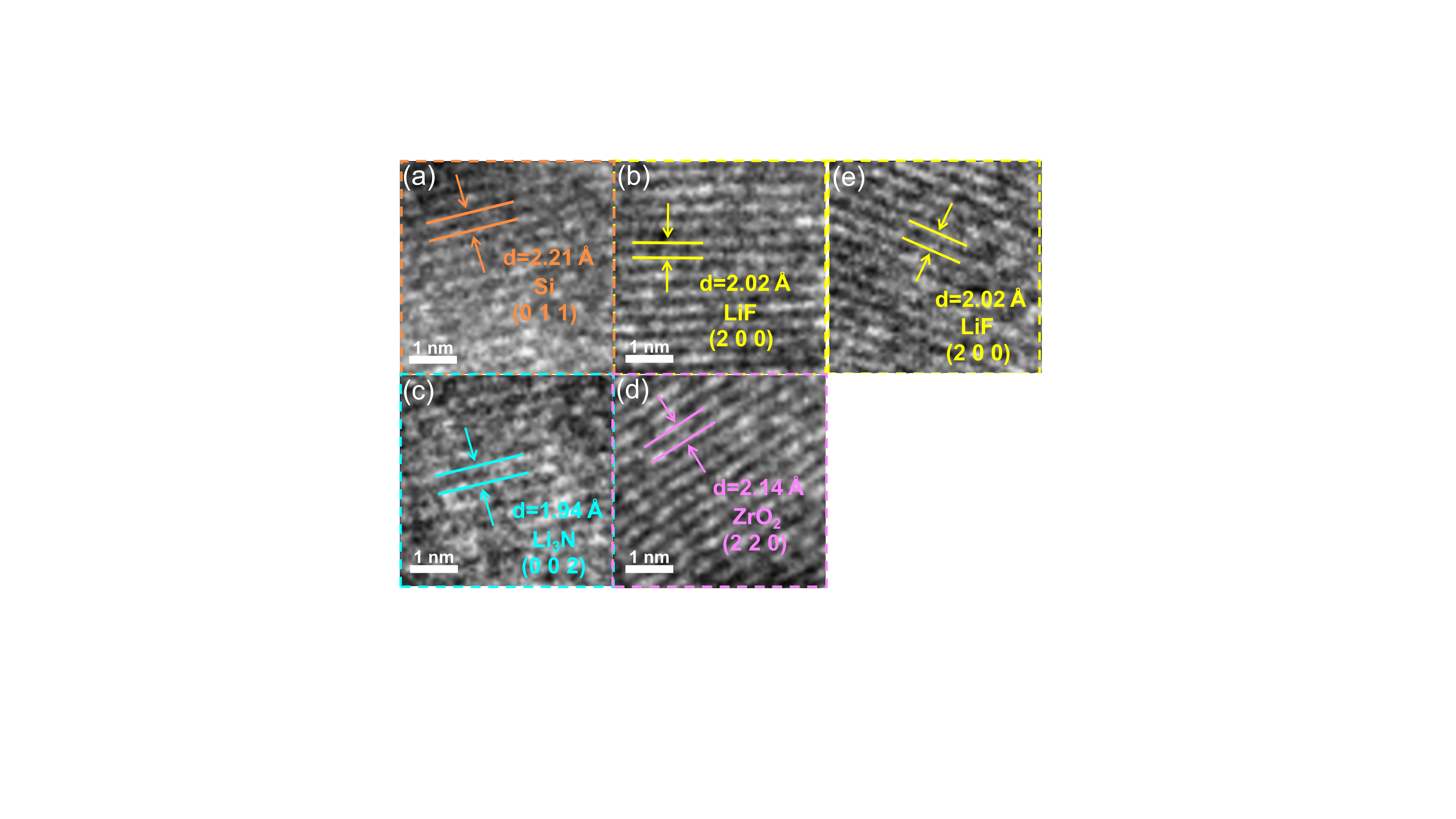


**Figure S34.** Magnified images of Region (a), Region (b), Region (c), Region (d) and Region (e) outlined in Figure 5 (a) and (b).

**
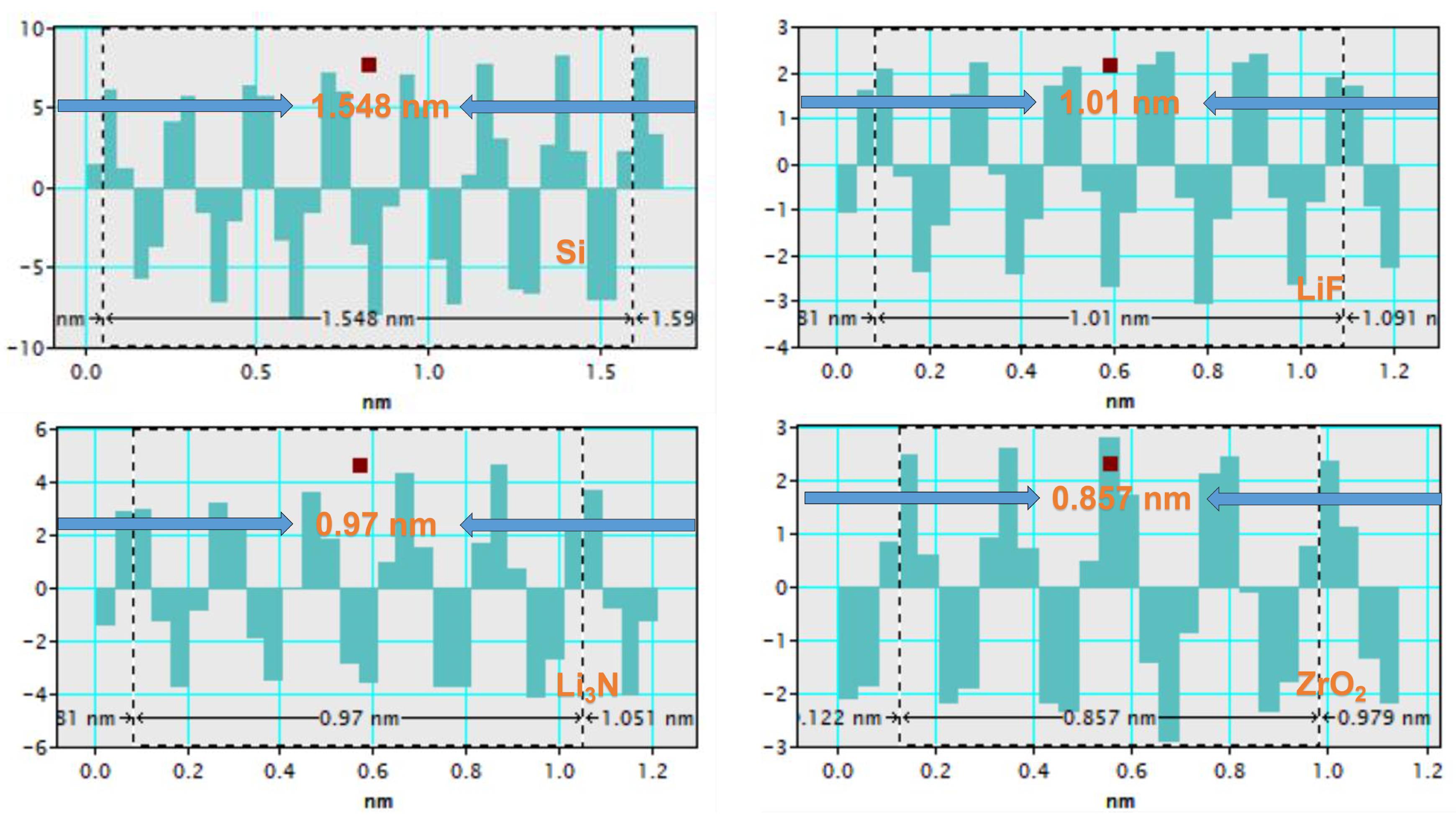
**

**Figure S35.** Lattice spacing analysis of Si, Li_3_N, LiF and ZrO_2_.

Crystal structure calibration based on the following PDF cards:

Si: 88-2284

Li_3_N: 76-0593

LiF: 04-0857

ZrO_2_: 49-1746


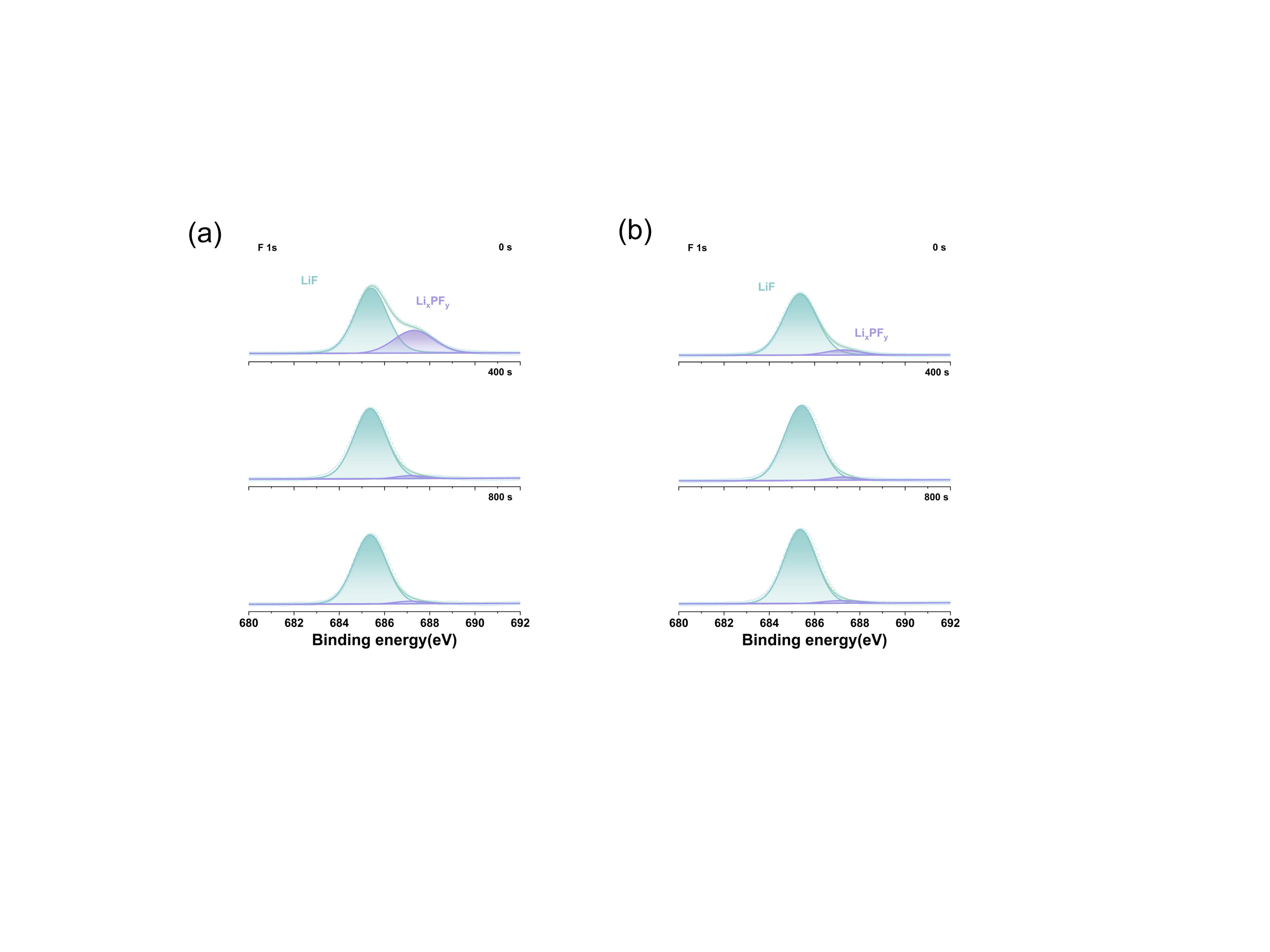


**Figure S36.** The F 1s XPS spectra depth profiles of the (a) SiO and (b) Pr-SiO.


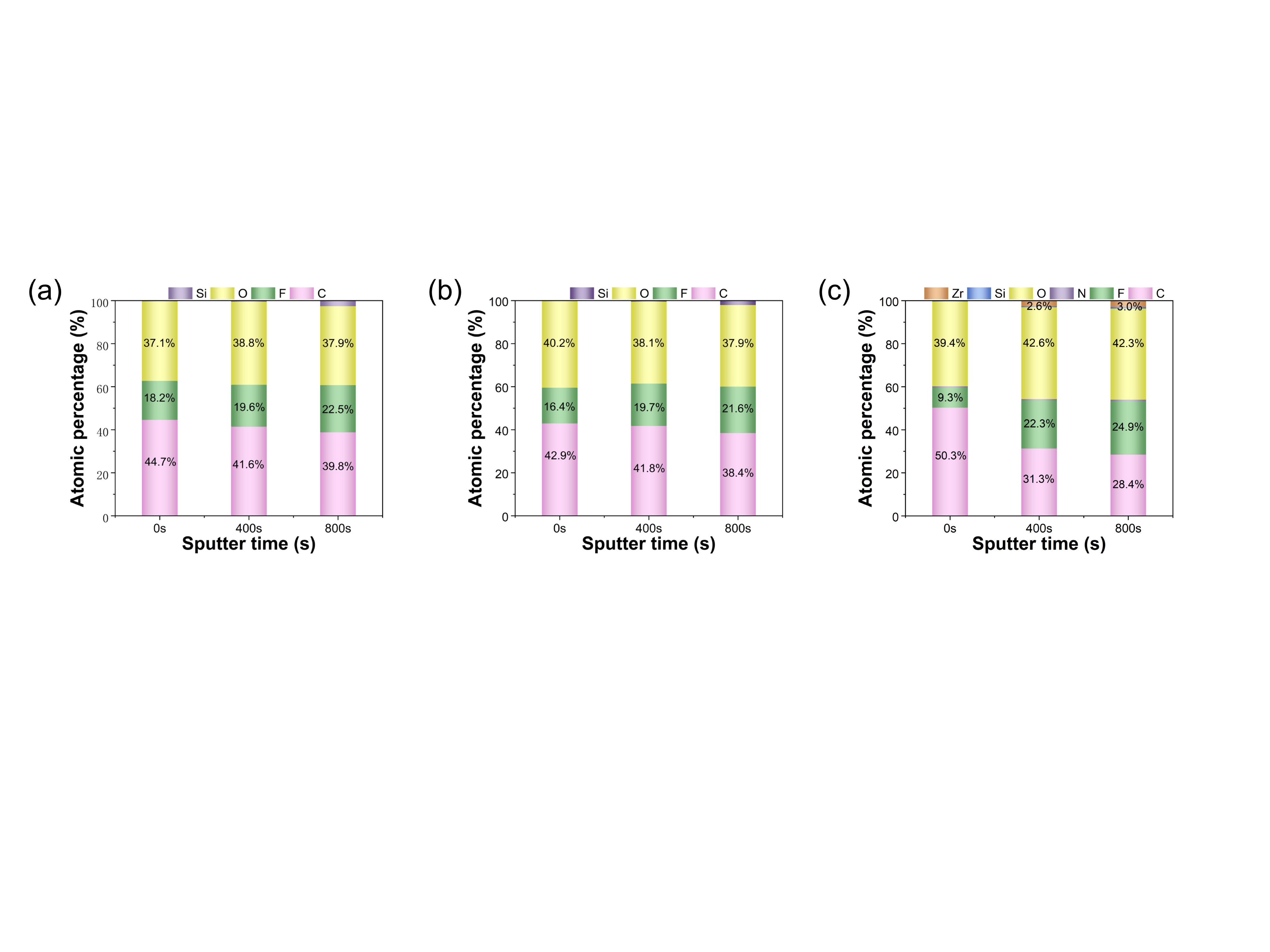


**Figure S37.** Depth evolution of the SEI element signals from (a) SiO, (b) Pr-SiO and (c) Ah-Pr-SiO electrode, respectively.

**
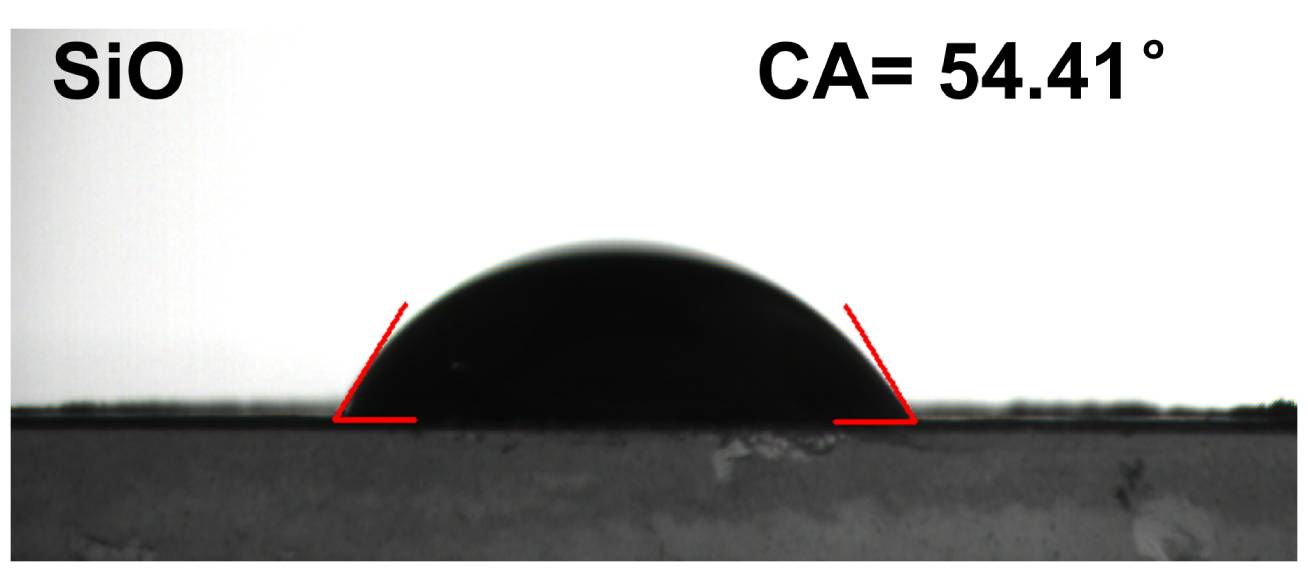
**

**Figure S38.** The contact angle with water for 10 min of SiO electrode.

**
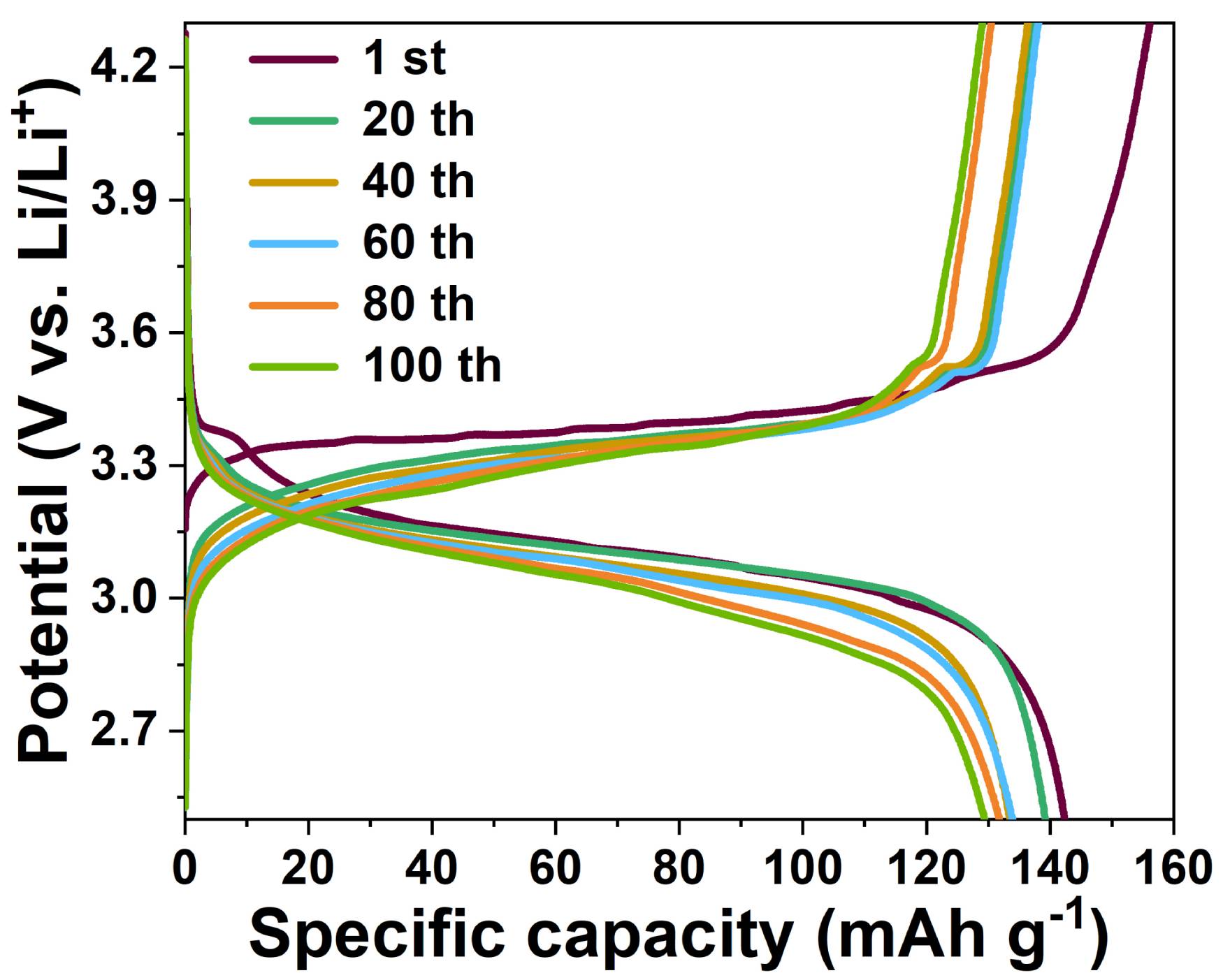
**

**Figure S39.** Charge/discharge curves of LFP//Ah-Pr-SiO.


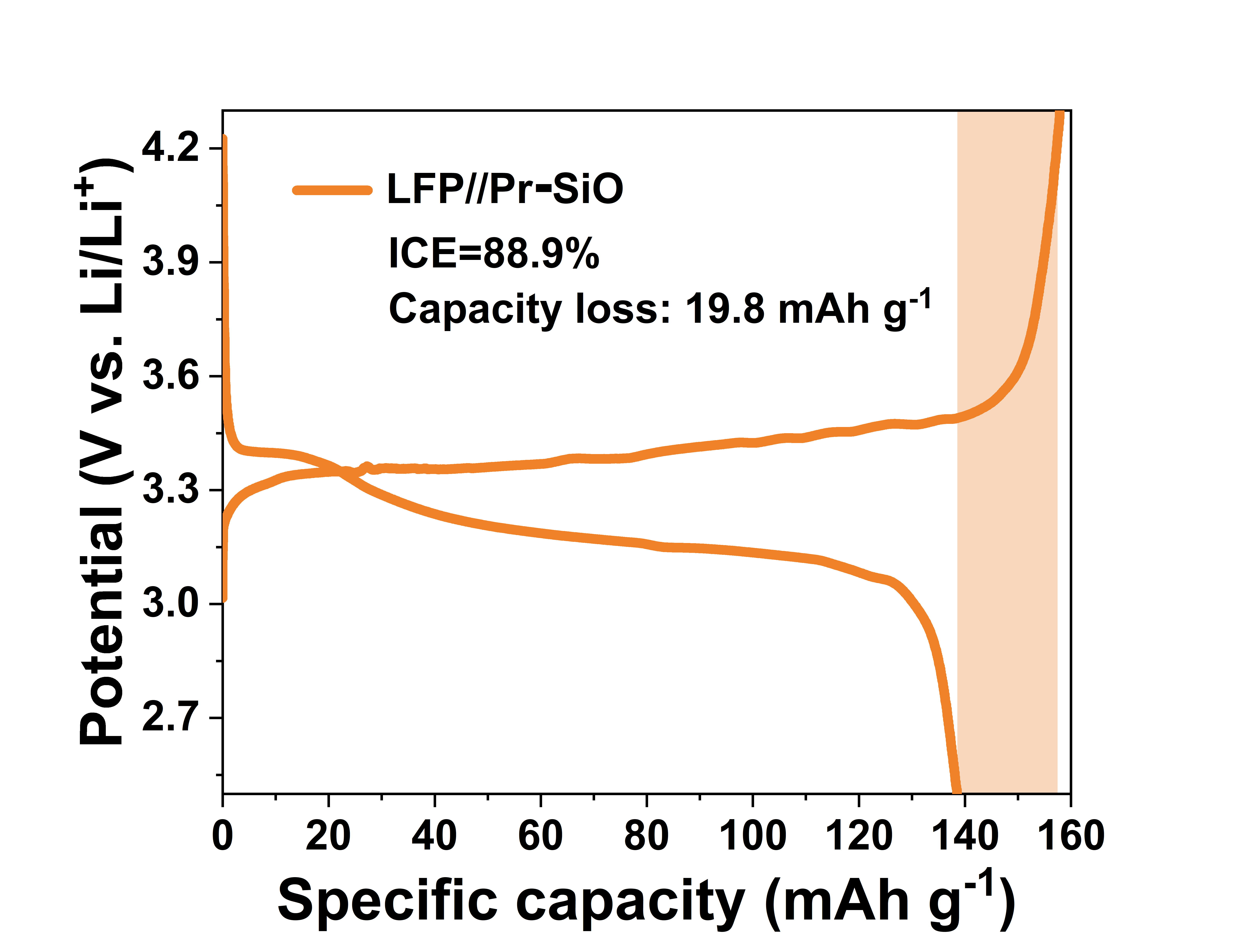


**Figure S40.** The first charge/discharge curves of LFP//Pr-SiO.


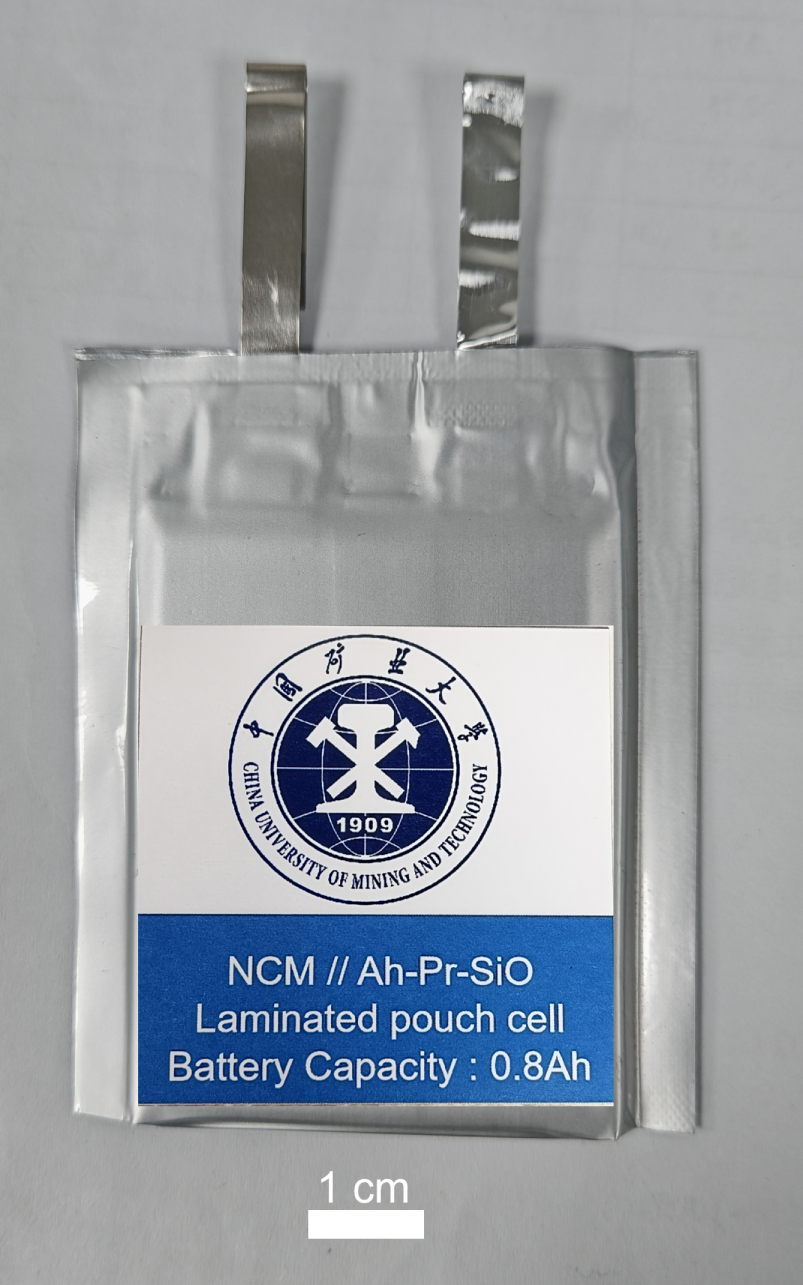


**Figure S41.** Optical image of NCM811//Ah-Pr-SiO pouch cell.

.
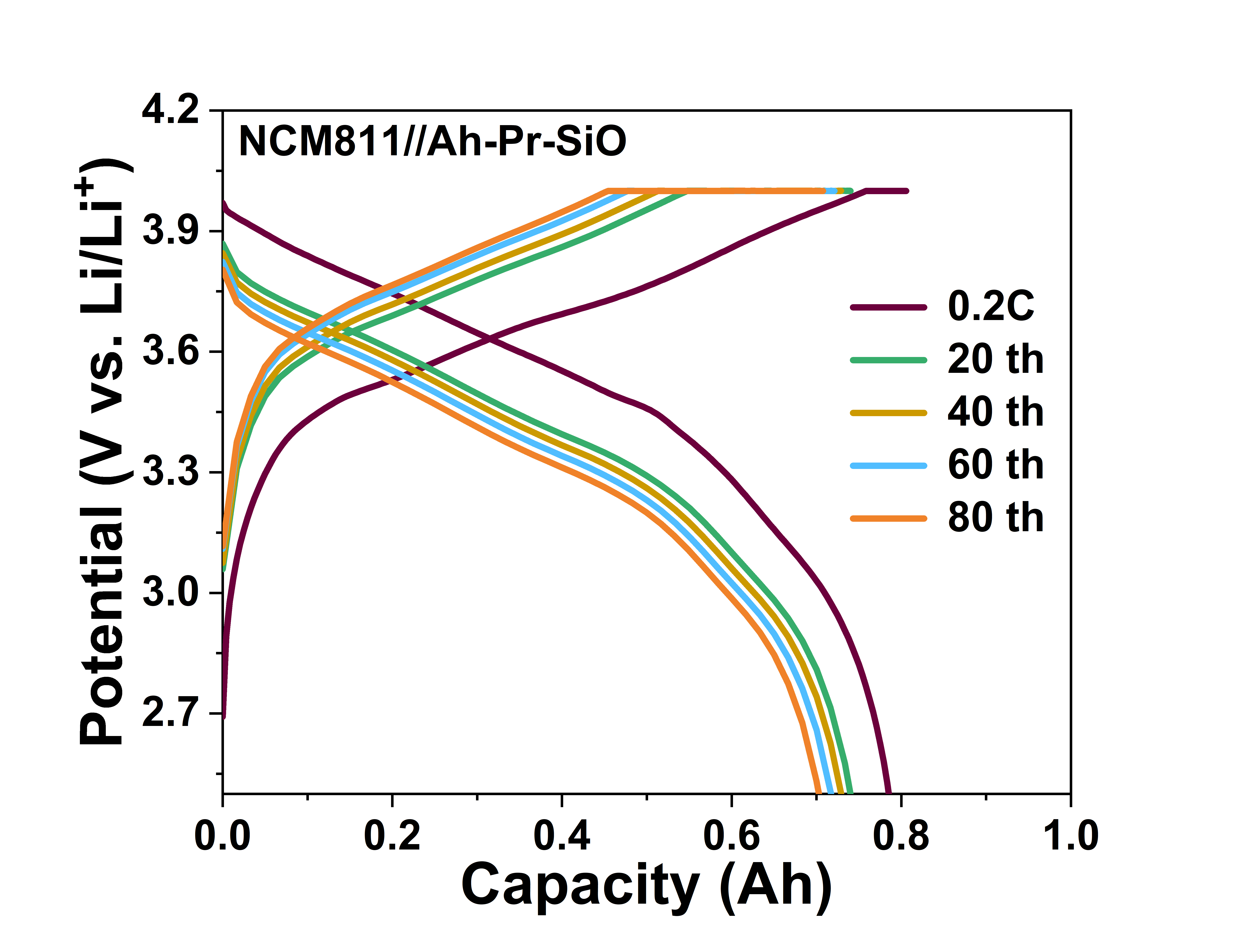


**Figure S42.** Charge/discharge curves of NCM811//Ah-Pr-SiO pouch cell.

**Supporting Equations**

**The fitted values of the Rsei for Figure 3f, g, according to the Arrhenius equation:**

**Equation S1:**$\frac{T}{R_{SEI}}=Ae\text{-}\frac{E_{a}}{RT}$

Where *T*, *R_SEI_*, *A*, *E_a_* and *R* represent the absolute temperature in kelvin, the resistance of Li^+^ through the SEI, pre-exponential factor, activation energy, and standard gas constant, respectively. The activation energy (*E_a_*) is determined by fitting the semicircles (*R_SEI_*) observed in the high-to-middle frequency range of the symmetric batteries. *R_SEI_* specifically characterizes the resistance encountered by Li^+^ when passing through the SEI layer.

**Diffusion coeficient of Li^+^ from GITT results:**

**Equation S2:**

Where *m*_B_, *V_M_*, *M_B_*, *S*, and *τ* are active material mass, molar volume, molar mass, area, and relaxation time.^[2,3]^

**Relationship between the peak current (i) and scan rate (v) of CV curves:**

**Equation S3:**$i\left(V\right)=av^{b}\left(0.5\leq b\leq1\right)$

Where both *a* and *b* are adjustable parameters. The value of *b* is calculated by the slope of log(*i*) vs. log(*ν*)^[2,3]^.

**Relationship of diffusion control and Pseudocapacitive control in CV curves:**

**Equation S4:**

Where represents the contribution of capacitance behavior, and *k*_1_ and *k*_2_ could be obtained by slope and intercept of the straight line.^[2,3]^

**The energy densities (E) of full batteries:**

**Equation S5:**$E_{g}=\frac{C*V}{M_{c}+M_{a}+M_{Al}+M_{Cu}+M_{Se}+M_{e}}$

*E*_g_, stands for the gravimetric-energy-density (Wh kg^-1^), *C* is the capacity (mAh) of full cell, *V* is the actual output voltage (mV), *M_c_* is the cathode mass (mg) which consists of the mass of LFP (80 wt%), conductive carbon (10 wt%) and binder (10 wt%). *M_a_*, is the anode mass (mg) which consists of the mass of SiO (70%), conductive carbon(20%) and binder (10%). *M_Al_*, *M_Cu_*, and *M_se_* stand for the mass of Al current collector, Cu current collector and separator. *M_e_* stands for the mass of electrolyte used in the full cell. It should be noted that the weight of package is not included in the evaluation process^[4]^.

**Supporting Tables**

**Table. S1.** Cost comparison of commonly used inorganic membrane additives.

| Chemicals | Cost ($/KG) | Purity (%) |
| --- | --- | --- |
| Ah | ~47.4 | ≥99 |
| FEC | ~118.2 | ≥98 |
| LiNO_3_ | ~152.9 | ≥99 |

Data sourced from Aladdin Reagent Network.

**Table. S2.** The EIS values of SiO, Pr-SiO and Ah-Pr-SiO anodes.

| Sample | R_SEI_ (Ω) | R_ct_ (Ω) |
| --- | --- | --- |
| SiO | 27.2 | 13.44 |
| Pr-SiO | 17.86 | 10.27 |
| Ah-Pr-SiO | 11.94 | 4.637 |

**Table. S3.** Comparison of the electrochemical performance of prelithiated SiO-based anode materials.

| ICE (%) | Capacity retention (%) | Cycle number | Ref. |
| --- | --- | --- | --- |
| 99.4 | 86.7 | 200 cycles | This work |
| 94.5 | 77.4 | 200 cycles | (8)[5] |
| 89.9 | 42.9 | 200 cycles | (5)[6] |
| 82.6 | 67.2 | 100 cycles | (1)[7] |
| 93.2 | 27.3 | 55 cycles | (2)[8] |
| 90.3 | 45.2 | 100 cycles | (3)[9] |
| 78.3 | <40 | 200 cycles | (4)[10] |
| 88.7 | 54.7 | 200 cycles | (6)[11] |
| 88.5 | ＜71 | 200 cycles | (7)[12] |
| 90.5 | ＜83.3 | 200 cycles | (9)[13] |

**Table. S4.** The atomic percentage (%) of the SEI element signals from SiO, Pr-SiO and Ah-Pr-SiO electrode by the XPS depth profiling test.

| SiO | 0 s | 400 s | 800 s |
| --- | --- | --- | --- |
| C | 44.72 | 41.63 | 39.82 |
| F | 18.17 | 19.57 | 22.53 |
| O | 37.11 | 38.8 | 37.65 |
| Si | 0.32 | 0.52 | 2.67 |

| Pr-SiO | 0 s | 400 s | 800 s |
| --- | --- | --- | --- |
| C | 42.9 | 41.75 | 38.44 |
| F | 16.63 | 19.67 | 21.57 |
| O | 40.24 | 38.12 | 37.89 |
| Si | 0.23 | 0.45 | 2.1 |

| Ah-Pr-SiO | 0 s | 400 s | 800 s |
| --- | --- | --- | --- |
| C | 50.28 | 31.32 | 28.45 |
| F | 9.37 | 22.36 | 24.86 |
| N | 0.53 | 0.58 | 0.64 |
| O | 39.42 | 42.66 | 42.3 |
| Si | 0.29 | 0.45 | 0.78 |
| Zr | 0.1 | 2.63 | 2.96 |

**Table. S5.** Specific parameters of NCM811//Ah-Pr-SiO pouch cells.

| Samples | Parameter | Value |
| --- | --- | --- |
| NCM811 Cathode | Specific capacity | 190 mAh g^-1^ |
|  | Area weight (each side) | 1.89 mAh cm^-2^ |
|  | Area capacity (each side) | 10.5 mg cm^-2^ |
|  | Number of layers | 11 |
| Al foil | thickness | 12 μm |
| Ah-Pr-SiO anode | Specific capacity | 1600 mAh g^-1^ |
|  | Area weight (each side) | 1.89 mg cm^-2^ |
|  | Area capacity (each side) | 2.11 mAh cm^-2^ |
|  | Number of layers | 12 |
| Cu foil | Thickness | 10 μm |
| Separator | Thickness | 1 μm |
| Electrolyte | E/C ratio | 4.5 g Ah^-1^ |
|  | Average Voltage | 3.25 V |
| Pouch cell | Capacity | 1 Ah |
|  | Energy Energy | 371 Wh kg^-1^ |

The full pouch cells were tested in the voltage range of 2.5–4.0 V. Energy density was calculated based on the **Equation S5**.

**Table. S6.** Comparison of the energy density of this work in this study with that of other reported SiO-based lithium-ion full cells.

| Battery | Energy density (Wh kg^-1^)  (based on total mass) | Reference |
| --- | --- | --- |
| NCM811//Ah-Pr-SiO | 346.6 | This work |
| LFP//Ah-Pr-SiO | 268.6 | This work |
| SC-NCM811//SiO-C | 225 | [14] |
| NCM622//SiOx/TiO2@MLG | 300 | [15] |
| NCM811//SiOx/G|LFO-FS| | 330 | [16] |
| NCM811//SiGC-19/Gr | 353 | [17] |

**References**

[1] J. Huang, N. P. Sullivan, A. Zakutayev, R. O Hayre, *Electrochim. Acta* **2023**, *443*, 141879.

[2] Y. Zhang, B. Wu, J. Bi, X. Zhang, D. Mu, X. Y. Zhang, L. Zhang, Y. Xiao, F. Wu, *Carbon Energy* **2024**, *6,* e480.

[3] Q. Man, H. Shen, C. Wei, B. Xi, S. Xiong, J. Feng, *J. Energy Chem.* **2024**, *92*, 224.

[4] Y. Ye, L. Chou, Y. Liu, H. Wang, H. K. Lee, W. Huang, J. Wan, K. Liu, G. Zhou, Y. Yang, A. Yang, X. Xiao, X. Gao, D. T. Boyle, H. Chen, W. Zhang, S. C. Kim, Y. Cui, *Nat. Energy* **2020**, *5*, 786.

[5] Y. Sun, K. Zhang, R. Chai, Y. Wang, X. Rui, K. Wang, H. Deng, H. Xiang, *Adv. Funct. Mater*. **2023**, *33,* 2303020.

[6] X. Hu, P. Xu, M. Liao, X. Lu, G. Shen, C. Zhong, M. Zhang, Q. Huang, Z. Su, *Acs Appl. Energ. Mater*. **2024**, *7* 774-784.

[7] X. Li, Z. Yan, S. Yi, J. Jiang, D. Yang, N. Du, *J. Power Sources* **2023**, *570*, 233021.

[8] C. Kuo, H. Hsu, C. Lan, *J. Power Sources* **2023**, *558,* 232599.

[9] S. Ji, R. Song, H. Yuan, D. Lv, L. Yang, J. Luan, D. Wan, J. Liu, C. Zhong, *J. Electroanal. Chem*. **2024**, *959,* 118141.

[10] Y. Zhang, G. Guo, C. Chen, Y. Jiao, T. Li, X. Chen, Y. Yang, D. Yang, A. Dong, *J. Power Sources* **2019**, *426*, 116-123.

[11] C. Bian, R. Fu, Z. Shi, J. Ji, J. Zhang, W. Chen, X. Zhou, S. Shi, Z. Liu, *Acs Appl. Mater*.*Interfaces* **2022**, *14*, 15337-15345.

[12] D.J. Chung, D. Youn, S. Kim, D. Ma, J. Lee, W.J. Jeong, E. Park, J. Kim, C. Moon, J.Y. Lee, H. Sun, H. Kim, *Nano Energy* **2021**, 89, 106378.

[13] W.J. Jeong, D.J. Chung, D. Youn, N.G. Kim, H. Kim, *Energy Storage Mater.* **2022**, *50*, 740-750.

[14] X. Fan, G. Hu, B. Zhang, X. Ou, J. Zhang, W. Zhao, H. Jia, L. Zou, P. Li, Y. Yang, *Nano Energy* **2020**, *70*, 104450.

[15] H. Xue, Y. Wu, Y. Zou, Y. Shen, G. Liu, Q. Li, D. Yin, L. Wang, J. Ming, *Adv. Funct. Mater.* **2020**, *30*, 1910657.

[16] Q. Meng, M. Fan, X. Chang, H. Li, W.P. Wang, Y.H. Zhu, J. Wan, Y. Zhao, F. Wang, R. Wen, S. Xin, Y.G. Guo, *Adv. Energy Mater.* **2023**, *13*, 2300507.

[17] R. Fu, J. Ji, L. Yun, Y. Jiang, J. Zhang, X. Zhou, Z. Liu, *Energy Storage Mater.* **2021**, *35*, 317-326.
